# Supplementary material for: A specialized bone marrow microenvironment for fetal haematopoiesis
Source: Nat Commun. 2022 Mar 14;13:1327. doi: 10.1038/s41467-022-28775-x (PMC8921288; doi:10.1038/s41467-022-28775-x)
Supplement: Supplementary file 1 — Supplementary Information [file 41467_2022_28775_MOESM1_ESM.pdf]

Supplementary Information for

A specialized bone marrow microenvironment for fetal haematopoiesis

Yang Liu[#] [*], Qi Chen[#], Hyun-Woo Jeong[#], Bong Ihn Koh, Emma C. Watson, Cong Xu, Martin Stehling, Bin Zhou, Ralf H. Adams[*]

[*]Corresponding author Emails:

ralf.adams@mpi-muenster.mpg.de

yangl005@scut.edu.cn

The Supplementary Information contains the following data

- Supplementary Tables

Supplementary Table 1. Summary of genetic mouse models used in this study
Supplementary Table 2. Summary of primary antibodies used in this study

- Supplementary Figures

Supplementary Fig. 1 | Vascular development in fetal BM.
Supplementary Fig. 2 | Fetal BM development and properties of c-Kit hematopoietic cells.
Supplementary Fig. 3 | Additional data for scRNA-seq analysis of all cells and HSPCs.
Supplementary Fig. 4 | Additional analysis of BMSCs
Supplementary Fig. 5 | Analysis of ECs and relationship between arteries and HSPCs.
Supplementary Fig. 6 | scRNA-seq analysis of embryonic and adult ECs
Supplementary Fig. 7 | Additional analysis of $Wls^{i\Delta Bmx}$ mutants.
Supplementary Fig. 8 | Analysis of $Wls^{i\Delta Bmx}$ mutant liver.
Supplementary Fig. 9 | Normal hematopoietic cell expansion and nerve extension in $Wls^{i\Delta Myh11}$ mutants.
Supplementary Fig. 10 | Femoral nerves are not required for fetal HSPC development.
Supplementary Fig. 11 | Additional analysis of Ctnnb1 inactivation in hematopoietic cells.
Supplementary Fig. 12 | Effect of IWR-1 on HSPCs.
Supplementary Fig. 13 | Additional data about the effect of Wnt on HSPCs.

Supplementary Table S1. Summary of genetic mouse models used in this study

Full Name	Short name	Inducible	Source	Purpose
Rosa26-mT/mG	*R26-mTmG*		Jackson Lab. Stock No. 007676	Cre reporter, labeling of Cre-recombined cells and their descendants
Vav1-Cre	-	No	Jackson Lab. Strain No. 008610	Labeling of and gene knockout in hematopoietic cells and their descendants
Lepr-Cre	-	No	Jackson Lab. Strain No. 008320	Labeling of and gene knockout in LepR+ stromal cells and their descendants
Wnt1-Cre	-	No	Jackson Lab. Strain No. 007807	Labeling of and gene knockout in nerve and their descendants
Bmx-CreERT2	-	Yes	generated by Adams group	Labeling of and gene knockout arterial endothelial cells and their descendants
Myh11-CreERT2	-	Yes	Jackson Lab. Stock No. 019079	Labeling of and gene knockout vascular smooth muscle cells and their descendants
Kit-CreERT		Yes	generated by Bin Zhou, Shanghai	Labeling of and gene knockout c-Kit+ cells and their descendants
Wntless floxed	*Wls*		Jackson Lab. Stock No. 012888	Conditional knockout of *Wntless* to block Wnt secretion from Cre+ cells
Ctnnb1 floxed			Jackson Lab. Stock No. 004152	Conditional knockout of *Ctnnb1*
Bmx-CreERT2 Wntless	$Wls^{i\Delta Bmx}$	Yes	see above	Conditional knockout of *Wntless* to block Wnt secretion from arterial endothelial cells
Myh11-CreERT2 Wntless	$Wls^{i\Delta Myh11}$	Yes	see above	Conditional knockout of *Wntless* to block Wnt secretion from vascular smooth muscle cells
Wnt1-Cre Ctnnb1	$Ctnnb1^{\Delta Wnt1}$	No	see above	Conditional knockout of *Ctnnb1* in nerve
Vav1-Cre Ctnnb1	$Ctnnb1^{\Delta Vav1}$	No	see above	Conditional knockout of *Ctnnb1* in hematopoietic cells
Kit-CreER Ctnnb1	$Ctnnb1^{i\Delta Kit}$	Yes	see above	Conditional knockout of *Ctnnb1* in c-Kit+ hematopoietic cells and their descendants
Bmx-CreERT2 Rosa26-mT/mG	*Bmx-mTmG*	Yes	see above	Reporter and genetic lineage tracing of arterial endothelial cells and their descendants
Lepr-Cre Rosa26-mT/mG	*Lepr-mTmG*	No	see above	Reporter and genetic lineage tracing of LepR+ stromal cells and their descendants

Efnb2-GFP			Jackson Lab. Stock No. 007843	Genetic labelling of EphrinB2+ arterial endothelial cells with nuclear H2B-GFP

Supplementary Table 2 | Summary of primary antibodies used in this study

Primary antibodies	Company	Catalog	Dilution	Clone	Usage
Endomucin	Santa Cruz	SC-65495	1:100	V.7C7	IHC
CD31	R&D	AF3628	1:100	-	IHC
CD31	BD pharmingen	553370	1:100	MEC13.3	IHC
Osterix	Abcam	Ab22552	1:800	-	IHC
Caveolin-1	Cell signaling	3238	1:100	-	IHC
GFP-Alexa488	Invitrogen	A21311	1:100	-	IHC
GFP	Aveslabs	GFP-1010	1:400	-	IHC
Th	Millipore	AB152	1:50	-	IHC
αSMA-eFluor660	eBioscience	50-9760-82	1:50	1A4	IHC
αSMA-cy3	Sigma	C6198	1:100	1A4	IHC
Synaptophysin	Abcam	Ab32594	1:50	-	IHC
Lineage-biotin cocktail	BD pharmingen	559971	1:100	-	IHC
c-Kit	R&D	AF1356	1:100	-	IHC
c-Kit-FITC	Biolegend	105806	1:50	2B8	IHC
Tuj1	Abcam	ab107216	1:50	-	IHC
Tuj1-488	Biolegend	657404	1:50	AA10	IHC
TrkA	R&D	AF1056	1:100	-	IHC
CyclinD1	Cell signaling	2978	1:200	92G2	IHC
CD150-FITC	ThermoFisher	11-1501	1:100	9D1	IHC
CD150	AbD Serotec	MCA2274A488	1:100	9D1	IHC
PDGFRβ	Abcam	ab32570	1:200	Y92	IHC
CD41-biotin	ThermoFisher	eBioMWReg30	1:100	13-0411	IHC
CD48-biotin	ThermoFisher	13-0481	1:100	HM48-1	IHC
Col3a1	Novusbio	NB600-594	1:200		IHC
Lineage-biotin cocktail	BD pharmingen	559971	20 μl per $1x10^6$ cells		FACS
Lineage-biotin cocktail-APC	BD pharmingen	558074	20 μl per $2x10^6$ cells		FACS
Sca1-FITC	eBioscience	11-5981-85	6 μl per $2x10^6$ cells	D7	FACS
Sca1-PE-Cy7	BD pharmingen	558162	6 μl per $2x10^6$ cells	D7	FACS
c-Kit-APC	BD pharmingen	553356	3 μl per $2x10^6$ cells	2B8	FACS

c-Kit-FITC	Biolegend	105806	8 µl per $2x10^6$ cells	2B8	FACS
Ter119-APC	eBioscience	17-5921	4 µl per $1x10^6$ cells	Ter-119	FACS
CD45-FITC	eBioscience	11-0451	4 µl per $1x10^6$ cells	30-F11	FACS
CD45R(B220)-APC	Invitrogen	RM2605	3 µl per $1x10^6$ cells	RA3-6B2	FACS
Gr-1-APC	eBioscience	17-5931	3 µl per $1x10^6$ cells	RB6-8C5	FACS
CD11b-APC	Biolegend	101212	3 µl per $1x10^6$ cells	M1/70	FACS
CD11b-FITC	BD pharmingen	553310	3 µl per $1x10^6$ cells	M1/70	FACS
CD150-PE	Biolegend	115904	4 µl per $2x10^6$ cells	TC15-12F12.2	FACS
CD48-APC-Cy7	BD pharmingen	561242	4 µl per $2x10^6$ cells	HM48-1	FACS
CD34-eFluor450	eBioscience	48-0341	4 µl per $2x10^6$ cells	RAM34	FACS
CD127-PE	eBioscience	12-1271	4 µl per $2x10^6$ cell	A7R34	FACS
CD16/CD32 PE-Cy7	eBioscience	25-0161	4 µl per $2x10^6$ cells	93	FACS
Ter-119 Pacific Blue	Biolegend	116232	4 µl per $1x10^6$ cells	Ter-119	FACS
CD45- Pacific Blue	Biolegend	103126	4 µl per $1x10^6$ cells	30-F11	FACS
CD45.1-FITC	Invitrogen	MCD45101	4 µl per $1x10^6$ cells	A20	FACS
CD45.2 Pacific Blue	Biolegend	109820	4 µl per $1x10^6$ cells	104	FACS
CD45 PE-Cy7	eBioscience	25-0451-82	4 µl per $1x10^6$ cells	30-F11	FACS

Ki67-FITC	Biolegend	652410	4 µl per $1x10^6$ cells	16A8	FACS
CD71-Biotin	Biolegend	113803	10 µl per 10^7 cells	RI7217	Cell depletion
CD117	Miltenyi Biotec	130-091-224	10µl per 10^7 cells		Cell depletion
CD45	Miltenyi Biotec	130-052-301	10 µl per 10^7 cells		Cell depletion
Ter119	Miltenyi Biotec	130-049-901	10 µl per 10^7 cells		Cell depletion

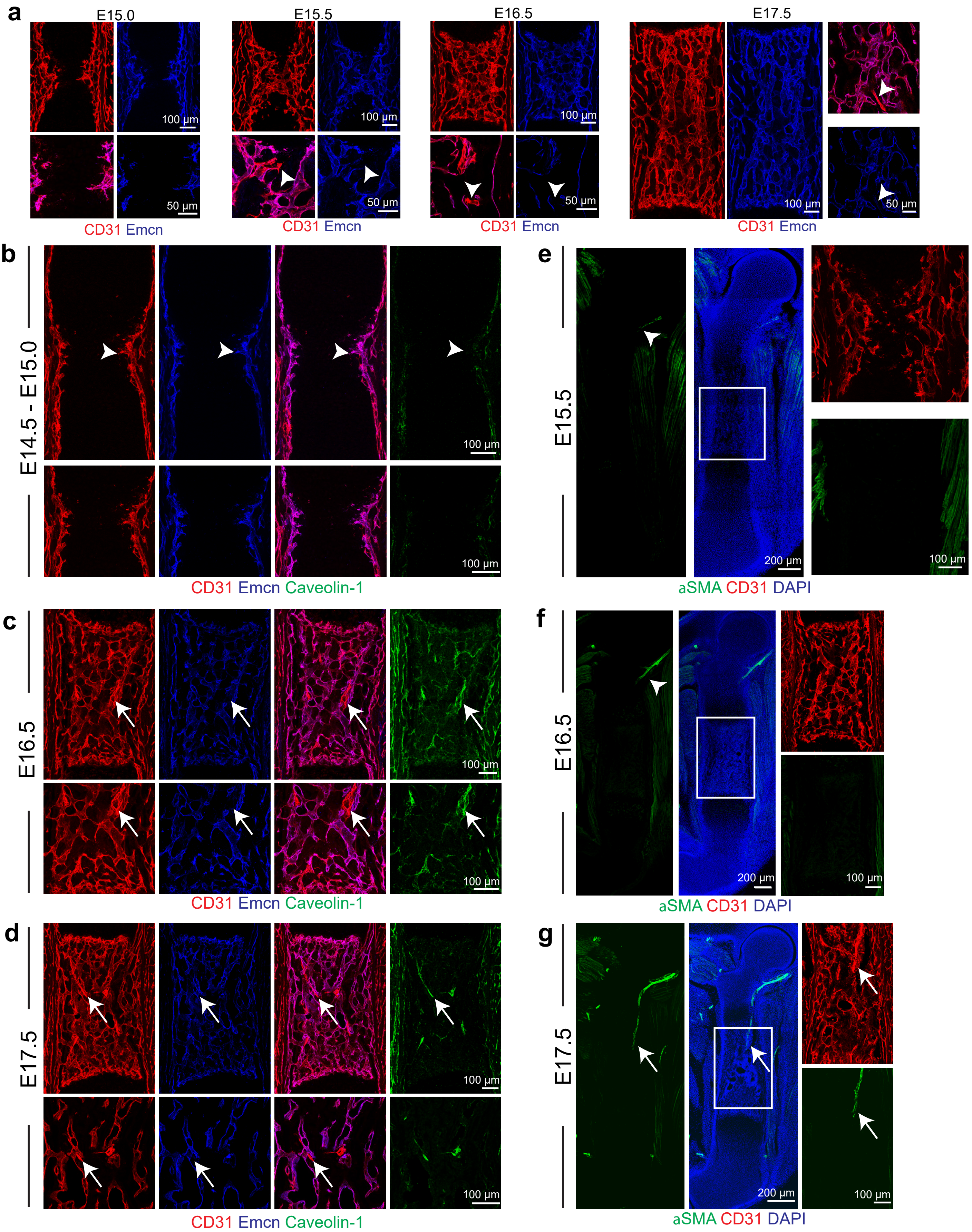

a
E15.0
E15.5
E16.5
E17.5
100 µm
50 µm
CD31 Emcn
b
E14.5 - E15.0
100 µm
CD31 Emcn Caveolin-1
c
E16.5
CD31 Emcn Caveolin-1
d
E17.5
CD31 Emcn Caveolin-1
e
E15.5
200 µm
aSMA CD31 DAPI
f
E16.5
aSMA CD31 DAPI
g
E17.5
aSMA CD31 DAPI

Supplementary Fig. 1 | Vascular development in fetal BM.

(**a**) Representative overview and high magnification images of artery development in femur at the indicated stages. Arrowheads mark $CD31^+$ $Emcn^-$ arteries.
(**b-d**) Expression pattern of AEC marker Caveolin-1 together with CD31 and Emcn at E15.0 (b), E16.5 (c), and E17.5 (d). Arrowhead in (b) marks Caveolin-1-negative primitive vascular plexus. Arrows in (c, d) indicate Caveolin-1^+ $CD31^+$ $Emcn^-$ AECs.
(**e-g**) Overview and high magnification images showing αSMA expression during artery development in femur. αSMA signals are undetectable at E15.5 (e) and E16.5 (f) but decorate the trochanter artery at E17.5 (g) inside femur. Arrowheads indicate αSMA signals outside femur, arrows mark the $αSMA^+$ trochanter artery penetrating into the bone.

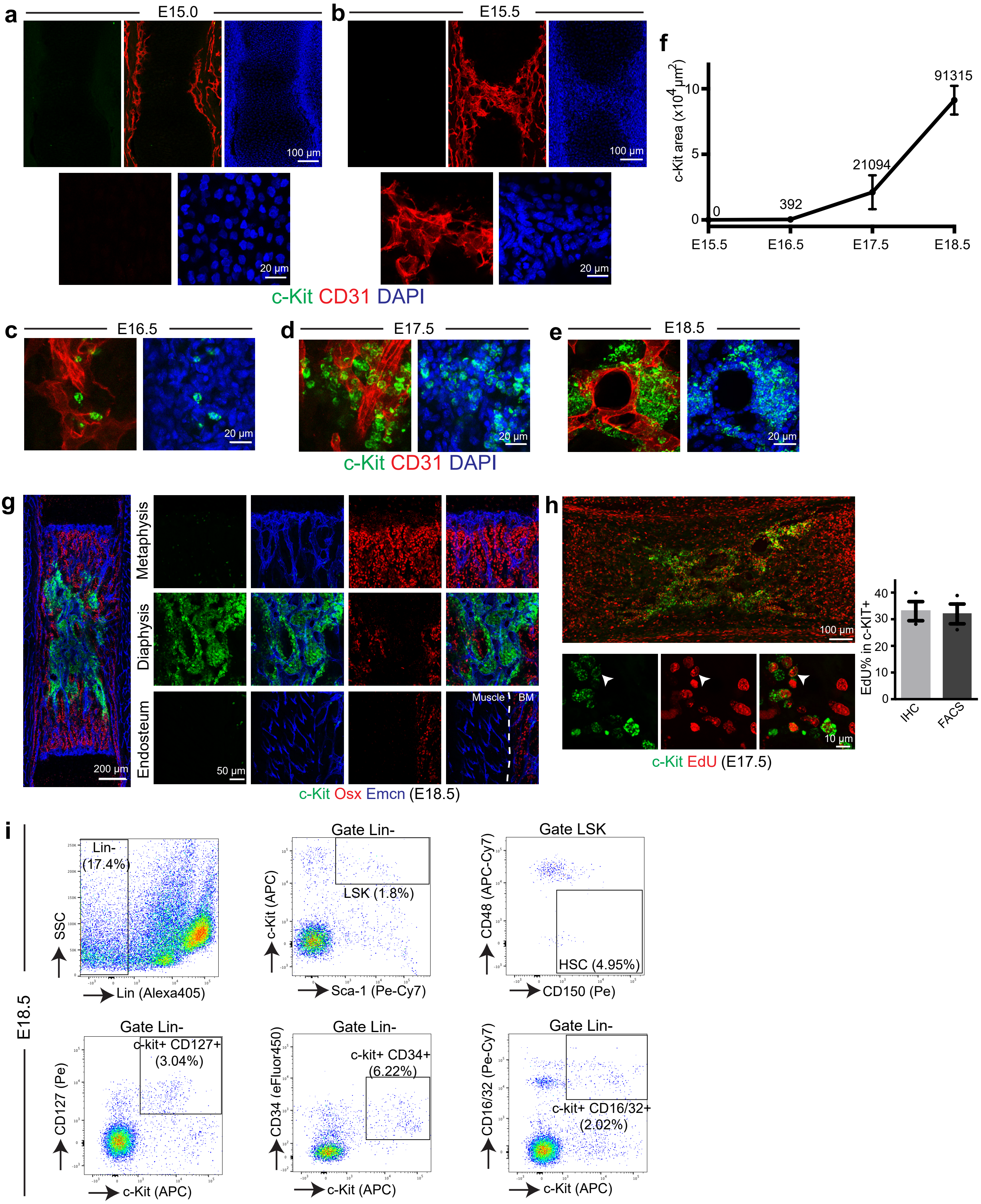
a
E15.0
100 µm
20 µm
b
E15.5
100 µm
20 µm
c-Kit CD31 DAPI
f
c-Kit area (x10^4 µm^2)
91315
21094
392
0
E15.5
E16.5
E17.5
E18.5
c
E16.5
20 µm
d
E17.5
20 µm
e
E18.5
20 µm
c-Kit CD31 DAPI
g
Metaphysis
Diaphysis
Endosteum
200 µm
50 µm
Muscle
BM
c-Kit Osx Emcn (E18.5)
h
100 µm
10 µm
EdU% in c-KIT+
IHC
FACS
c-Kit EdU (E17.5)
i
E18.5
Lin-
(17.4%)
SSC
Lin (Alexa405)
Gate Lin-
LSK (1.8%)
c-Kit (APC)
Sca-1 (Pe-Cy7)
Gate LSK
HSC (4.95%)
CD48 (APC-Cy7)
CD150 (Pe)
Gate Lin-
c-kit+ CD127+
(3.04%)
CD127 (Pe)
c-Kit (APC)
Gate Lin-
c-kit+ CD34+
(6.22%)
CD34 (eFluor450)
c-Kit (APC)
Gate Lin-
c-kit+ CD16/32+
(2.02%)
CD16/32 (Pe-Cy7)
c-Kit (APC)

Supplementary Fig. 2 | Fetal BM development and properties of c-Kit hematopoietic cells.

(**a-e**) Representative overview and high magnification images showing c-Kit$^+$ cells in femoral BM at E15.0 (a), E15.5 (b), E16.5 (c), E17.5(d), and E18.5 (e).
(f) Quantification of c-Kit$^+$ cell covered area in femur at different developmental stages. Numbers indicate average value (E16.5 N=3; E15.5, E17.5, E18.5 N=4 embryos for each stage). Error bars, mean±s.e.m.
(g) Representative overview and high magnification images of c-Kit$^+$ cell distribution in femur at E18.5.
(h) Representative overview and high magnification images of c-Kit together with EdU signal in E17.5 BM (arrowhead marks c-Kit$^+$ EdU$^+$ cell). Quantification of EdU% in c-Kit cells by immunostaining and FACS. IHC=3, FACS=3. Error bars, mean±s.e.m.
(i) Representative FACS gating of Lin$^-$, LSK, HSC, Lin$^-$ c-Kit$^+$ CD127$^+$, Lin- c-Kit$^+$ CD34$^+$, Lin$^-$ c-Kit$^+$ CD16/32$^+$ cells.

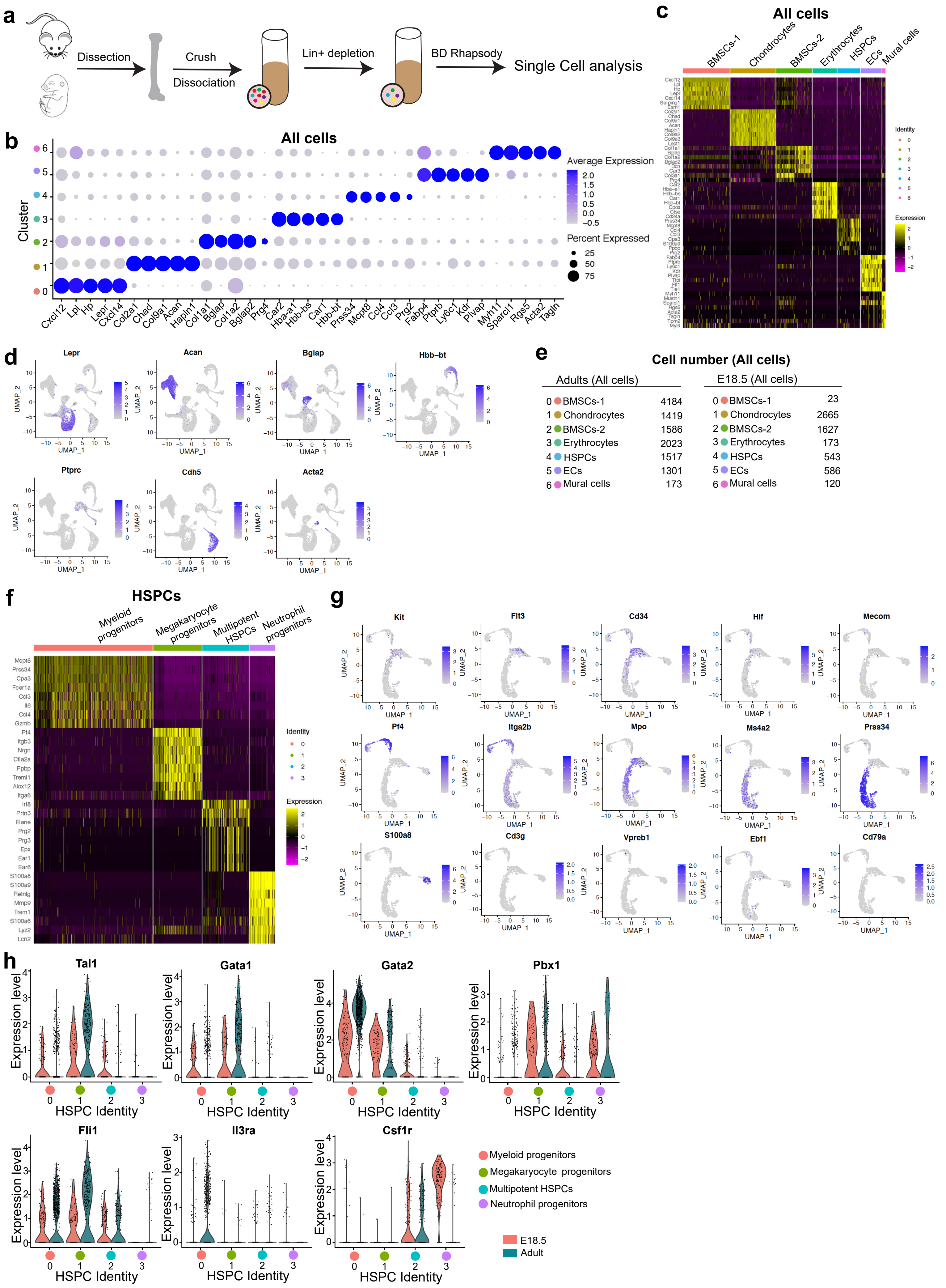
a
Dissection
Crush
Dissociation
Lin+ depletion
BD Rhapsody
Single Cell analysis
b
All cells
Average Expression
Percent Expressed
Cluster
c
All cells
BMSCs-1
Chondrocytes
BMSCs-2
Erythrocytes
HSPCs
ECs
Mural cells
Identity
Expression
d
Lepr
Acan
Bglap
Hbb–bt
Ptprc
Cdh5
Acta2
e
Cell number (All cells)
Adults (All cells)
E18.5 (All cells)
0 BMSCs-1 4184
1 Chondrocytes 1419
2 BMSCs-2 1586
3 Erythrocytes 2023
4 HSPCs 1517
5 ECs 1301
6 Mural cells 173
0 BMSCs-1 23
1 Chondrocytes 2665
2 BMSCs-2 1627
3 Erythrocytes 173
4 HSPCs 543
5 ECs 586
6 Mural cells 120
f
HSPCs
Myeloid progenitors
Megakaryocyte progenitors
Multipotent HSPCs
Neutrophil progenitors
g
Kit
Flt3
Cd34
Hlf
Mecom
Pf4
Itga2b
Mpo
Ms4a2
Prss34
S100a8
Cd3g
Vpreb1
Ebf1
Cd79a
h
Tal1
Gata1
Gata2
Pbx1
Fli1
Il3ra
Csf1r
Expression level
HSPC Identity
Myeloid progenitors
Megakaryocyte progenitors
Multipotent HSPCs
Neutrophil progenitors
E18.5
Adult

Supplementary Fig. 3 | Additional data for scRNA-seq analysis of all cells and HSPCs.

(a) Scheme showing magnetic separation for the depletion of mature hematopoietic cells prior to scRNA-seq analysis.
(b) Dot plot showing the top 5 markers for each cluster in all cells. Dot size represents percentage of cells where the gene is detected, color indicates average expression level of the gene in each cluster.
(c) Heat map showing the top 8 markers for each cluster in all cells.
(d) UMAP plots showing distribution of selected markers in all cells. Color represents relative expression level.
(e) Quantification of cell number in each cluster.
(f) Top 8 differentially expressed genes in each HSPC subcluster.
(g) UMAP plots showing distribution of selected markers in each HSPC cluster. Color represents relative expression level.
(h) Violin plots showing expression of important transcription factors and receptors in each HSPC subcluster at E18.5 or in adult.

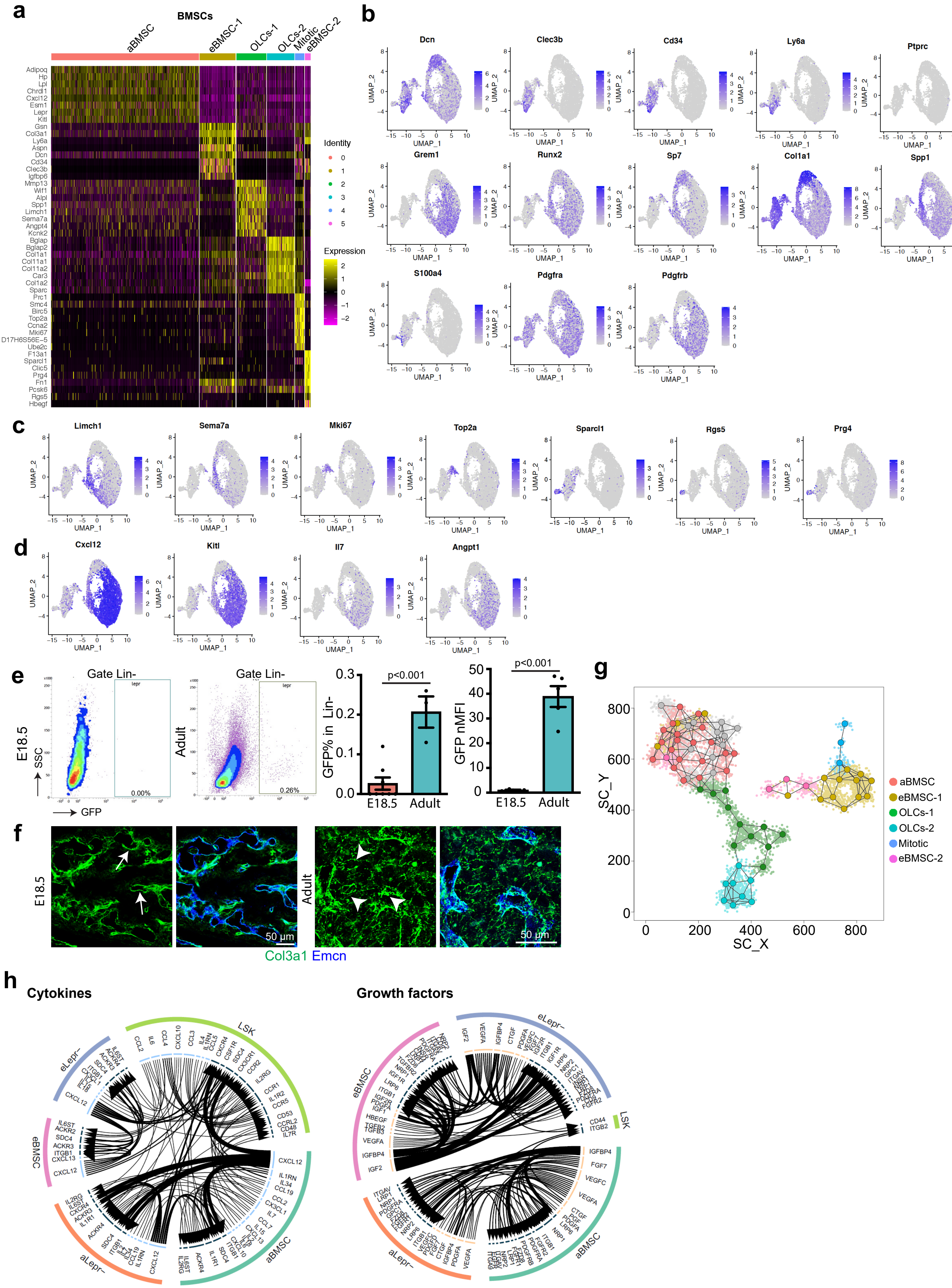

a
BMSCs
aBMSC
eBMSC-1
OLCs-1
OLCs-2
Mitotic
eBMSC-2
Adipoq
Hp
Lpl
Chrdl1
Cxcl12
Esm1
Lepr
Kitl
Gsn
Col3a1
Ly6a
Aspn
Dcn
Cd34
Clec3b
Igfbp6
Mmp13
Wif1
Alpl
Spp1
Limch1
Sema7a
Angpt4
Kcnk2
Bglap
Bglap2
Col1a1
Col11a1
Col11a2
Car3
Col1a2
Sparc
Prc1
Smc4
Birc5
Top2a
Ccna2
Mki67
D17H6S56E–5
Ube2c
F13a1
Sparcl1
Clic5
Prg4
Fn1
Pcsk6
Rgs5
Hbegf
Identity
0
1
2
3
4
5
Expression
b
Dcn
Clec3b
Cd34
Ly6a
Ptprc
Grem1
Runx2
Sp7
Col1a1
Spp1
S100a4
Pdgfra
Pdgfrb
UMAP_1
UMAP_2
c
Limch1
Sema7a
Mki67
Top2a
Sparcl1
Rgs5
Prg4
d
Cxcl12
Kitl
Il7
Angpt1
e
Gate Lin-
E18.5
Adult
SSC
GFP
lepr
0.00%
0.26%
GFP% in Lin-
GFP nMFI
p<0.001
E18.5
Adult
f
E18.5
Adult
50 μm
Col3a1 Emcn
g
SC_Y
SC_X
aBMSC
eBMSC-1
OLCs-1
OLCs-2
Mitotic
eBMSC-2
h
Cytokines
Growth factors
LSK
eLepr–
eBMSC
aLepr–
aBMSC

Supplementary Fig. 4 | Additional analysis of BMSCs

(**a**) Heat map showing the top 8 markers for each BMSC subcluster.
(**b-d**) UMAP plots showing distribution of additional markers and HSPC-supporting factors in BMSC cluster. Color represents relative expression level.
(e) Representative FACS plot showing GFP^+ ($LepR^+$) cells in adult or E18.5 Lin^- Lepr-Cre R26-mTmG BM. Quantification of GFP^+ cell percentage or normalized mean fluorescent inten-sity (nMFI) of GFP+ cells. E18.5=8, Adult=3. Error bars, mean±s.e.m. Two-tailed Student's t test.
(f) High magnification images showing Col3a1 immunostaining together with the EC marker Emcn in E18.5 or adult BM. Arrows indicate vessel-associated Col3a1 signal in fetal BM, arrow-heads mark $Col3a1^+$ reticular fibres in adult BM.
(g) Metacell analysis showing interrelation of BMSC subclusters.
(h) Interactome analysis between multipotent HSPCs and different BMSC clusters. The direc-tion of arrows indicates potential interaction, width of line and arrow represent relative gene expression level.

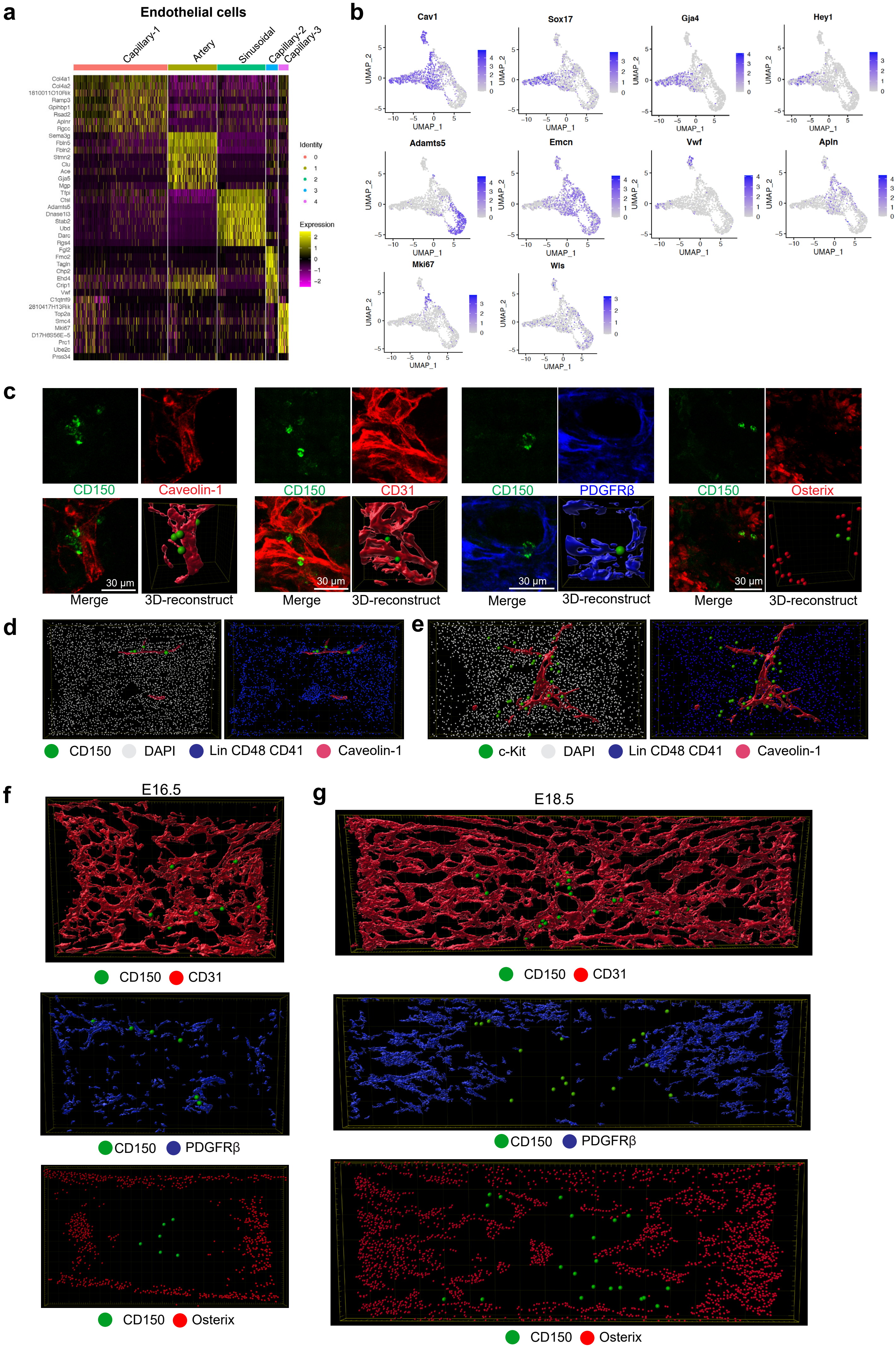
a
Endothelial cells
Capillary-1
Artery
Sinusoidal
Capillary-2
Capillary-3
Identity
Expression
b
Cav1
Sox17
Gja4
Hey1
Adamts5
Emcn
Vwf
Apln
Mki67
Wls
UMAP_1
UMAP_2
c
CD150
Caveolin-1
CD31
PDGFRβ
Osterix
Merge
3D-reconstruct
30 μm
d
CD150
DAPI
Lin CD48 CD41
Caveolin-1
e
c-Kit
f
E16.5
g
E18.5
CD150
CD31
PDGFRβ
Osterix

Supplementary Fig. 5 | Analysis of ECs and relationship between arteries and HSPCs.

(a) Heatmap showing the top 8 differentially expressed genes in each EC subcluster.
(b) UMAP plot showing distribution of selected markers in each EC subclusters. Color represents relative expression level of the gene (RNA).
(c) Comparison of projected images and 3D-reconstruction from original confocal images for CD150.
(**d, e)** Representative 3D-reconstruction image for quantification of the distance to caveolin-1$^+$ arteries for CD150$^+$, DAPI$^+$ or Lin$^+$ CD48$^+$ CD41$^+$ cells (d) or for c-Kit$^+$, DAPI$^+$ and Lin$^+$ CD48$^+$ CD41$^+$ cells (e).
(**f, g**) 3D-reconstruction images showing position of CD150$^+$ cells relative to CD31$^+$ ECs, PDGFRβ$^+$ stromal cells and Osterix$^+$ osteoprogenitor cells at E16.5 (f) or at E18.5 (g).

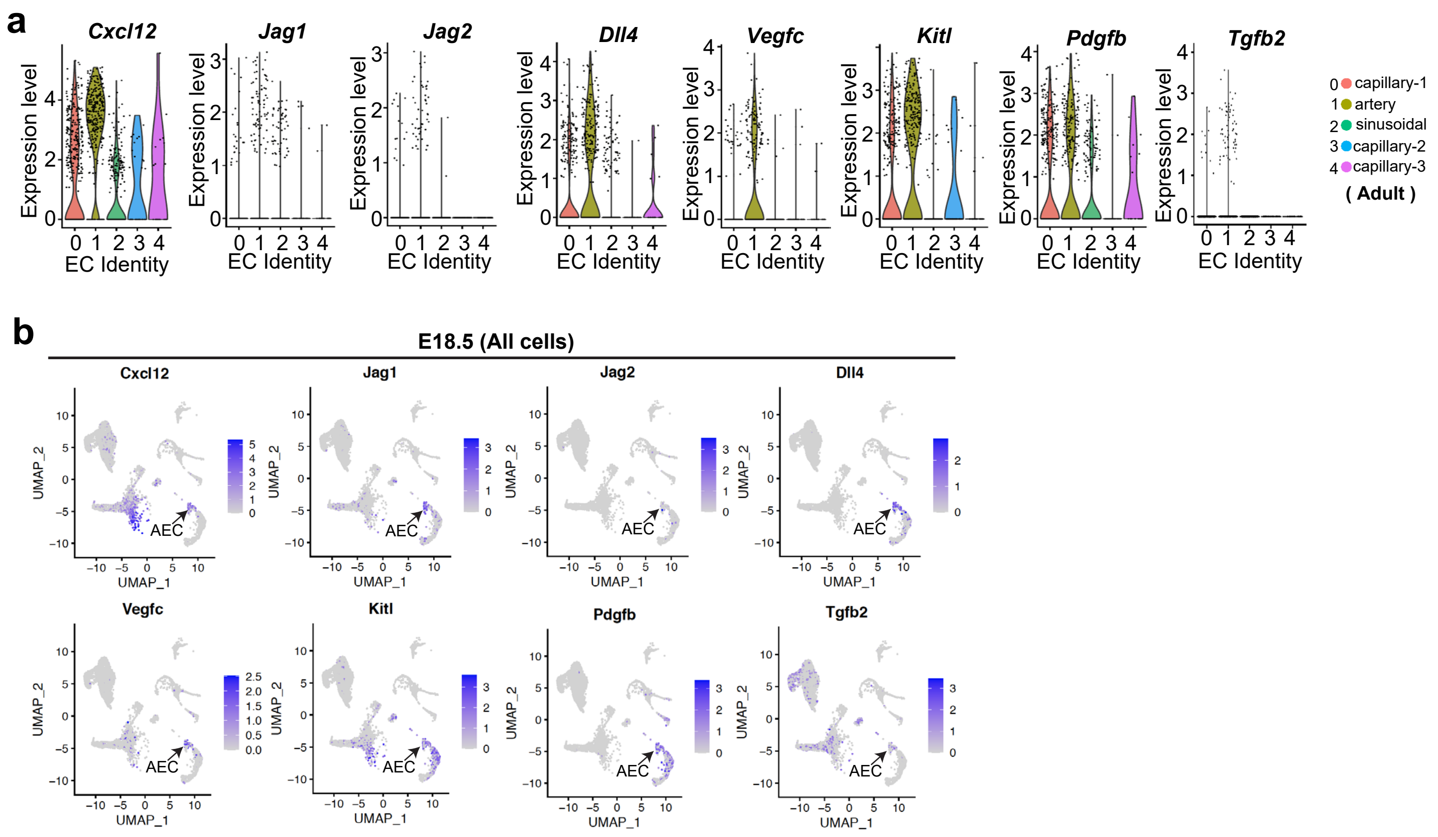
a
Cxcl12
Jag1
Jag2
Dll4
Vegfc
Kitl
Pdgfb
Tgfb2
Expression level
EC Identity
0 capillary-1
1 artery
2 sinusoidal
3 capillary-2
4 capillary-3
(Adult)
b
E18.5 (All cells)
Cxcl12
Jag1
Jag2
Dll4
Vegfc
Kitl
Pdgfb
Tgfb2
AEC
UMAP_1
UMAP_2

Supplementary Fig. 6 | scRNA-seq analysis of embryonic and adult ECs

(a) Violin plots showing expression of angiocrine or HSPC-supporting factors in AECs or other EC subclusters in adult bone.
(b) UMAP plots showing distribution of angiocrine or HSPC supportive factor in all E18.5 cells. Arrows indicate location of AECs, colors represent relative gene expression level.

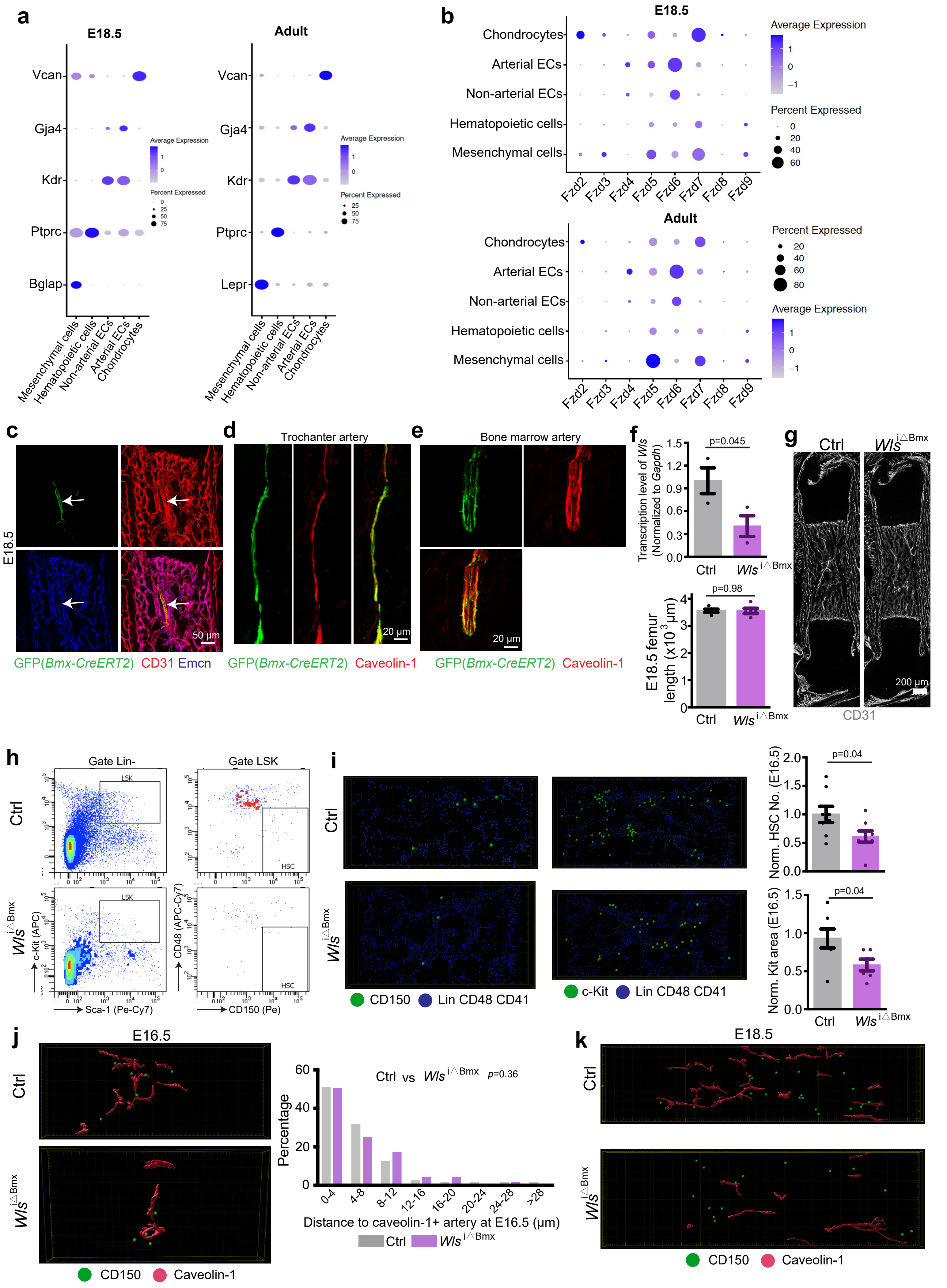
a
E18.5
Adult
Vcan
Gja4
Kdr
Ptprc
Bglap
Lepr
Average Expression
Percent Expressed
Mesenchymal cells
Hematopoietic cells
Non-arterial ECs
Arterial ECs
Chondrocytes
b
E18.5
Adult
Chondrocytes
Arterial ECs
Non-arterial ECs
Hematopoietic cells
Mesenchymal cells
Fzd2
Fzd3
Fzd4
Fzd5
Fzd6
Fzd7
Fzd8
Fzd9
Average Expression
Percent Expressed
c
E18.5
50 µm
GFP(Bmx-CreERT2) CD31 Emcn
d
Trochanter artery
20 µm
GFP(Bmx-CreERT2) Caveolin-1
e
Bone marrow artery
20 µm
GFP(Bmx-CreERT2) Caveolin-1
f
Transcription level of Wls (Normalized to Gapdh)
p=0.045
Ctrl
Wls i△Bmx
E18.5 femur length (x10³ µm)
p=0.98
g
Ctrl
Wls i△Bmx
200 µm
CD31
h
Gate Lin-
Gate LSK
LSK
HSC
Ctrl
Wls i△Bmx
c-Kit (APC)
Sca-1 (Pe-Cy7)
CD48 (APC-Cy7)
CD150 (Pe)
i
Ctrl
Wls i△Bmx
CD150
Lin CD48 CD41
c-Kit
Lin CD48 CD41
Norm. HSC No. (E16.5)
p=0.04
Norm. Kit area (E16.5)
p=0.04
Ctrl
Wls i△Bmx
j
E16.5
Ctrl
Wls i△Bmx
CD150
Caveolin-1
Ctrl vs Wls i△Bmx p=0.36
Percentage
0-4
4-8
8-12
12-16
16-20
20-24
24-28
>28
Distance to caveolin-1+ artery at E16.5 (µm)
Ctrl
Wls i△Bmx
k
E18.5
Ctrl
Wls i△Bmx
CD150
Caveolin-1

Supplementary Fig. 7 | Additional analysis of Wls$^{i\Delta Bmx}$ mutants.

(a) Representative markers showing unsupervised clustering of cells in targeted scRNA-seq at E18.5 and in adult. Dot size indicates percentage of cells where the gene was detected, color reflects average gene expression level in each cluster.
(b) Dot plots showing Frizzled receptor transcripts in different BM cell populations in E18.5 and adult BM based on targeted scRNA-seq.
(**c-e**) Representative confocal images confirming that the tamoxifen-inducible Bmx-CreERT2 allele specifically triggers recombination (GFP expression in R26-mTmG Cre reporter background) in CD31^{+} Emcn^{-} arteries (arrows) in BM (c), caveolin-1^{+} trochanter artery (d) or caveolin-1^{+} artery in BM (e).
(**f**) Quantification of knockout efficiency in Wls$^{i\Delta Bmx}$ artery by qPCR analysis of FACS-sorted CD31^{+} Emcn^{-} AECs from fetal mice (N=3). Error bars, mean±s.e.m. Two-tailed Student's t test.
(**g**) Tile-scan overview images of CD31^{+} ECs (gray) in E18.5 Wls $^{i\Delta Bmx}$ and littermate control femur. Femur length is comparable in E18.5 in Wls$^{i\Delta Bmx}$ (N=5) and littermate control embryos (N=5).
(**h**) Representative FACS plot for quantification of LSK cells and HSCs in E18.5 Wls$^{i\Delta Bmx}$ mutant and littermate control.
(**i**) Representative 3D-reconstruction of CD150^{+}, c-Kit^{+} and mature Lin^{+} CD48^{+} CD41^{+} haemato-poietic cells in E16.5 Wls$^{i\Delta Bmx}$ and littermate control femur. Quantification of normalized number of HSCs (Ctrl =8; Wls$^{i\Delta Bmx}$=8) and c-Kit+ cell area in E16.5 Wls$^{i\Delta Bmx}$ mutant and littermate control (Ctrl =7; Wls$^{i\Delta Bmx}$=6). Error bars, mean±s.e.m. Two-tailed Student's t test.
(**j**) Representative 3D-reconstruction showing CD150^{+} cells and caveolin-1^{+} arteries in E16.5 Wls$^{i\Delta Bmx}$and control femur. Quantification of the distance between CD150^{+} cells and caveolin-1^{+} arteries. Ctrl =99; Wls$^{i\Delta Bmx}$ =78, P-value, Kolmogorov-Smirnov test.
(**k**) Representative 3D-reconstruction showing CD150^{+} cells and caveolin-1^{+} arteries in E18.5 Wls$^{i\Delta Bmx}$ mutant and littermate control.

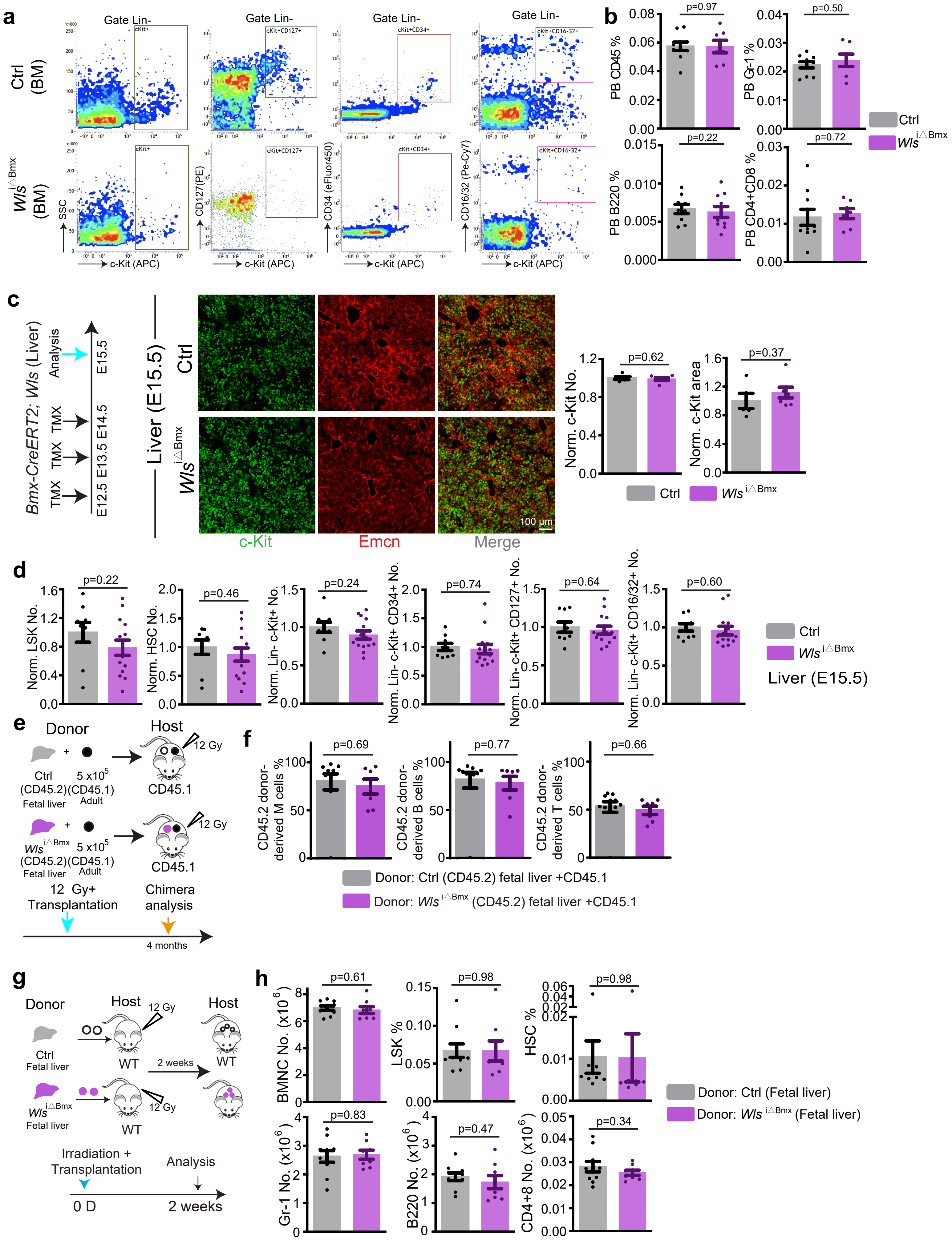
a
Gate Lin-
Ctrl (BM)
Wls iΔBmx (BM)
cKit+
cKit+CD127+
cKit+CD34+
cKit+CD16-32+
SSC
CD127(PE)
CD34 (eFluor450)
CD16/32 (Pe-Cy7)
c-Kit (APC)
b
PB CD45 %
p=0.97
PB Gr-1 %
p=0.50
PB B220 %
p=0.22
PB CD4+CD8 %
p=0.72
Ctrl
Wls iΔBmx
c
Bmx-CreERT2; Wls (Liver)
Analysis
E15.5
TMX
E14.5
E13.5
E12.5
Liver (E15.5)
Ctrl
Wls iΔBmx
100 μm
c-Kit
Emcn
Merge
Norm. c-Kit No.
p=0.62
Norm. c-Kit area
p=0.37
Ctrl
Wls iΔBmx
d
Norm. LSK No.
p=0.22
Norm. HSC No.
p=0.46
Norm. Lin- c-Kit+ No.
p=0.24
Norm. Lin- c-Kit+ CD34+ No.
p=0.74
Norm. Lin- c-Kit+ CD127+ No.
p=0.64
Norm. Lin- c-Kit+ CD16/32+ No.
p=0.60
Ctrl
Wls iΔBmx
Liver (E15.5)
e
Donor
Host
Ctrl (CD45.2) Fetal liver
5 x10^5 (CD45.1) Adult
12 Gy
CD45.1
Wls iΔBmx (CD45.2) Fetal liver
5 x10^5 (CD45.1) Adult
12 Gy
CD45.1
12 Gy+ Transplantation
Chimera analysis
4 months
f
CD45.2 donor-derived M cells %
p=0.69
CD45.2 donor-derived B cells %
p=0.77
CD45.2 donor-derived T cells %
p=0.66
Donor: Ctrl (CD45.2) fetal liver +CD45.1
Donor: Wls iΔBmx (CD45.2) fetal liver +CD45.1
g
Donor
Host
12 Gy
Host
Ctrl Fetal liver
WT
2 weeks
WT
Wls iΔBmx Fetal liver
12 Gy
WT
Irradiation + Transplantation
Analysis
0 D
2 weeks
h
BMNC No. (x10^6)
p=0.61
LSK %
p=0.98
HSC %
p=0.98
Gr-1 No. (x10^6)
p=0.83
B220 No. (x10^6)
p=0.47
CD4+8 No. (x10^6)
p=0.34
Donor: Ctrl (Fetal liver)
Donor: Wls iΔBmx (Fetal liver)

Supplementary Fig. 8 | Analysis of Wls$^{i\Delta Bmx}$ mutant liver.

(**a**) Representative FACS plots for quantification of HSPC subsets in E18.5 Wls$^{i\Delta Bmx}$ and littermate control.
(**b**) Quantification of percentage of $CD45^+$, Gr-1^+, $B220^+$ and $CD4^+/8^+$ cells in E18.5 Wls $^{i\Delta Bmx}$ and littermate control peripheral blood. Ctrl =10; Wls$^{i\Delta Bmx}$ =7. Error bars, mean±s.e.m.Two-tailed Student's t test.
(**c**) Diagram showing tamoxifen administration and analysis of Wls$^{i\Delta Bmx}$ livers. High magnification images showing c-Kit^+ hematopoietic cells in Wls$^{i\Delta Bmx}$ and littermate control liver at E15.5. Quantification of c-Kit^+ cell number and c-Kit^+ covered area. Wls $^{i\Delta Bmx}$ =7; control=5. Error bars, mean±s.e.m.Two-tailed Student's t test.
(**d**) FACS based quantification of HSPC number in Wls$^{i\Delta Bmx}$ (N=14) and littermate control liver (N=9). Error bars, mean±s.e.m. Two-tailed Student's t test.
(**e**) Diagram depicting CD45.2/CD45.1 competitive repopulation assay using Wls$^{i\Delta Bmx}$ and littermate control fetal liver.
(**f**) Quantification of competitive repopulation assay showing percentages of liver donor-derived (CD45.2, control=1 and Wls$^{i\Delta Bmx}$ =7) myeloid cells ($CD11b^+$), B cells ($B220^+$), and T cells ($CD4^+$ or $CD8^+$). Error bars, mean±s.e.m. Two-tailed Student's t test.
(**g**) Diagram depicting transplantation of Wls$^{i\Delta Bmx}$ and littermate control fetal liver donor cells into lethally-irradiated mice and analysis at 2 weeks after transplantation.
(**h**) FACS based quantification of LSK%, SLAM-LSK% as well as the number of BMNCs, Gr-1^+ myeloid cells, $B220^+$ B-lymphocytes and $CD4/8^+$ T-lymphocytes at 2 weeks after transplantation of Wls$^{i\Delta Bmx}$ and littermate control fetal liver cells into irradiated recipients. Wls$^{i\Delta Bmx}$ =8; ctrl=10. Error bars, mean±s.e.m. Two-tailed Student's t test.

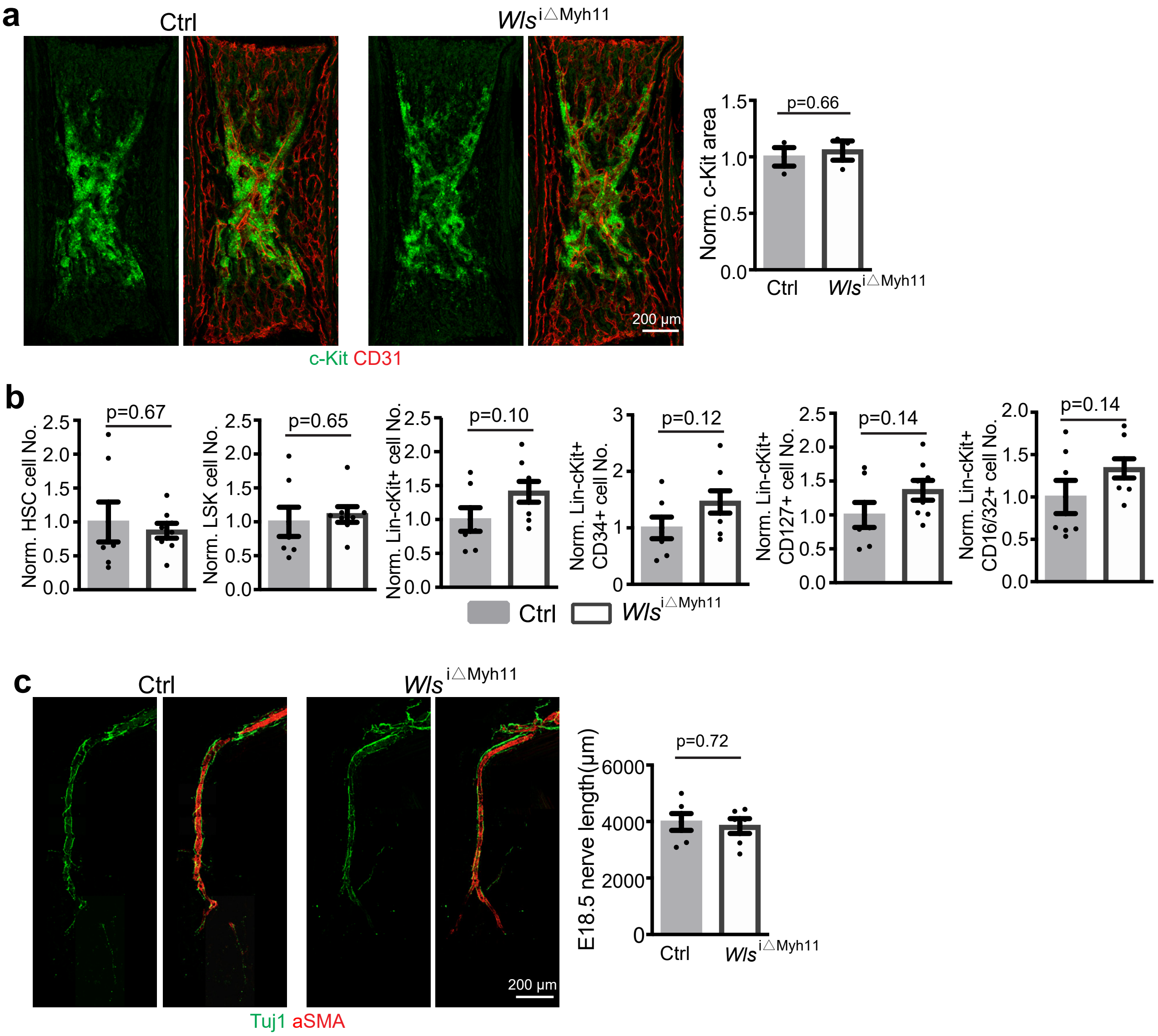
a
Ctrl
Wls i△Myh11
Norm. c-Kit area
p=0.66
1.5
1.0
0.5
0.0
Ctrl
Wls i△Myh11
200 μm
c-Kit CD31
b
Norm. HSC cell No.
p=0.67
Norm. LSK cell No.
p=0.65
Norm. Lin-cKit+ cell No.
p=0.10
Norm. Lin-cKit+ CD34+ cell No.
p=0.12
Norm. Lin-cKit+ CD127+ cell No.
p=0.14
Norm. Lin-cKit+ CD16/32+ cell No.
p=0.14
Ctrl
Wls i△Myh11
c
Ctrl
Wls i△Myh11
200 μm
Tuj1 aSMA
E18.5 nerve length(μm)
p=0.72
6000
4000
2000
0
Ctrl
Wls i△Myh11

Supplementary Fig. 9 | Normal hematopoietic cell expansion and nerve extension in Wls$^{i\Delta Myh11}$ mutants

(**a**) Representative overview image of c-Kit$^+$ cells in E18.5 Wls $^{i\Delta Myh11}$ mutant and littermate control. Quantification of c-Kit$^+$ cell covered area. Ctrl=3; Wls $^{i\Delta Myh11}$ =3. Tamoxifen was injected from E14.5 to E16.5. Error bars, mean±s.e.m. Two-tailed Student's t test.
(**b**) FACS-based quantification of normalised HSC, LSK, Lin$^-$ c-Kit$^+$, Lin$^-$ c-Kit$^+$ CD127$^+$, Lin$^-$ c-Kit$^+$ CD34$^+$, Lin$^-$ c-Kit$^+$ CD16/32$^+$ cell number in E18.5 Wls $^{i\Delta Myh11}$ knockout (N=8) and littermate control (N=7). Error bars, mean±s.e.m. Two-tailed Student's t test.
(**c**) Stitched images from serial sections of Tuj1$^+$ nerve fibres and αSMA$^+$ vSMCs in E18.5 Wls$^{i\Delta Myh11}$ mutant and littermate control. Quantification of nerve length. Wls$^{i\Delta Myh11}$ (N=4) and control (N=4). Error bars, mean±s.e.m. Two-tailed Student's t test.

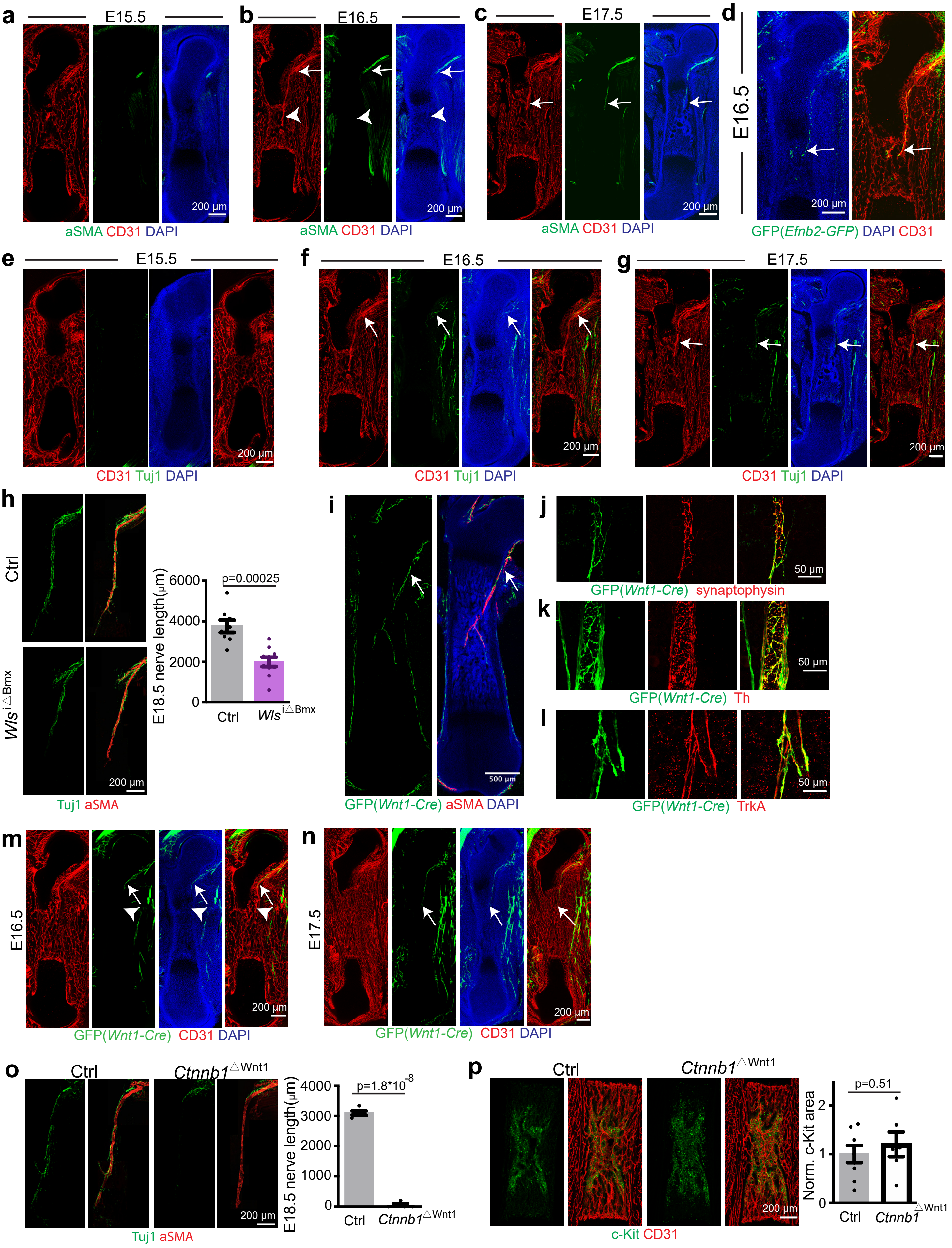

a
E15.5
aSMA CD31 DAPI
b
E16.5
aSMA CD31 DAPI
c
E17.5
aSMA CD31 DAPI
d
E16.5
GFP(Efnb2-GFP) DAPI CD31
e
E15.5
CD31 Tuj1 DAPI
f
E16.5
CD31 Tuj1 DAPI
g
E17.5
CD31 Tuj1 DAPI
h
Ctrl
Wls i△Bmx
Tuj1 aSMA
E18.5 nerve length(μm)
p=0.00025
i
GFP(Wnt1-Cre) aSMA DAPI
500 μm
j
GFP(Wnt1-Cre) synaptophysin
k
GFP(Wnt1-Cre) Th
l
GFP(Wnt1-Cre) TrkA
50 μm
m
E16.5
GFP(Wnt1-Cre) CD31 DAPI
n
E17.5
GFP(Wnt1-Cre) CD31 DAPI
200 μm
o
Ctrl
Ctnnb1△Wnt1
Tuj1 aSMA
E18.5 nerve length(μm)
p=1.8*10-8
p
Ctrl
Ctnnb1△Wnt1
c-Kit CD31
Norm. c-Kit area
p=0.51

Supplementary Fig. 10 | Femoral nerves are not required for fetal HSPC development.

(**a-c**) Representative overview images showing ingrowth of the trochanter artery into BM. Arrows mark expansion of vSMC-covered rear part of trochanter artery, arrowhead indicates arteriolar front part.
(**d**) Overview image of trochanter artery in Efnb2-H2B-GFP (*Efnb2*^{GFP}) knock-in reporter. Green, H2B-GFP; Red, CD31; Blue, DAPI. Arrow indicates trochanter artery in E16.5 femur.
(**e-g**) Representative overview images showing development of Tuj1^{+} nerves in femur at E15.5 (e), E16.5 (f) and E17.5 (g). Arrows indicate extension of nerves.
(**h**) Stitched images from serial sections of Tuj1^{+} nerve fibres and αSMA^{+} vSMCs in Wls $^{i\Delta Bmx}$ mutants and littermate controls at E18.5. Quantification of nerve length. Ctrl=8; Wls$^{i\Delta Bmx}$ =10.Error bars, mean±s.e.m. Two-tailed Student's t test.
(**i**) Tile-scan image showing ingrowth of Wnt1-mTmG-labelled nerve at lesser trochanter and extension into BM along artery (arrow) at P0.
(**j-l**) Validation of Wnt1-mTmG GFP signal in postnatal nerves. GFP signal overlaps with synaptophysin (j), tyrosine hydroxylase (Th) (k) and Tropomyosin receptor kinase A (TrkA) (l) immunostaining.
(**m-n**) Overview images showing developmental change of Wnt1-Cre-driven GFP expression in femoral BM and around trochanter artery at E16.5 (m) and E17.5 (n). Arrowheads point to the trochanter artery, arrows mark the adjacent nerve.
(**o**) Stitched serial sections of Tuj1^{+} nerve fibres in E18.5 Ctnnb1$^{\Delta Wnt1}$ mutant and littermate control. Quantification of nerve length. Ctrl=5; Ctnnb1$^{\Delta Wnt1}$ =5. Error bars, mean±s.e.m. Two-tailed Student's t test.
(**p**) Representative overview image of c-Kit^{+} cells in E18.5 Ctnnb1$^{\Delta Wnt1}$ and littermate control BM. Quantification of c-Kit^{+} cell covered area. Ctrl=8; Ctnnb1 $^{\Delta Wnt1}$ =6. Error bars, mean±s.e.m. Two-tailed Student's t test.

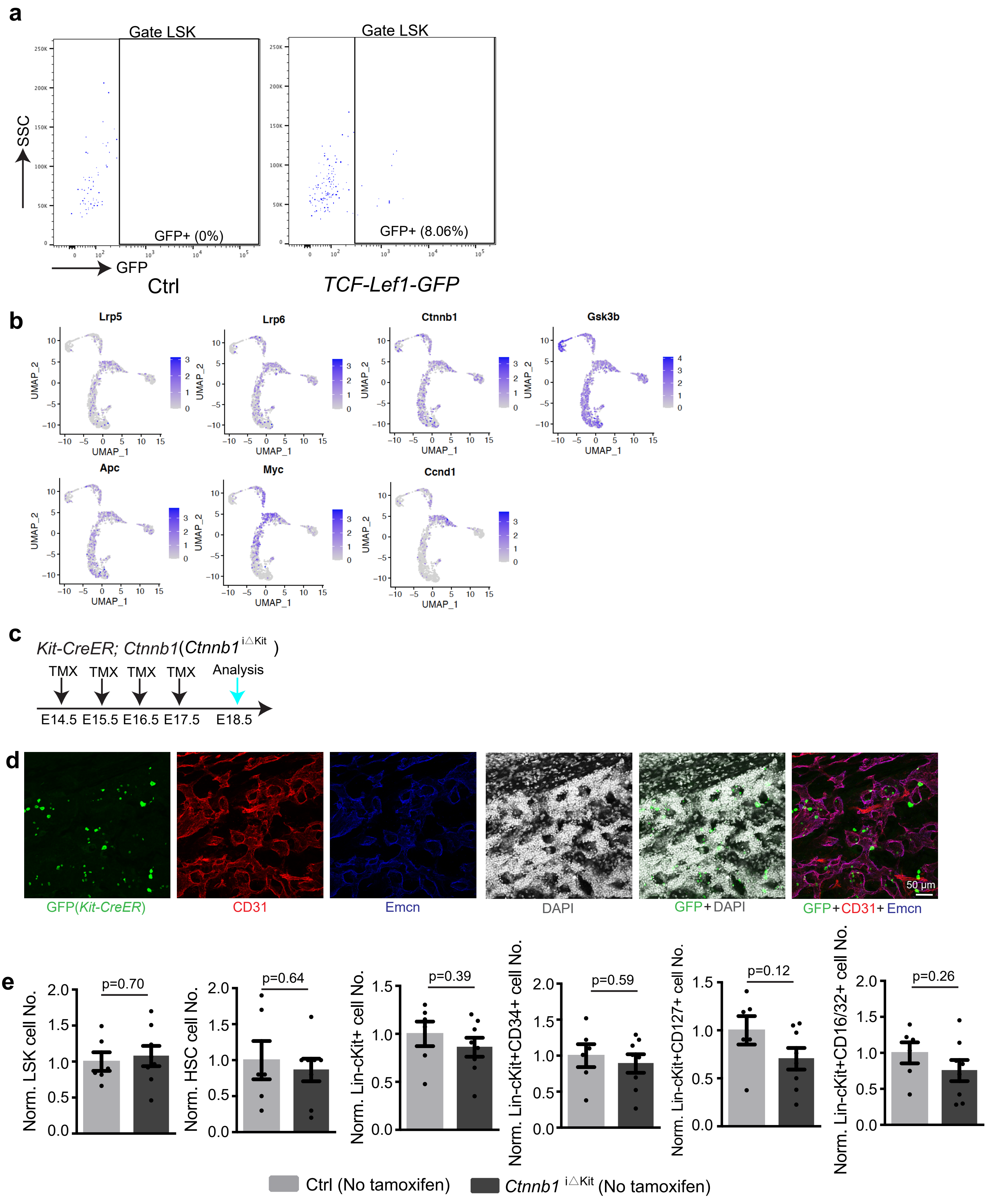
a
Gate LSK
Gate LSK
SSC
GFP+ (0%)
GFP+ (8.06%)
GFP
Ctrl
TCF-Lef1-GFP
b
Lrp5
Lrp6
Ctnnb1
Gsk3b
Apc
Myc
Ccnd1
UMAP_1
UMAP_2
c
Kit-CreER; Ctnnb1(Ctnnb1 i△Kit)
TMX
TMX
TMX
TMX
Analysis
E14.5 E15.5 E16.5 E17.5 E18.5
d
GFP(Kit-CreER)
CD31
Emcn
DAPI
GFP+DAPI
GFP+CD31+Emcn
50 μm
e
Norm. LSK cell No.
p=0.70
Norm. HSC cell No.
p=0.64
Norm. Lin-cKit+ cell No.
p=0.39
Norm. Lin-cKit+CD34+ cell No.
p=0.59
Norm. Lin-cKit+CD127+ cell No.
p=0.12
Norm. Lin-cKit+CD16/32+ cell No.
p=0.26
Ctrl (No tamoxifen)
Ctnnb1 i△Kit (No tamoxifen)

Supplementary Fig. 11 | Additional analysis of Ctnnb1 inactivation in hematopoietic cells.

(a) Representative FACS plots of LSK cells from TCF-Lef1-H2B-GFP reporter mice and littermate controls at E18.5.
(b) UMAP feature plots showing expression of canonical Wnt signalling components in multipotent HSPCs. Color represents relative gene expression level.
(c) Schematic diagram showing tamoxifen injection and analysis of Ctnnb1$^{i\Delta Kit}$ mutants.
(d) High magnification images showing very limited Kit-CreER-controlled recombination in ECs of fetal Kit-CreER R26-mTmG femur. Green, GFP; Red, CD31; Blue, Emcn.
(e) FACS-based quantification of normalized HSC, LSK, Lin$^-$ c-Kit$^+$, Lin$^-$ c-Kit$^+$ CD127$^+$, Lin$^-$ c-Kit$^+$ CD34$^+$, Lin$^-$ c-Kit$^+$ CD16/32$^+$ cell number in E18.5 Ctnnb1$^{i\Delta Kit}$ mutants and littermate controls in absence of tamoxifen injection. Ctrl=6; Ctnnb1$^{i\Delta Kit}$ =8. Error bars, mean±s.e.m. Two-tailed Student's t test.

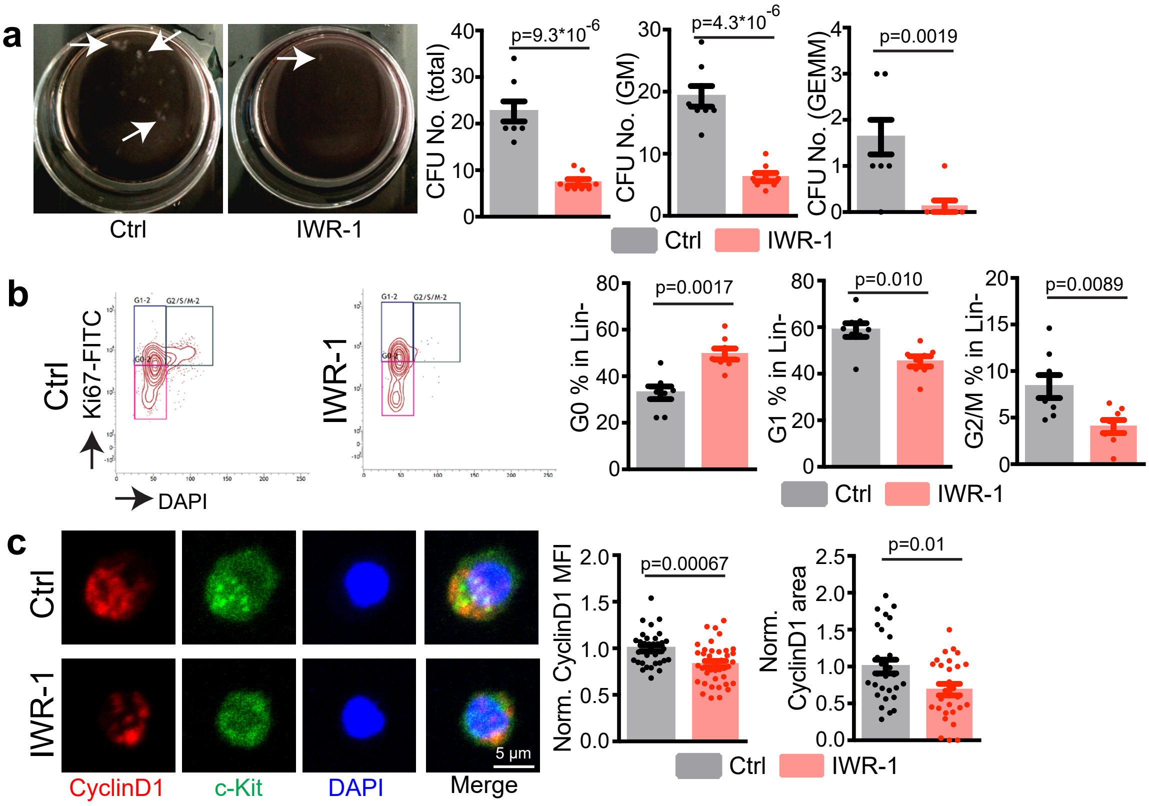
a
Ctrl
IWR-1
p=9.3*10^-6
CFU No. (total)
p=4.3*10^-6
CFU No. (GM)
p=0.0019
CFU No. (GEMM)
Ctrl
IWR-1
b
Ctrl
Ki67-FITC
DAPI
IWR-1
G0 % in Lin-
p=0.0017
G1 % in Lin-
p=0.010
G2/M % in Lin-
p=0.0089
Ctrl
IWR-1
c
Ctrl
IWR-1
CyclinD1
c-Kit
DAPI
Merge
5 μm
Norm. CyclinD1 MFI
p=0.00067
Norm. CyclinD1 area
p=0.01
Ctrl
IWR-1

Supplementary Fig. 12 | Effect of IWR-1 on HSPCs

(a) Representative images (dish at day 8 after seeding) and quantification of colony forming units from 1000 Lin- c-Kit^{+} cells isolated from neonatal wild-typed mice. IWR-1 or vehicle was added to MethoCult medium (vehicle=8; IWR-1=8). Error bars, mean±s.e.m. Two-tailed Student's t test.
(b) Representative FACS plot showing Lin- cells in the G0, G1, G2/M/S phase of cell cycle (left) and quantification of Lin^{-} cell percentage in each stage of cell cycle (right) after IWR-1 treatment. Ctrl=8; IWR-1=8. Error bars, mean±s.e.m. Two-tailed Student's t test.
(c) Representative image of CyclinD1 expression in FACS-sorted Lin^{-} c-Kit^{+} cells after IWR-1 treatment. Red, CyclinD1; Green, c-Kit; Blue, DAPI. Immunostaining based quantification of mean fluorescent intensity (MFI) (Ctrl=32 cells; IWR-1=40 cells) and area of Cyclin D1+ signal (Ctrl=28 cells; IWR-1=28 cells). Error bars, mean±s.e.m. Two-tailed Student's t test.

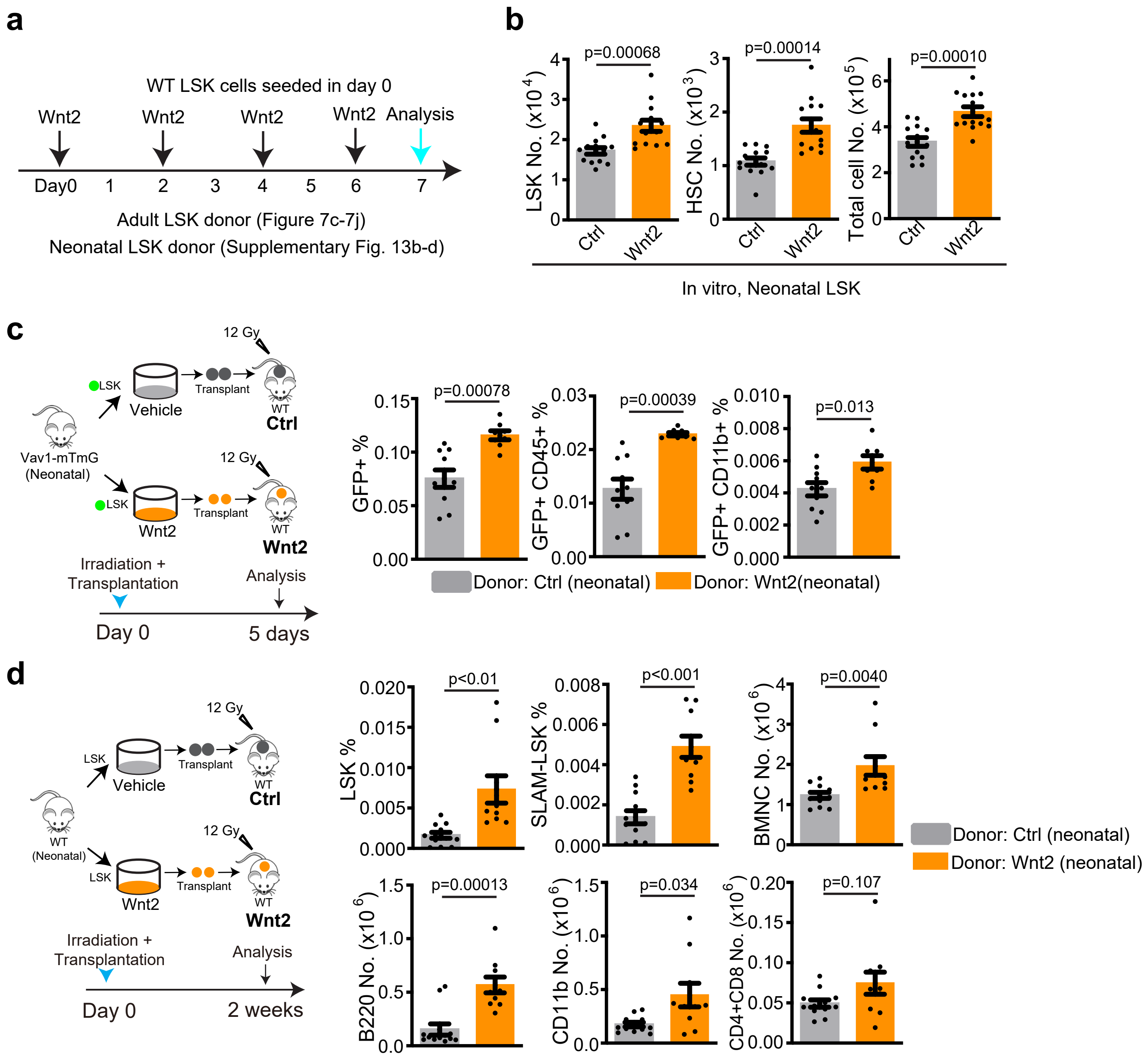

a
WT LSK cells seeded in day 0
Wnt2
Wnt2
Wnt2
Wnt2
Analysis
Day0 1 2 3 4 5 6 7
Adult LSK donor (Figure 7c-7j)
Neonatal LSK donor (Supplementary Fig. 13b-d)
b
p=0.00068
p=0.00014
p=0.00010
LSK No. (x10^4)
HSC No. (x10^3)
Total cell No. (x10^5)
Ctrl
Wnt2
In vitro, Neonatal LSK
c
12 Gy
LSK
Vehicle
Transplant
WT
Ctrl
Vav1-mTmG
(Neonatal)
Wnt2
Irradiation +
Transplantation
Analysis
Day 0
5 days
p=0.00078
p=0.00039
p=0.013
GFP+ %
GFP+ CD45+ %
GFP+ CD11b+ %
Donor: Ctrl (neonatal)
Donor: Wnt2(neonatal)
d
WT
(Neonatal)
2 weeks
p<0.01
p<0.001
p=0.0040
LSK %
SLAM-LSK %
BMNC No. (x10^6)
p=0.00013
p=0.034
p=0.107
B220 No. (x10^6)
CD11b No. (x10^6)
CD4+CD8 No. (x10^6)
Donor: Ctrl (neonatal)
Donor: Wnt2 (neonatal)

Supplementary Fig. 13 | Additional data about the effect of Wnt on HSPCs.

(a) Diagram showing protocol for Wnt2 treatment in vitro.
(b) Quantification of HSC number, LSK number and total cell number after Wnt2 treatment. The same number of neonatal LSK cells was seeded. Vehicle=10; Wnt2=9. Error bars, mean±s.e.m.Two-tailed Student's t test.
(c) Diagram showing Wnt2-treatment of sorted Vav1-mTmG neonatal LSK cells in vitro followed by analysis at 5 days after transplantation into lethally-irradiated WT mice. FACS based quantification of GFP^+, GFP^+ $CD45^+$ and GFP^+ $CD11b^+$ cell percentage in BM at 5 days after transplantation. The same number of neonatal LSK cells was seeded before Wnt2-treatment. ctrl=10; Wnt2=8. Error bars, mean±s.e.m. Two-tailed Student's t test.
(d) Diagram showing Wnt2-treatment of sorted WT neonatal LSK cells in vitro followed by analysis at 2 weeks after transplantation into lethally-irradiated WT mice. FACS based quantification of LSK%, SLAM-LSK% as well as the number of BMNC, $Gr\text{-}1^+$ myeloid cell, $B220^+$ B-lymphocyte and $CD4/8^+$ T-lymphocyte. The same number of neonatal LSK was seeded before Wnt2-treatment. ctrl=12; Wnt2=10. Error bars, mean ±s.e.m. Two-tailed Student's t test.
