# Supplementary material for: A specialized bone marrow microenvironment for fetal haematopoiesis
Source: Nat Commun. 2022 Mar 14;13:1327. doi: 10.1038/s41467-022-28775-x (PMC8921288; doi:10.1038/s41467-022-28775-x)
Supplement: Supplementary file 3 — Description of Additional Supplementary Files [file 41467_2022_28775_MOESM3_ESM.pdf]

Description of Additional Supplementary Files

File Name: Supplementary Data 1
Description: Metadata tables of cell-to-cluster assignment

File Name: Supplementary Data 2
Description: Tables of cluster-specific markers

File Name: Supplementary Data 3
Description: Metrics summary of single cell RNA-seq experiments

File Name: Supplementary Data 4
Description: Amplicons for targeted scRNA-seq in FASTA format
