# Supplementary material for: A specialized bone marrow microenvironment for fetal haematopoiesis
Source: Nat Commun. 2022 Mar 14;13:1327. doi: 10.1038/s41467-022-28775-x (PMC8921288; doi:10.1038/s41467-022-28775-x)
Supplement: Supplementary file 7 — Supplementary Data 4 [file 41467_2022_28775_MOESM7_ESM.pdf]

>Acan|ENSMUST00000032835.6|PolyA_1 location: chr7(+): 79114408 site_id: 441410, AMPLICON
ATGCTAGAACCCTCGGGCAGAAGAAAGATCGCTACGAGATCAGCTCCCTGGTGCGGTACC
AGTGCACTGAGGGCTTTGTCCAGCGCCACGTGCCCACCATCCGGTGCCAGCCCAGCGGGC
ACTGGGAAGAGCCTCGAATCACCTGCACAGACCCCAACACCTACAAGCACAGGCTACAGA
AGCGGAGCATGAGACCCACACGGAGGAGCCGCCCCAGCATGGCCCACTGAGAGGAGCTTC
CATAATGTGCCCAGGATGCTGAGCCCAGCGGCCAGCCAGGCTGACCGTGCATCCCACCCA
CATGGTGTCTTCTTGTCGCTTTTTGTCATATAAGGAATCCATTAAAGAAGGAAAAAAAAC
CC
>Acp5|ENSMUST00000069330.13|Reference_end location: chr9(-): 22126732 site_id: 1310963, AMPLICON
AATCACTCTTTAAGACCAGCCTCCCAAGGAGACCCAGACCCTGAACACCACGAGAGTCCT
GCTTGTCCGCTAACGGGTGTGAGGGAGGAGGCGTCTGCAGGCGTGGTGGGTGGGCCCTGT
GAGGACCTCACTCACGGCAGCTTCTCTGCCCTGGTACTCTGGCAGAGAGGGAAGGGGAAC
CAAGGGGCTTACTTACAGGAAAGAAGTATGCCCACACCAACTGATCCCACAGCCAGGCTC
ACCCCTTTTAGGGTAGAGCTTTCTTGGGAACGGGCTTTCCTTTCAGTCAAGTCTTACTGT
CACTGCTATTCAATAAAACAATGCTTTGTCTCC
>Acta2|ENSMUST00000039631.9|PolyA_1 location: chr19(-): 34241525 site_id: 1448698, AMPLICON
ACCGCAAATGCTTCTAAGTCCCCCCTGCTCTGCCTCTAGCACACAACTGTGAACGTTTTG
TGGATCAGCGCCTCCAGTTCCTTTCCAAATCATTCCTGCCCAAAGCTTTGATTTGTTACT
CGTGGTTTTTTTAAAAATAAATCAGACATGTGCTACCCT
>Adipoq|ENSMUST00000023593.5|PolyA_2 location: chr16(+): 23157739 site_id: 478107, AMPLICON
ACCAACTGACTGCAACTACCCATAGCCCATACACCAGGAGAATCATGGAACAGTCGACAC
ACTTTCAGCTTAGTTTGAGAGATTGATTTTATTGCTTAGTTTGAGAGTCCTGAGTATTAT
CCACACGTGTACTCACTTGTTCATTAAACGACTT
>Alpl|ENSMUST00000030551.10|Reference_end location: chr4(-): 137741748 site_id: 1284765, AMPLICON
AGTCACGGCCAGTCCTCAAGCCCAACCCTCCCTGGGGGGAAGACCAGGTCTGCTCAGGAT
GAGACTCCCAGGAAGCCACCTCCGGGGTTGGCTGTCTACCCAGGGTTGCCAAGCTGGGAA
GAACACTCCAGCCGGACAGGACACACACACACACTCCCCACCCAATTGCAGAGACTCGCC
AACCCTTCACTGAAGTGGCTCTCCTGTTTGGAATAGCGGGGTGGGGTGGGGGAGaagaaa
gaaagaaagaaaaaaaATTTTTAATTTCTCTTTTTGGTGTTGGTTAAAAGGGAACACAAG
ACATTTAAATAAAACATCCCAA
>Amer1|ENSMUST00000084535.5|Reference_end location: chrX(-): 95420335 site_id: 489634, AMPLICON
GTGAGGGTTGGGTCTATTAAGAGGGGGCTTTAATTATTCCTGTTACTATTGGTGCTTATT
TTCCCCGCTGTATATGGGGGTGGGGAAGGGCTGGGGTAAACCAGAAATGTATATTTAGGT
CACGTTAAATAAAATGGCTGGGGGAAGAAGAAAGATATGAGTCTAGGTGTAAGAGTTCTC
TCTTCCAGGACAGAAAGGGGATTGTAGCTTGTTGCTGCACCTGAGCTTCCCTTCCGCATT
CCTGAGACATCATGGAATCATCTGCATTTGTTACCTCTCCATCCCTAGCACTCACTGGCA
TCTGGATATCCTGATACACTGCCTGTATGTTACAACATATATTTCATTTGCTGAATGCTA
ATAAACTTCTGTGCTCAT
>Amfr|ENSMUST00000053766.13|PolyA_1 location: chr8(-): 93971927 site_id: 449037, AMPLICON
GATCGGCTCAAATTCTCCACGTATTTAAGGATTTGAAACATGCAATCATAGGATACATAG
TTTTATGTCCCATTGGATTAAGGTTTTTTTTTTTTAATGTTTCAAAACATAGTTTTATGG
TAGTCCTTTTGGGAAAAATCCAGTATTATCTAAAATTATTGGCAAAGTTAAATGTATTTT
ACATACCTGGAAGTTTTTAGACTGTTGATAAGTAACTGAGAAAGGATAACAGGCGCCTAG
GTGAAGATAATTTATTTCAATAAATCTTTCAAAAGC
>Apc|ENSMUST00000079362.12|PolyA_1 location: chr18(+): 34319538 site_id: 1467677, AMPLICON
ATGGTGTGCAGAGAGATAGCTACAGTGTAACGATTCACACTATTTTGTGCTCAAATCTGT
GTAACTGTAAAGCATTGAATGAAACTATTTTAACTGAACTAGATTTTATCAAAAGTAGGT
AGAATTTTTGCTATGCTGTAACTGTTGTATATTCTGGTATTTGAGGTGAGTTGGCTGCTC
TTTTATTAATGAGACACGAATTGTGTCTCAAACTAAATGAACATTTCAGAATAAATTATT
GCTGTATGTAAACTATTACTGAAATTGGTATTTGTTTGAGGGTCTTATTTCACATTTGTA

TTAATAACTGTCAAAAGGGCCTCTTTTAAAAGCTAAATGTAATTTTTTTTCTTCAGATTC
TATGCATTAAGAGTAAAATTTCCTCTTGCTG
>Apc|ENSMUST00000079362.12|PolyA_2 location: chr18(+): 34319395 site_id: 1467678, AMPLICON
CATGGTGTGCAGAGAGATAGCTACAGTGTAACGATTCACACTATTTTGTGCTCAAATCTG
TGTAACTGTAAAGCATTGAATGAAACTATTTTAACTGAACTAGATTTTATCAAAAGTAGG
TAGAATTTTTGCTATGCTGTAACTGTTGTATATTCTGGTATTTGAGGTGAGTTGGCTGCT
CTTTTATTAATGAGACACGAATTGTGTCTCAAACTAAATGAACATTTCAGAATAAATTAT
TGCTGTATG
>Apc|ENSMUST00000079362.12|PolyA_3 location: chr18(+): 34322188 site_id: 1467679, AMPLICON
ACCCAGGTCATTTTCCTCCTGTTCTAACTCTGCTTTTTGTTAGGCCATTGATGCATCTTT
CCGACTGTATGTTCTGTGTATAATGCTGTTCTTGGTGCTAAATAGACTTTCTTTCCTCTG
ATATTCAGGATAATTTTTCTCTATACTTCTGTTCACTTGTTTTCTTGAAGTTATCTGATT
GTTATTTTTAATTAAAAATACTTAAATG
>Apln|ENSMUST00000039026.7|Reference_end location: chrX(-): 48025149 site_id: 488396, AMPLICON
GCTCACAAAAAGACTCGCTATGCTTAAGGGCCAGTTCACATAAATTTGAGCCCCTTTTAA
GTCCTTTGGCATCTGTTCGAATGTCCTCATTTTTCTGGCAGTGTCACTTAAAGGATTTTT
TTTCCAGAAGCAATTCATTTAAGGACACGCTGATCAAAGGTGGGATTTGTACTAAACGCT
GTTTTGTAGCCCTGGTGGGTGCCTTCTGTTTTATCTGCTTTTGAATACTTTCGGGACTTT
TTAGCCAGTTTGCCTTTCTTGACAAATGTTGTCCTCAGCCATAAATACATTTGGTAATGA
CTTTGTATGTATCGTTTTATTGTTTCACAAAGTGGAGTTGCTTGATGAATGAGATAACCT
GGAAAATAAACTTCGAGGAGTTCGATAG
>Aqp1|ENSMUST00000004774.3|Reference_end location: chr6(+): 55348523 site_id: 432542, AMPLICON
CTTAGCAGTCAAGGCCATGTTTGGCCACCCCATGGTCAATGACGTGGACTCCTCTCACCT
CCAGCTTCTCAGTTTCTGCCTGGGCAATGGCTAAGGGCCAGGGGTGAGGGTGGGGGTGGA
GGTGGTGAGAGCTTTGCTGGAGAGGGGCAGGGCAGGCCCATCCCACCTGGTTCTGGCCAT
TGTGAACCAGGACCTTGTATCTGTCTGCTCTGTGTATGTTTCTTTGCAATTGAATTTCAT
CTTATG
>Aspn|ENSMUST00000021820.13|Reference_end location: chr13(+): 49567559 site_id: 1113965, AMPLICON
GGAACAGTATGGACGCCAATCAATTTTATATCAACTTATCTCTTCAAATATGCACATTGG
GTAATGCCTGGAAACATAGCTAAGGTGACAAAAACTGAAAACTGAACAAAACTTAATAGT
ACTTTCATGTGTTTTTTTTAAACTGATATTCATTATGAATTAAGTAAAAAGTGACAATAA
GGAAAACATTAAATACTGGTTTTCA
>Atp6v0d2|ENSMUST00000029900.5|Reference_end location: chr4(-): 19876839 site_id: 418024, AMPLICON
tcaaccaccttagactgtcctctcaaaccctgacccaaaagaaacccttccctttctaaa
ctgttgtttcaggtattttgtggcagcaacacaacaaagtaactaatacagaaaactgat
actgccattgctacaataaacttgattttgggatt
>Atp6v0d2|ENSMUST00000029900.5|PolyA_1 location: chr4(-): 19878071 site_id: 418025, AMPLICON
GCTGGCATTCAACAGGCAATTCCATTATGGTGTGTTTTATGCGTATGTAAAGTTGAAGGA
GCAAGAGATGAGAAATATCGTGTGGATAGCAGAATGCATCTCACAGAGGCATCGAACTAA
AATCAACAGCTACATTCCAATTTTATAAGCCAGTGTACAGAAGATCATACATGTTGCCAT
GAAGTTATTGAGGAAAGGAAGGGGGATTGTGTCACATTATCTAGATTATATAAAAGTAAG
TCATACCACCTTTCCATAAACTACATGTCCACTGGAAGCCCAGTAAACAGAACTTG
>Axin1|ENSMUST00000074370.9|Reference_end location: chr17(+): 26195809 site_id: 480766, AMPLICON
CATGTCACTGAGTGCCTTCAACATAGCTGTCTCTTGCCTGCCACTGTGTGAATCTGGCAG
CTGAGTATCTCAGGCCCCTTTGCCTGTCTCTAGCCACCAGCTTGGTTCAGCAGGAGGGGG
GGCGGTGTGTCTGGTCCCTTCCAAGTGTCCGTGTAAATATGTACATTTCTCAGGCCAGGG
CCAGCAGGGGGATACCCTGAGCCCATTTTTCATGCAATGACTTGTACAATTATCTTTTCA
AAGGTACTTGGATAATAATGAAATAAAAACGTTTTTGAACCT
>Axin2|ENSMUST00000052915.13|Reference_end location: chr11(+): 108950778 site_id: 464753, AMPLICON

TGGCTAGTGCTTGACTTGGGAATCAGGACAGTACCTGTACAGGCACGGGGACCCGCTCCG
TCCGTGCGCCGCCCTATATTGAGGGCTCCAGCTCTCCCTTGGTTTTTGAAAGGGGTTTAT
GTATAAATATATTTTATGCCTTTTTATTACAAGTCTTGTTACTCAATGACTTTTGTCATG
GCAGTTTGTTCTACTTTAGACTGTAAATTATGCATTATAAAGAGTTCATTTAAAGAAAAC
TACTTGGTACAATAATTATTGTAATTAAGAGATGTAGCCTTTATTAAAATTTTATATTTT
>Bglap|ENSMUST00000076048.4|Reference_end location: chr3(-): 88383501
site_id: 414166, AMPLICON
TGAAGACCGCCTACAAACGCATCTATGGTATCACTATTTAGGACCTGTGCTGCCCTAAAG
CCAAACTCTGGCAGCTCGGCTTTGGCTGCTCTCCGGGACTTGATCCTCCCTGTCCTCTCT
CTCTGCCCTGCAAGTATGGATGTCACAGCAGCTCCAAAATAAAGTTCAGATGAGG
>Bglap2|ENSMUST00000098956.2|Reference_end location: chr3(-): 88377736
site_id: 414164, AMPLICON
TGAAGACCGCCTACAAACGCATCTACGGTATCACTATTTAGGACCTGTGCTGCCCTAAAG
CCAAACTCTGGCAGCTCGGCTTTGGCTGCTCTCCGGGACTTGATCCTCCCTGTCCTCTCT
CTCTGCCCTGCAAGTATGGATGTCACAGCAGCTCCAAAATAAAGTTCAGATGAGG
>Bglap3|ENSMUST00000075523.10|Reference_end location: chr3(-): 88368622
site_id: 414160, AMPLICON
CCCTGCTTGTGACGAGCTATCAAACCAGTATGGCTTAAAGACCGCCTACAGACGCATCTA
CGGTATCACTATTTAGGACCTGTGCTGCCCTAAAGCCAAACTCTGGCAGCTCGGCTTTGG
CTGCTCTCCAGAACTTGATCCTCCCTGTCCTCTCTCTCTGCCCTGCAAGTATGGATGTCA
CAGCAGCTCCAAAATAAAGTTCAGATGAGG
>Bmx|ENSMUST00000112265.8|Reference_end location: chrX(-): 164192841
site_id: 491476, AMPLICON
ggcttcaccctactatctctgcttcttgcttacaattcaagatgtgagctctcagaagct
gaaaaggtggcttggtgcttaagaacaccggctgttcttctagaggacccagactcaatt
cctagcacccacacagtggtttacaacttgtctatgaatccagttctaaggtatctgatg
acgtcttttggccttcatggacaccatggacacgtgtggtacactgacatgtatacaggc
aaaGAAGAAAAATAAaggatgagagctctcagcatcctgctctatctgcctgtttgcaag
ttgcctttgccctgccattttgaactctaactttctggaactataagccaaataaagcct
ttcttctat
>Car2|ENSMUST00000029078.8|Reference_end location: chr3(+): 14900768
site_id: 1275635, AMPLICON
AGTCCACATCATGAGACAAACTGAAGTAACTTAGGCAAAACAGGTAAAACAGTCATAGTT
TTGTGATTATAAATGAGATGAATGTTCACCCTTCCAAGATCTTATATTAAAGAAAAAATT
TTAAAAAGCTTATATATTTGTAGCAAAGTTATTCTTAAATATGAATTATGTTATAACTTA
GTGACTTTTGATTTCTAGAGGTGTAAATGAGGATGTAAAAATTGATATAGTTGTGATACA
GAGTATATTTCCCTTCAGATAACATACCACAACACAATGGATAATGTATTTTAGATATAT
TCTCTAATAAAATTGAGAACT
>Car3|ENSMUST00000029076.5|Reference_end location: chr3(+): 14872509
site_id: 1275633, AMPLICON
CCAGCGATAATCCATCACTCGTTAAAAATTTGCCTACTACCAAGTTTGCCTGGTTTTAGT
CACCTATAAAACACACCCATGAAGCCAGGTGTTTTAAAATGTTTGATCCAGCATTTAAAT
TTCTTCTTCATAAAGATGGTTTTCTTTGCCCAAAGTAGAGCCATTTATTTTTTATTTCAC
TACTTTAATCTTTGCATGCCTATTaaaaacaaaaacaaacaaacaaaaaaaaaaacaaaaa
cagcaacaacaaaaaaaacTGTGTCAGGTGTCTTTTGAGTTGGAGGCTGGGATGTCTGGT
TCCTGGAACAGGAAGGAGGGAATGTCAACACTGAAGCCAAGAGCTTGCATTGATCACAGA
A
>Cav1|ENSMUST00000007799.12|Reference_end location: chr6(+): 17341297
site_id: 430968, AMPLICON
GAACCCTGCTTGACTTGTTTTCTCCTTCAGTCTTGCCGACACTTTACCAACCTGCTACCT
ACTTTGATTGTTTGCATTTAAAACAGACACTGGCATGGCCACAGTTTGAATTTTAAACTG
TGCACATAACTGAAAGTGTACTAGACTGTATACCTTTTTACATGTAGAGATATTCTTTAT
CTTTATATAAGGAGAATCACTTGGGAAATGATTCTACAATTCAGTCTGTAAACTGTGTGT
TCCAAGACATGTCTGTTCTCCCTAGATACTCAGTTTTATACAAGTCAATTGCTGATCCAA
AAGGTTACTGAAATTTTATATGCTTACTGATATATTTTACACTTTTTTATGCTGCATGTC
CTATAAAGATTTCAAA
>Ccar2|ENSMUST00000035612.6|Reference_end location: chr14(-): 70138177
site_id: 1331295, AMPLICON

TTAGGTGATGGAGCCCATTCAAGGAGCATTTGGTGTTGGGTCAGGGTTGCCAATAGAACA
CTGTGTCCCAGCTGCCCCCAACTTGCCAGAAGAACCAGCACTTACTTCTTTCTCTAGTTC
TTATGTTCAGAATGTGGTATTGGCCCTGGCCTAGCCCCTTCCACTTCCCATAATTCTTGC
CTCTTTCCATAATTCATGGTTTCAGTTCGACTTTGTATATAAAGttgtttgttggttttt
ttttttttttttggctttttgttttttAAATAAACCAAAGTGA
>Ccar2|ENSMUST00000035612.6|PolyA_1 location: chr14(-): 70138684 site_id: 1331296, AMPLICON
CACCTTGAGATGCAGCGGATTGTTGAGAAGGCTGACAGCTGGGTAGAGAAAGAAGAGCCA
ACGCCTAGCAACTGAGTGGCTGCCTAGTGAGGTCTGCGATGGAGCCTGATGACATTAGAG
CCCTTTTGGTACCAGAAGGCAGCTGGAGCAGGGCCTGGGAGCCACAGCAGGGGTAGCTTG
AGTTCTGTGCTCAGCCTCTGCAGGGGATGTCAAACTCAGTATGGGGTTTGTGCTATGTGG
ATGGATATGTAAGGAACTCCAGTTCCAGCTACAACTAAATACAACGTCCTTTGC
>Ccn3|ENSMUST00000050027.8|PolyA_1 location: chr15(+): 54753533 site_id: 475120, AMPLICON
ATAGACAAACCGACTCCTTTCCAATAGAAAGAAGCTGAACCAAAAACTCCCAATGCAGAG
ATGACGGGGCCTCTCAGCTCATGGTTTGTACCCCGGAGGCTGCTGAGGCCCACTTCTTCA
AAATTAAACAGGGACTCTTTCATAGTTAAAGTACACTGAAGACCAAGCCACCTAATTTAC
AATCTCTGATGATTTTCAAATGTGCTTTTAGTGCAATTTTGAAAAGGAAGAGCAGAATCT
ACAAAATTTTATGAAAGTTTAAAGTAGCTGAAGACCTGCTCAAAATACATCTTTTGGTCA
TAGTTATGAAAACACAAGTTATACATGTGTACCTATGTATGCTTAATAAACAAAGTTCA
>Ccna2|ENSMUST00000029270.9|Reference_end location: chr3(-): 36564873 site_id: 412698, AMPLICON
CTCTTTCCGAATCTTACCCTGTTCTAAGTAAGTAGCTCCTTGCAGCTCTGAAAATTTGTA
AAAATTGATGTTTTCTATAAACTCTTTCTTTCCTTTTTCCCCAGAGGACATGTAGGCCAG
AATGCTCTAATTCTTGCTACCAGGACTGCCCATGAAAAATGTAGAAAACTGCTTTGTTTG
CCCATTTTTCTCTTGAACAGTAATTATATATGTGTGTAAATAAAGCATGCAAATTAA
>Ccnd1|ENSMUST00000093962.4|Reference_end location: chr7(-): 144929979 site_id: 1305822, AMPLICON
TCTGTTAGGTTCTAGTGTTCCGTCTTGTTTTGTGTTAATTACAGCATTGTGCTAATGTAA
AGACTCTGCCTTTGCGAAGCCAGCTGCAGTGCTGTAGGCCCCCAAGTTCCCTAGCAAGCT
GCCAAACCAAAACGGGCACCACCAGCTCAGCTGAGGCATCCCAGCCAGGCAGGACCCTTG
AGGGCCGCTGTATCCATGGTGATGGGGTGAGGTTTTGGCCAAAAGGCCAAAGACTGGTGG
TGGGTCCACGGAATCTGCCCTGTGACATGAAAGGCTTTGAGGGGCTCTGGCTGGTGGCCA
GGTTGGCTTTTTGTATTTCTGGTTGACACACCATGGCGCTTCCCAGCACAGACATGTGAC
CAGCATGGTCCAGGA
>Ccne1|ENSMUST00000108023.9|Reference_end location: chr7(-): 38097987 site_id: 439475, AMPLICON
TACTTGACCCACTGGACTCTTCACACAGATGACACAAGCTTGAAGTTAAGGAGGCCACGC
CATGGCGGTTGCTGGCCTCTGCTCGGGTGTTGTAGGTTGCTGTTGATAGGCTGTGACCAG
TGGTTGTGAGCAAGTGTGGGATGCAGAGCCCACAGCCTGGCCTAGGCTGCCCTTCTCCAC
TGTCAGCCAACAGCATGTCCTGCCTTCATGAACTCTTATTTTGTAAGTGCTGCTATGTCT
ATCAATTTTTAATAAAGATAATACTATCTTTG
>Ccny|ENSMUST00000053917.5|PolyA_1 location: chr18(-): 9315947 site_id: 1327257, AMPLICON
CAAATCGGGAATGCCATGCTATGCCCTTGGAATCTGTGTGCTCTGTAAGAAAGACTTCCT
GCATTCCTTCATGCTGCTCTCGGGATTGGGGATGGGGATGTTTGTTCATTTTTATTCTGG
TTGGGTTTTGATTCCTCAGAGCAGAGAGTATAGTTTGTAACCACCATGGCACAGACATCC
AAATAAATAGTACTGATTGTT
>Cdc73|ENSMUST00000018337.8|PolyA_1 location: chr1(-): 143604102 site_id: 1265982, AMPLICON
GGGATTGTGTGTTAGGAACTGTTCATAAGAGTATCTTGGAAGTTACAAATTGCCATATAC
TGAAATACAGTAAGCATTCTATATGAATAAAGTTTGTGGACTACAACTAGTTTTGTGCCA
AAATTAAAAAGATGGGATTATATGTCAAAATTAAGGAAACTTTCCAAGTATTTTGTATTC
TGTTAAATTTTGTTTCTATTTTAAATGTAAGTAAAGCTACTTAGCCTC
>Cdc73|ENSMUST00000018337.8|PolyA_4 location: chr1(-): 143607377 site_id: 1265985, AMPLICON
TGGGAAGCTACAGTATGATTTGGGGGGATGAGTGTGTGTCAACATAGTTACAGTGTAACT
TGAACCTCCTCTAAGTCTGACTATGATTCTTATTTTATATGCGATAAGATTTTGAGTCTT

CTATTTGTACACATGTCAACTTGGGACTTCTTATCTTCCATTATCCCTAAATAATGATAA
ATTCCCAGGCACCAAAGAACACATTTGCTTACGTGTCTGAAAATGAAACAAGATAAAAAC
ACTGGTATTTTTATGTGTGTGTGTTCAATGTGGTATAAAATATAAAACCTATATTTTAAC
GTAGTAAGATATTTTACTATTCCTCTACTTTACAGAGTGTTGCATCCACGCTACAATTAA
TGACCATATTCCTTG
>Cdh13|ENSMUST00000117160.1|PolyA_1 location: chr8(+): 119324449 site_id: 450047, AMPLICON
AGGCTAAGGGCTGAACTATTCTCCCATGGGCAAGTCTGCCACTGTACCACCCCAGACCCA
CAGTCCTATGGTCCTTGTGGTGACACAGATCATGGTGGTGGCCCTCTCCCTCCTCGGTGT
ATGTACAAGTACAAATGTCATCATGGTGTACCTATGAGGAGACCAACAGCACCATCTTGT
ATGCACATCTATTACCCACACATGTACACACGCATTACGCATATCCGCACCGTTTGTTTC
CTATATACAGGCATAAAATAGAGTAAGCCCAGGTAGTTTTTCAAGTACCCTTCCGTGTGA
CTACCGTTGTTCGCAAAGCTGA
>Cdk1|ENSMUST00000020099.12|PolyA_1 location: chr10(-): 69338306 site_id: 1318079, AMPLICON
TCGTGTTGTTTACCGTTGGCTCTCTTCCTGTCTTGTATAGTTTTCTTTGTTTGTAAACTG
TCATCTGGACTTTTCTTAATTTCCTACGTATAACTTAATTAACATGTAAATATTATTCCA
TATGAATTTAAATATAATTCTGTATATGTG
>Cdk14|ENSMUST00000030763.12|Reference_end location: chr5(-): 4803393 site_id: 423789, AMPLICON
CCCCACTGGTTTATATAACGCACTGAGATGTGTACTTTTTATTGGGGTTAGTACCTCAGA
GACTCATTATGCTTCAACATGACACATAGCAAGGAGAATGGAATACTACATGTTGCATAT
GTGTTGCCAATTGTAATTCGTCTGTATTATGAAGGATGTAATGGTTTGTCAGCTGTCATT
GTTTTCTGTAACATGATATGGAATAAAATATAGCCAAATCTGC
>Cfh|ENSMUST00000111976.8|Reference_end location: chr1(-): 140085878 site_id: 1265961, AMPLICON
GCATTGAAGTATTGTTTAACTCATGTCTTCTCATAAATATAAACATTTTTGTTATATGGT
GATTAATTTGTAACTTTAAAAACTATTGCCAAAATGCAAAAGCAGTAATTCAAAACTCCT
AATCTAAAATATGATATGTCCAAGGACAAACTATTTCAATCAAGAAAGTAGATGTAAGTT
CTTCAACATCTGTTTCTATTCAGAACTTTCTCAGATTTTCCTGGATACCTTTTGATGTAA
GGTCCTGATTTACAGTGGATAAAGGATATATTGACTGATTCTTCAAATTAATATGATTTC
CCAAAGCATGTAACAACCAAACTATCATATATTATATGACTAATGCATACAATTAATTAC
TATATAATACTTTCAA
>Cilp|ENSMUST00000048762.7|PolyA_1 location: chr9(+): 65280536 site_id: 452653, AMPLICON
GCCCCCACGATGACAAAACAAAGATTCTCCTCTTAAAAAGAAACAAAAGGAATTGGTAAA
CGTCTGCAGCTTTGGCTGAAAACACACATCTTATTGTATGGTTTTGCCAGCCTTTACACT
AATGATAATCTAATACTGAGGCTCAAGTGAACAACAATAAAACATTTTTCTTGGCCATGT
TCCACAAGATGTAAG
>Cnmd|ENSMUST00000022603.7|Reference_end location: chr14(-): 79637726 site_id: 474151, AMPLICON
GGGCATGGTGTAAATCCAGTTCATCTATCAGGACTGCCAAGCAAGAATCGATATGAGAGT
TGAGAACCAAAGAAGCATAGAGCATGTCCATGCCAAGAGACCACAAAGTATTTTATATTC
AACCTGAATAATGTTATCCTAAACGCTGTTGTGCACCACTGTGTGCAAATGTGCTGAAAG
GGTAGTTTAACTCTACAATTATTATCATTTTACGACTTGCTTTACCAATAAAGCCTACTG
CAAGTCTTTA
>Col10a1|ENSMUST00000105511.1|Reference_end location: chr10(+): 34397085 site_id: 456076, AMPLICON
GCAGAAAGTCCACATACCTGGTGTAACTTGGAAACAAGTGTCTGACTTATGTCAGTAACA
CAGTGCACAGTGTGATTACTCTGATTTAATTCCTTGCATCTTTGTAAAATGATTTTATGG
GCTTATACAATATTAGAATAGACACTTTGTGTGCCTTTCAATCGAGTGGAAGAATAAACA
TTAAGGGTTTGAACATCAGACTAGAAACGAAAAGACATTATTTATTTATGCTCTGTACTG
TATTTTTACATTATTGTTTAAAACTGGTAGGCTGTACCTCACTTATTAAAGCCCGGAGTA
TTTAATCTACTTGGTATTACGAAGCAATAAAATGACATGAATAGACCTTTA
>Col12a1|ENSMUST00000071750.12|Reference_end location: chr9(-): 79598992 site_id: 453294, AMPLICON
GCCTCGTAGCCGAAGTTATTGTATCAACTGGATACTGTTCCATATCGCTTTTAGACGTCT
TTGTTTTGACACACTAACATTTATGCCAAATTGCAGATTATTCTGCAGAAATGGAATTGC

ATGTTTGTGTTGTATATTTAGAATGAACttttttttttttttttGCAAAACATGTTTCT
TAGTTATCAAAGCAGTTGGAAATGTTTGCAAGACTATGAACATAGAATTGCTGCTTTTAT
ATTTTAACTGCAGATTGTGAATTTCACTGCCTTATATTATTTATTTCTGAAACAAAAGAG
GCATTTTTCAATAAAACTACTGAAAATTTGA
>Col22a1|ENSMUST00000159993.7|PolyA_1 location: chr15(-): 71798477 site_id: 475009, AMPLICON
GTTATCCTTGGTGCTTCAGGCTACCAGAACTCCAAAAGAAGTCACATAGTATTGGCAGTA
GCTTGTGTCAGGGCCCTCTATGAACAAGCTGGTGCTGAAGAAGAGTTCCCAGGTGTGACT
TCACTTCTAACTGTGGCCTGAACCCCTTCAAGTTCCTCATGCTGGTTTCAATTGTGTGAG
TTGCAAAATAAAAGTTTTCAATGGTC
>Col2a1|ENSMUST00000023123.14|Reference_end location: chr15(-): 97975602 site_id: 476624, AMPLICON
GAATGGCTGACCTGACCTGATGATACCCAACCGTCCTCCCCTCACAGCCCGGACTGTGCT
CCCCTTTCTAAGAGACCTGAACTGGGCAGACTGCAAAATAAAATCTCGGTGTTCTATTTA
TTTATTGTCTTCCTGTAAGACCTCTGGGTCCAGGCGGAGACAGGAACTATCTGGTGTGAG
TCAGACGCCCCCCGAGTGACTGTTCCCAGCCCAGCCAGAAGACCCCTACAGATGCTGGGC
GCAGGGACTGCGTGTCCTACACAATGGTGCTATTCTGTGTCAAACACCTCTGTATTTTTT
AAAACATCAATTGATATTAAAAACCAAAAAAAAAAAAAATCATTGGAAAGGA
>Cp|ENSMUST00000091309.11|PolyA_1 location: chr3(+): 19992898 site_id: 412179, AMPLICON
CAGAATCACTTGACACAGCCCACTTATCTGCACTGAAAGGActctggctctttaagctct
cacctttcttattctcatctctagccctctttctcctccccaccccaacatggctatggc
cagtctctcttctctttctccctgccttcctacaataaagctctaaaaccataaaaaaaa
tattaaaaaaaaaaaaagaaaaGG
>Ctnnb1|ENSMUST00000007130.14|Reference_end location: chr9(+): 120960484 site_id: 1315788, AMPLICON
CAGATAGAAATGGTCCGATTAGTTTCCTTTTTAATATGCTTAAAATAAGCAGGTGGATCT
ATTTCATGTTTTTGAACAAAAACTTTATCGGGGATACGTGCGGTAGGGTAAATCAGTAAG
AGGTGTTATTTGAGCCTTGTTTTGGACAGTATACCAGTTGCCTTTTATCCCAAAGTTGTT
GTAACCTGCTGTGATACAATGCTTCAACAGATGCGGTTATAGAAATGGTTC
>Ctsk|ENSMUST00000015664.4|Reference_end location: chr3(+): 95509336 site_id: 414963, AMPLICON
CGCAGCGATGCTAACTAAGATTGTTTCATTTCTCCTCTCGTTGGTGCTTCCAGTGACAAC
TCTACTTCCCTTCTCTCTGCCCAGGGCCCTTTTCTTTGTGGACACAACAGGGCATTTTTC
TGAGAGTTGTGGACTCTGTGCTGGTAGACATTGGAGTCCTCCAGCAGGCTGGAGGACTAA
GGTGACCTTCCCGAGCCCCTGTCTTCTGTATACACCAGTGAACATTTCAGTCTTCCACTG
AGATGCACAAATCTATTCGTGATTCTTTGACAAATTTACATGATATTAAAAAAAGTGTTT
TTCCTTCTTTGTATT
>Cxcl12|ENSMUST00000112871.7|Reference_end location: chr6(+): 117181364 site_id: 1295749, AMPLICON
CAAATTCCCCCAGCAGACTTCTACCCTCGCCAAGTTCCCAAAACCCACTCAGCAAAGTTG
CCAACCTCGACGGGCTAGCAGTGTCTAAGCAGCGATGGGTTCAGTGTTGTGTGTGGTGAA
TACTGTATTTTGTTTCAGTTCTGTCTCCCAGATAATGTGAAAACGGTCCAGGAGAAGGCA
GCTTCCTATATGCAGCGTGTGCTTTCTTATTCTTATTTTTAATATATGACAGTTATTTGA
GAAGCCATTTCTACTTTGAAGTCATTATCGATGAAAGTGATGTATCTTCACCTACCATTT
TCCTAATAAAGTTCTGTATTCAAA
>Cytl1|ENSMUST00000073554.3|Reference_end location: chr5(+): 37739817 site_id: 425287, AMPLICON
CAGCTCAGCTCCTAGGTGTCTCATTGGAATGTAAGAGGCACAAAGAGGAAAGTGCACACT
GGCTTCGCTTTGGAGAGCAAGCACCTTAGGAACAGCAAAATCTCATGCCTTTGTGACTGT
TTTAATGAACTAATGGGACCACTCTTCTTTCTGGTCTCTGCTTACACCTACAGGGGCTTC
AACTTTATGCTTCCTTCTTCCTGTGCAAGCTTTCCTGCCTCTCTCTCATTTTAAAGTGTT
TTTACTGCTTTTGCGATACATTTACAAGGCTTTTATGTAGTGTAAACGAGCCACCCTTTC
GCTGAAGGGTGATGAAAACCAAATAAACCTCTGTCGTG
>Dkk1|ENSMUST00000025803.8|Reference_end location: chr19(-): 30545863 site_id: 486504, AMPLICON
GGGCAGAATTCAGAAGAGATTATGCAGAAGGAAGCCTGCCAACTTAAGCCAAATAATTAA
TTCTGAATATTTTAGATCCTCTGACATGCTGAAGAATTGTTTCAATCACTTTGATTTAAA

AAATAATTACTAAACAATCATAAATTCTAGAATGTAACACTTCTCTTGCCAAGAAATTAT
TTCATTTTTTGCTTCACCTGTACTCCATTGATTGGAATGAGAATGGCTTTATTAAAAAAA
AAAATCCCCGAATTGAA
>Dkk2|ENSMUST00000029665.6|Reference_end location: chr3(+): 132180301
site_id: 416708, AMPLICON
GAGTTCCAGCTTCGGACACACATTTGGTCAGGATGATGGTGGTTAGTGCTCTGCATGTGT
CTGCTAGAACAAAACTCATAAGCTAATTTACCCAAGAGCAAACCTTATGTGGTTTAAGCT
TTGTGATGTGAAAATGGAGTATTTTGGTCAGAATTTCCTACATTTCATACGACACCTACC
ACCTAGTTTTCGCATTATTCCCCTTATATTCACCCTCTCAAAAATTATTATTTGAAATAA
TTTATTTACAGGAAATGTTAATGAGATGTATTTTCTTATAGAGATGTTTCTTACAGAAAG
CTTTGTAGCAGAATATATTTGCAGCTGTCAACTTTGTAATTTAGGGGAAATGTATAATAA
GATAAAATATATTAAATTTTTCTCTTTCAAAAAGT
>Dkk3|ENSMUST00000033036.6|Reference_end location: chr7(-): 112116027
site_id: 443451, AMPLICON
aattgttcaaatccattcTGTTCCAAGACATGGGAGCGCTATGTGCTAAGTCTTCCACAT
AAGAGCACCGAGTACCTCTTAAACGCCTGTAAATCGCATCTGAAGATACCACAGTAAAGA
GATGTAAACATTTAGGAAAACAATAAATGTAACTGATGAA
>Dkk4|ENSMUST00000033936.7|Reference_end location: chr8(+): 22627547
site_id: 446421, AMPLICON
aggcgagcatgttacactcagcACATAAATAAAGCCCAATCTCCTAGCAACTGAGACAGA
CAGCAAGCAGATACTGAGATGCTATACTCTGATGAGCCAGCGACTTTTGGCTTTGCGTCT
ACTATCTACAGTACTGACAGGACTTTACAATAAAACAGCCCGGACACAGTGACTCAATCC
GTTCAAATAAATCTTTTTTAAAAATTGAAA
>Dvl1|ENSMUST00000030948.15|Reference_end location: chr4(+): 155859296
site_id: 423612, AMPLICON
TCTAAGGGTGGCAGCTAGTTACCCAAAGGGGCAGTTTGCATGCCCCTTTCCCCACCTGCT
ACTTGGCACATGACAACACAGTTTGTACTGAAGGTATGTGAAGGGTAGCTAGTAGGAGAG
ACAGGAGAGAGACCTGGCACCTAGCCACTGTCTCAGTCTCAGTGGTGGGTGACAGTGAAC
ACAAGAGCTGCAGAGCTGGGCCCTGTTCTGTTTCTGTTCTGGTGGCTGCCCATCATCATG
TGCCACTGCCATCCCGGCACAGCGGCCCCACACATCTACACTAGACACTGTGTCAAAGTC
TGAGTGACTGGGTAGTTGACATAGAGCTGCTTCTGTGTAAATGCTGCTTCTGTGTAAATG
CTATTTTAAACACTAAAAAAAGCGTTTAATT
>Dvl2|ENSMUST00000019362.14|Reference_end location: chr11(+): 70010108
site_id: 461820, AMPLICON
TCCAGGTGCCTAGTCAGCTCTCATGTTGAGGGTGGGTGGTGGTCAGTGGTACCCTGGGAA
GAAGAAGGTGTGGGCTGGGTACCCCAGACAGCAGTCCAAGGAGGCTGGTGGTGTGTTGGG
GACTGTGTAGCTGCCTTTGTTACTCTATTTATTTTAGTCACTTGTACAAAACACCAAATA
AAGCAAATAGAGG
>Ecm1|ENSMUST00000117507.8|Reference_end location: chr3(-): 95734149
site_id: 415002, AMPLICON
GCTGTGACCTGTCTCCTGAAGATAAGCAAATCAACTGCTTCAATACCAACTACCTGAGGA
ACGTGGCTTTAGTGGCTGGAGACACTGGGAATGCCACTGGCTTGGGGGAGCAGGGCCCAA
CTCGGGGAACAGATGCCAACCCCGCCCCTGGGTCCAAGGAAGAATGAGTCACCCTGAGCC
TCAGAGGATTAGATGGGGGAACTCCGCCTACTCCACCCTCCTCGAACACTCATTACAATA
AATGCCTCTTGGATTTGGC
>Epyc|ENSMUST00000020094.7|Reference_end location: chr10(+): 97681900
site_id: 458209, AMPLICON
ACTCATGTATGTTTCCCACCTGATTCAGTTAAGAGCAGAAAAAAATATTAAATGTGTTTG
AAAGATATATGGTCTTGTGAAAAAGTATTTCTTATAATTGTAGTGTGCAAATAACTATGT
AATAGCAGTATACATTCCTTCAGTTTCTAATGACATCTATCATCTATAAGAACATCAACT
CATTTTGCAAACTTGGTTAGTTGACAGCTACCTGTATTCCTCTACATACAAAAAATAAAA
TATGAGATATAGAGC
>Esm1|ENSMUST00000038144.8|Reference_end location: chr13(+): 113218097
site_id: 471182, AMPLICON
CACAAGAAGAACTGTTGTCCTGCCAATGCTGGGAGATATTTGCAGAGTATTTTATTTGGA
GTGAAGAACTTATTTAAGAAGTATTTGCTTATTTCCTTATATTTTATTCTCAAACCTTGC
CAACAGAGTTGTGAATGTACTCGTGGGAAGATCCGTGAATGTAAGCGCAACCGGCTGTTA
GGTTTTGTCTTAAAAGAACGGTGTATTATTGTTCAATAAAAATGGCACAGCTGT

```
>Fbln5|ENSMUST00000021603.8|PolyA_1 location: chr12(-): 101749960 site_id: 1111138, AMPLICON
TGCTGCTTTGCAAAAGTCCTCATGGGCTCGTGGGAAATGCTGGGAATAGCTAGTTTGCTT
CTTGCATGTTCTGAGAAGGCTATGGGAACACACCACAGCAGGATCGAAGGTTTTTATAGA
GTCTATTTTAAAATCACATCTGGTATTTTCAGCATAAAAGAAATTTTAGTTGTCTTTAAA
ATTTGTATGAGTGTTTAACCTTTTCTTATTCATTTTGAGGCTTCTTAAAGTGGTAGAATT
CCTTCCAAAGGCCTCAGATACATGTTATGTTCAGTCTTTCCAACCTCATCCTTTCCTGCA
TCTTAGCCCAGTTTTTACATAGACCCCTTAATCATGCTTTCTTAAGAGTTTTTACCCAAC
TGTGTTGGAAGACAGAGGTATCCA
>Fbn1|ENSMUST00000028633.12|Reference_end location: chr2(-): 125300599 site_id: 1272890, AMPLICON
GTCATTCCTGTCAACAAGGGGTTAATGTCATGATGTCACAATAAAGTGATCCTCCCCTCC
CTCCCCATTTTAGTTTACACCTTGGTTTTATGTTCTTGTGTGGATTAAGGCTGGAGGGGC
CTTTCTGGAGGTGAAATAAAGTCTCCTGGGTTTAA
>Fbn1|ENSMUST00000028633.12|PolyA_1 location: chr2(-): 125300943 site_id: 1272891, AMPLICON
TTGCTTCTCCGAGTGCAAGCAGTAGGTTTCTGTTGTCTGTTTGGTGCTAGTAAATTCTCA
AAATAGATTTATTGATGCACTGTAATAAAGACACGAGTTAGGAACCAAATTGCCAAGTAC
TCAGTTCAAATACTTCATTTCAATCAACCAAAGTTAGTTCAGTAGCTTATCTCACTTATG
AGTATGGTGCAGTACATGTAAATTAAGTGTGTGTACACTGTAACGTGCTATTTTTATCAT
TGAAACATTTATAAACTACAATAATAAAGCCCTTAATGT
>Fzd1|ENSMUST00000054294.6|Reference_end location: chr5(-): 4753896 site_id: 423783, AMPLICON
GCAGTCCTCCTGATTGTAGTGTTTAAACCTTAAGAGTTTATCACAAATGCCGATACATAC
ATAGGACCTAAATTTATCTATGTCTGTCATACCCTAAAATGACATTGGTTTTTGGATTTG
GTATGCattattattgttattattgttattattattCTCACCACCATGAGATCATCTATA
TTTATAGAGGAATAGAAGTTTATATATATAA
>Fzd10|ENSMUST00000117102.3|Reference_end location: chr5(+): 128604098 site_id: 429051, AMPLICON
GGCAAGAAAAGAAATCATCTAACAAAAGAATTTAGAGGCTCAAGCCTCAGGGTTGGGGGT
TGGGGGGGCGCCTTCAGCCTGATACATTTTGTGGCTTTTTAATGGAAACCAAGCCAGTGT
GTTCTACACACTGGGACTGATTTGTGGAGAGGAAGGGGGAGGGGGGGAAGCCAGTGGAGA
GCACCAGAGGGCTTATTGACTCTCTGAATTGTTAAACAAATGATTTCCATGAGTGGTCTT
GAAGCACTTGGGAAGACAAACTTTGTCTCCAGCCAGGGAGGGCCATGCCTGCCTCTGTAT
ATCTGTAATATAGGATATTTTTCATGCTCCACTATTTTATTAAAAATAAAAGACATTCTT
TAGTTT
>Fzd2|ENSMUST00000057893.6|Reference_end location: chr11(+): 102608056 site_id: 464355, AMPLICON
AGTTAGCGGCCTGAGAGATGTTCTATAAAGTATCTTTGTTAGTGTGAGAATCCCAGTTTC
CAGCAGGCATGGGTTCCCTCGAGCCTGGAGTACTTTACAGCTTGCATGCTAAAGAAGCGA
TCTGAAGACCAGTGTGCCAGAGCTCCGCTCTCAACCGTCTCTCCTGTTTCTTGATAATAA
ATAGTGCAAATTAAAACCTAATAATCATCATAATAAAGTGCATTTAATTATCT
>Fzd3|ENSMUST00000131309.2|Reference_end location: chr14(-): 65192449 site_id: 1331290, AMPLICON
CTTCTCCATGTGTCGGCTAAAACAGATCATTTCCAGAAGAGTGGAAAGTTACTGTTGTTG
GGGGTCTGTGTAGGGCGGGCACACCCAGTTAGAGCATGGGCTGTACTGTCACGGTAGCTG
GGTGTGCTCTGCACTCTGCTGTTGTAGATTTATTTTGTTTACAGTAGACTGATGTCAGTT
TTGTAAATGTTTTTCTTAGCACTTTAACTGTGAGTGTAAAAACTGATTTTAAATGTAGTG
GTTGTATATTTTGGTTATAAATTGGAAATTTTTAATAAAACTAAATAACTTGTT
>Fzd4|ENSMUST00000058755.4|Reference_end location: chr7(+): 89413093 site_id: 441958, AMPLICON
ACCAGAAGGCAACCTGGGGATATTGAGATGACAATGTAGAGGTACCAGGCAAACCCCTGC
TATACATTCTCTGAACAAATCACTGAATTTCCAAGGAAATAGATCCCCCTTTAAAGGATG
TACAAAAGTGTGTCTGCATTGATGTCTGTACTGTAAATTTCTAATTTATCACTGTATTAA
AAAAAACTTGCTAT
>Fzd4|ENSMUST00000058755.4|PolyA_1 location: chr7(+): 89409399 site_id: 441959, AMPLICON
TTACCTTACCGACCCTAGAGACCTATTGCATTAAGCAATGTTAAGCAATTGGGACTTAAA
```

```
ATATTTTAGTTTGTGTGATTGCATCTAGGCAGACGCCAGTCTGGAAGAACTGAAATGTTA
AATTTCTTGGCAACTTTGCATTCACACAGATTAACTGTGTAATTTGTGTGTGTCAATTAC
AATTAAAAGCACATTCTTGGACC
>Fzd5|ENSMUST00000063982.6|Reference_end location: chr1(-): 64730566
site_id: 399323, AMPLICON
GTCTTGGAGAGACGTTTGGTTTGGTTTTGTGCCTGGGAGTCAGTCAGTACAACAGCAACT
GTATGTGCTTATGTTAGTCTCAAGATCCTTAACTTGTTGACCTTATTATTTTGGAAATTT
TTTTGTATTATATTTGTGGGAAGGTAAAATAATGATTTTAAGATTTTTATCAAATATGGA
GATTAGTTATTTATGAAAAACAAAGAAATGTCTATTTTTGTTTCTTTGTTCCCAATTAAT
GTAGATAACTTTTTAAAATGCATTAAAGCAATGGTGAAGAA
>Fzd6|ENSMUST00000022906.7|Reference_end location: chr15(+): 39038187
site_id: 474976, AMPLICON
ATTCCTATCTGCATCTAAGCCTGCAAAAGAAAATGTGCGAAGGGCAGAGTCAGAGTTGGG
CAGGAAGAGTGTAGTGCAGCAGATGCAGCGTGAAGACACTGAAGGTGCTAAGACAGCGTC
TCAGTGCTGGTCCTCCTTAAGGATTATCTCGCCAGCGAGGTTTTCTTAGATACTTTGATC
CCATTGGAGCTCTGTTAAAGTTTAAAATGAAAATTATCATGTACTGTATGGGAAATGTAA
ATACTAACTTTTCCACATATGTAAACTTCAGACACAAATTTTTTTGTGTGTTCTTTTCAT
CAATAAAATTTTCTTTGT
>Fzd7|ENSMUST00000114246.3|Reference_end location: chr1(+): 59486945
site_id: 1110103, AMPLICON
GACAGATTACTTCATATGTGTCCTGTTCAGTTGAATGGAGCTGCTTTTACAATTAAAGTG
ATCTTGTATTTTTTAAACTTTCAAAGTGATCTCACCTGTCAGAATTTTTTTAAGCTGCCA
CTACACAGGTTTGGCATCTTTTGTGTTTTATCTCTTTAAGTGCATGTGAAATTTGTAAAA
TAGAGACAGTGCAGTATGTATATTTTGTAAATCTCCCATTTTTGTAAGAAAATATATATT
GTATTTATACATTTTTACTTTGGATTTTTGTTTTGTTTACCTTTAAAGATCTACAATGAA
GCCCCACTTTATCACATGTACAGATCACGAATAAATTT
>Fzd8|ENSMUST00000041080.6|Reference_end location: chr18(+): 9218137
site_id: 1327188, AMPLICON
TGGCTGCGCAGATACCACTCTAACCTATGTAGCTGTGTTCTTGACTACTACATGAAAAAA
ATGTTAAGACTCATCAAATATTTGAGGGTAGGCAATGTGGTTGTTATATGTCCAGAGGAT
TGAGGTAAATATAATCTAATGTGATTAAAACAAACAGGAAAC
>Fzd9|ENSMUST00000062572.2|Reference_end location: chr5(-): 135248938
site_id: 429404, AMPLICON
CTAGGCTGTGAGTTATGGTTGCTCCCTCCTTGCCCTCCCCTCCCCCCTTCAGAGACAGCT
GACTAACAGCTGCCCAGCTGTCAAGGTCAGACAAGTGAGACACAGGGGGCTGAGGACTAG
GGTGGGGACCCAGTAAAGCTCAGGGCCTTGACCTTCTGTCTCATGCAGGGAGTGGTCCTA
GTCCACAGAGGGTCCCAGGATAAGAAGGGGCAGAAGGGGGCAGGGTCCAGTGCAGAGTTA
TTTAATGATGTAATTTATTGTGGCATTTCTCTGGAAGCTGTGACTGGAATAAACCCTCGT
GTGGC
>Gja4|ENSMUST00000053753.7|Reference_end location: chr4(-): 127311425
site_id: 421568, AMPLICON
GGTTTTCATCACACCCCACTTCTCCTGGGAAAAAGCACTGATGCAGGCTGCTGGCTCAGC
CTCGTCACCTGGACAGACACAGCCTGCGCCGGAGCTGGCCCTTGCCAGGGGAACTGGTTG
ATGTCTTGTTTTTCTCACTTCTAGTTCCACTGTTTATGATCCTCAAATAAACAGGACTCC
ATCA
>Gsk3b|ENSMUST00000023507.12|PolyA_1 location: chr16(+): 38245439 site_id:
1123014, AMPLICON
CAATTAGCGTGACCACTTCCATCTTAAAAACAAATCTAAACAAAATTTATTATTATTATA
TATATATAAAGGACTGTGGGTTGTATACAAACTATTGCAAACACTTGTGCAAATCTGTCT
TGATATAAAGGAAAAGCAAACGCTGTGTAACATTACTACTTGAATGCCTTTGTGACTGAA
TTTTTTTTTTCATTTTAAATATAAACTTTTTTGTGGAAAGTATGCTCAATGTTTTTTTCC
CCCTCCCCCCCATTCCCTTGTAAATACATTTTGTTCTATGTGACTTGGTTTGGAAATAGT
TAACTGGTACTGTAATTTGCATTAAA
>Gskip|ENSMUST00000051934.6|PolyA_1 location: chr12(+): 105701922 site_id:
1111387, AMPLICON
GCAGTGCTTTGGTATTCCTACAGCGCCCTTCTTCCCCAGCAGTTGGACAGTGCTCTGTCT
CATTGCCACATGAACAGTGTAACATGAGTGCATTGTATGGTTCGAAACCAAAGGATGAAT
GAAGCATTCAGAAACTTGATATTTAAAAAAGAGATGCTCTGTATTTTATATTTTATTACA
```

```
GTCCCCGGTGTTTATAAACGTAATAAAGTCCTCCGTGCTG
>Hey1|ENSMUST00000042412.4|Reference_end location: chr3(-): 8663382
site_id: 411912, AMPLICON
CAACCCGATTTTCCTAGAACTCCCATATTTTCTTTTAAGTGGAAATTTTTATGTTGTGTT
TTCCTTTTGGTGCATGAAAATGTGGTTCTTGCAGTACTTAAAAGGGCTTCTCTGCCTTCT
CATTCATTTTTAAAATTTTGATTTGGGCTCTAAAAGTATTGTTTTACAGGCTTACCCCTT
TAGAAGGTATAATTTGAACAGCTCCTCTGAACTAGGTTTGACCTCTGTTGTATTGATGTG
TTGTGACTAA
>Hlf|ENSMUST00000004051.7|PolyA_1 location: chr11(-): 90338566 site_id:
463297, AMPLICON
TGTTCGCTTTCGGCCTGTGTATCTATCTCACGCAGTGTAAGGTTGACCTCGGTGCTATGT
GTACGGCTTAGAGTTTGATAGCTGTTTTGACTTTAAAGATGGCTGTTATTTTGTTTCACT
GAGTTGTATAATGTCAAGAGAGTCTGCTGTTTCTTCAAAAGCACATTTTTGTGCTCGATT
AAAATCCCCTTCCATCC
>Igfbp7|ENSMUST00000163898.5|Reference_end location: chr5(-): 77349266
site_id: 1288324, AMPLICON
GATGCCCTCCATGAAATACCACTGAAAAAAGGTGAAGGTGCTCAGTTATAACCTGCGAAT
CCATGAGCCTCTGTAGCTAAAGGTGCTCTCAGACAGCCGACAGCTATAACCCTGCTCTTG
CCTGACACACTTCTCTTAACCTAACCCACTAACACTTTATTACAGCCAGCTGGTTTTACA
CAGAGAAATCAAAGATAACACATCAAGACTATCTACAAAAATTTATTATTTACAGAAAAA
AGCACATGTAGCTTTAAACAAAACAA
>Kcnk2|ENSMUST00000193319.5|Reference_end location: chr1(-): 189209790
site_id: 403939, AMPLICON
GACTGGCTACGGGTGATCTCTAAGAAGACGAAGGAAGAGGTGGGAGAGTTCAGAGCGCAT
GCCGCTGAGTGGACAGCCAATGTCACGGCCGAGTTCAAGGAAACGAGGAGGCGGCTGAGC
GTGGAGATCTACGACAAGTTCCAGCGTGCCACATCCGTGAAGCGGAAGCTCTCCGCAGAG
CTGGCGGGCAACCACAACCAGGAACTGACTCCGTGTAGGAGGACCCTGTCTGTGAACCAC
CTGACCAGCGAGAGGGAAGTCCTGCCTCCCTTGCTGAAGGCTGAGAGCATCTATCTGAAC
GGTCTGACACCACACTGTGCTGGTGAGGACATAGCTGTCATTGAGAACATGAAGTAG
>Kdr|ENSMUST00000113516.1|Reference_end location: chr5(-): 75932835
site_id: 426463, AMPLICON
ATGCAGCTATATACCCTACCGTCTCTCTCATCTCAAACggaggaggaggaggaggagTCA
GGTATAATGTGAGTGTGTTCTACGTGTCCTTGTTCTCTGTTCTTAGGAGGAATGATTTCA
TCAAATGTTTATATGCTTTATAAACCAATAAACGTATTCTGAGT
>Kit|ENSMUST00000005815.6|Reference_end location: chr5(+): 75656718
site_id: 1288216, AMPLICON
ATGGATGTCAGGTACTTAAGGGGCCACACCATTGAGAATTTTGTCTTGGATATTCTTGAA
AGTTTATATTTTTATAATTTTTTTTACATCAGATGTCAGATGTTTCTTTCAGTTGCTTGA
TGTTTGGAATTATTATGTGGCTTTTTTTGTAAATATTGAAATGTAGCAATAATGTCTTTT
GAATATTCCTGAGCCCATGAGTCCCTGAAAATATTTTTTATATATACAGTAACTTTATGT
GTAAATAATACGCTGTGCAAGTTTAAACATGTCACGTTACATGTGGGTTTTTTCTGATAT
GTTGTCCAACTGTTGACAGTTCTGAAGAATTCTaataaaaatgtaaatatataaa
>Kitl|ENSMUST00000105283.8|Reference_end location: chr10(+): 100100402
site_id: 1319857, AMPLICON
GTGATTATTGTGTCTCTCTACCACTGTTTTTTAAGTCAATGGACTTGAGAAGTATTCTGA
CATTTATATGGCTTCACAGATATATTGCTAGTTCAGTCATAGATTGGAGTTTGCATATTG
TAATGTAAGTGTATGTCCAACACTATTCTAAATAGTTTATGACTGAAGTTTAATTAAATA
AAAGGTTGTAAAatgtgatgtgtatgtgtatatactgtatgtgtaCTTTTTAAAATAGGT
ATATGTCCCGACCCTTTTGATACAGGTTTGAATTTGAAATTACATTATATAAACATATAC
TTTATTGTTCTAAATAAAGAATTTT
>Kitl|ENSMUST00000105283.8|PolyA_1 location: chr10(+): 100100272 site_id:
1319858, AMPLICON
GTGATTATTGTGTCTCTCTACCACTGTTTTTTAAGTCAATGGACTTGAGAAGTATTCTGA
CATTTATATGGCTTCACAGATATATTGCTAGTTCAGTCATAGATTGGAGTTTGCATATTG
TAATGTAAGTGTATGTCCAACACTATTCTAAATAGTTTATGACTGAAGTTTAATTAAATA
AAAGGTTGTAAAatgt
>Lef1|ENSMUST00000029611.13|Reference_end location: chr3(+): 131224320
site_id: 416684, AMPLICON
```

ATCTCCTTTGAGCGAACCCGTTTGCAGTTCATTTTTTTAATCTCTTTCTTTTCACTCCTG
TAAAAAGCCCAGCACTTGAATTGTTCTTTCTTTAAATGTTCTGTGTTTGTATCTGTTTTT
ATTAGCCGATTAGTGGGAATTTATGCCAGTTGTTAAAATGAGCATTGACGCACCTGTTAT
TTTTTTTTTTTTAAATAGCAAGGCACAGCCTCTGCCCCAAACTTGTCATCTTAACGTTTG
TCATTCCAGTTTGAGTTAACGTGCTGAGCATTTTTTAAAAAGAAGCTTTGTAATAAAACA
TTTTTTTTAAAAG
>Lepr|ENSMUST00000102777.9|Reference_end location: chr4(+): 101792258
site_id: 420218, AMPLICON
ATGACGCAGGGCTGTATGTCATTGTACCCATAATTATTTCCTCTTGTGTCCTACTGCTCG
GAACACTGTTAATTTCACACCAGAGAATGAAAAAGTTGTTTTGGGACGATGTTCCAAACC
CCAAGAATTGTTCCTGGGCACAAGGACTGAATTTCCAAAAGGTCACTGTTTAAGTATTTT
AACCCAGATATCTAAGGTTGCAGTTTAGATGCCACAGTACTTACAGATCTTTAAACAACT
TTAAAGGGCTTTATGTTGTTGTGTTCATGTTCTCAAGCCTGTTCATCCTTTCTCTTTCTC
AAAAGCTGGGTTTGGGATTTGATCAGAGAAAACAAAGTTCGCTCCCTTA
>Lgr4|ENSMUST00000046548.13|Reference_end location: chr2(+): 110014220
site_id: 1272187, AMPLICON
CCTTGCCTTGTAAATAGTCTTGGTTGTACATTGTCAGTGGAATAAAAACAGAATCTTTGT
ATATCAAAATCACGTAGTTTGTATAAAATGTGGGAGGGATTTATTTACTGTGCTTGTAAT
TTTGTAAGGTCATCTATTTACAACATAAATATAATGTTTGTATATTTACACATCTGATAA
ATATTAAATCATAACTTGGTA
>Lgr5|ENSMUST00000020350.14|Reference_end location: chr10(-): 115450311
site_id: 454434, AMPLICON
CCATGTACAGTAGCCTATTCTCCCAGACATATTGTTTCTCTGCCAATTTCCCCAATGCAT
TAACTGTACATAACACATGTAAATACGTAGTGCTTGTAAATAGATCCAGAATTTGCTTTT
CTACTGGGCTCAAAATAAATTTGTAATAAAATGTGTGACTTGCAAAACAAA
>Lgr6|ENSMUST00000044828.13|Reference_end location: chr1(-): 134983305
site_id: 401712, AMPLICON
AAGAGGTAGTAGGAACCGCCATCTTCCTAGCTCCAGTCCCTCTGACTACCTCTGCTCTGG
GTTACCAAGTTCTCCCACCTAAGACCTACCACCTCCACCTCCTTTCTGGGTTCCAGTCCC
ATGCCTCATCACCAACCCCAGCCATGTGAACTATAACAATTGTCAAGATGTAAATATCCG
GTTTTTCTGTATAGTAAAGCACCTATGAA
>Lrp4|ENSMUST00000028689.3|Reference_end location: chr2(+): 91513767
site_id: 407876, AMPLICON
GTCTTGTTGTCATTAGCTAGGCCCAGATATATGTCTGCACACAGAGCCAGAGCAGGCCAA
TGGCCTTTTCCAGTAAACCAATTGAGAAAAGTACAGTGCCACTTCTAAGAGGAAAGATGG
ACATTTCACACAGAATGAGGCAGCACTTGAGCCTTTAAATCCATAGTTGGGGAGGAGGGG
GGGGGGGGGAAACAGAAGCGTGGTGTCCTCCTGAGTGGAAAGGGGCAAGGATCTTTTTGC
ACTTGGGCTGTTCAGAATGTAGAAAGGACGAATTTGAGGAAATATCTATTTGAGCACTGA
TTTACTCTGTAAAAAGGGCAAAAATCTCTCTGTCCTAAACTAATGGAAGC
>Lrp5|ENSMUST00000025856.16|Reference_end location: chr19(-): 3584831
site_id: 1249376, AMPLICON
AGCAGCAAATACTACCTGGACTTGAATTCGGACTCAGACCCCTACCCCCCCCCGCCCACC
CCCCACAGCCAGTACCTATCTGCAGAGGACAGCTGCCCACCCTCACCAGGCACTGAGAGG
AGTTACTGCCACCTCTTCCCGCCCCCACCGTCCCCCTGCACGGACTCGTCCTGACCTCGG
CCGTCCACCCGGCCCTGCTGCCTCCCTGTAAATATTTTTAAATATGAACAAAGGAAAAAT
ATATTTTATGATTTAAAAAATAAATATAATTGGGATTTTTAACAAGTGAGAAATGTGAGC
GGTGAAGGGGTGGGCAGGGCTGGGAAAACTTTGTACAGTGGAGAAAATATTTATAAACTT
AATTTTTTAAAACA
>Lrp6|ENSMUST00000032322.14|Reference_end location: chr6(-): 134446500
site_id: 435949, AMPLICON
TCATTGTCTTAAAGTCAGCTGGTGTTTCTTGTACATCGACTTTGGAATGATGGTGCTTTG
CAAGGATGTATTTATATTATAATGGCCAACATTTGGTCAGCCCTTGTCCGCTTATTCACT
TCCCCCCTTTTGTAAAATAAGTGCTTTAATTATAAACTGTATAAATACCTTGTATAACCC
CCCCCCCTTTTGATTATTACAAACAGCTGAATTGTAACAAATGAAATGTTGATTTTTATA
ATAAAACAGTGGA
>Mafb|ENSMUST00000099126.4|Reference_end location: chr2(-): 160363708
site_id: 1274317, AMPLICON
ACTTGCCGCAACGCAACAGAAATTGTTTTTAATTTCATGTAAAATAAGGATCAATTTCA

ACCCCCTGCTTATGATATGAAAATATTAAAATCTAGTCTTATTGTAGTTTTAATTCAGAC
TGGTTTCTGTTTTTTGGTTATTAAAATGGTTTCCTATTTTGCTTATT
>Matn1|ENSMUST00000102576.3|Reference_end location: chr4(+): 130955475 site_id: 421811, AMPLICON
CGACTGCGTTCGTTATTTTTCCAACCTAAAGCTTAATATATTCCTGTTTTTTTTTTCCTC
CAGTTAATTCTTTACCCAGTCAGTTCTTTACTCTGAGAGATTTTTTTTTGTTTGTTTCT
TGAGAAAAAAATAAAAACAATTAAATGTGGTTCGCTGTACACCCTG
>Mecom|ENSMUST00000173495.7|Reference_end location: chr3(-): 29951336 site_id: 397140, AMPLICON
AGCTTGTGCGATGATGTTATGTTCATGTTAATCCTATTTGTAAAATGAAGTGTTCCTGAC
CTTATGTTAAAAAGAGAGAAGTAAATAACAGACATTATTCAGTTATTTTGTCCTTTATCG
AAAAACCAGATTTCATTTTTCCTTTTTGTTTGTGATCTCATTTGGAAATAATTGGCAAGT
TGAGGTACTTTCTTCCCATGCTTTGTACAATATAAACTGTTATGCCTTTCAGTGCGTTAC
TGTGGGAGGAGCAACTA
>Mgp|ENSMUST00000032342.2|Reference_end location: chr6(-): 136872441 site_id: 436090, AMPLICON
GTCATTTGGTTGTGGAGTTTCGTTTTATATCTCCTGCAGTAGCATTACTGAAGTATACAG
ACACGCATGTGTTGCTTGCTCCTTACATGATCTCCTAGCTGGCTGGCCCACTCCTTCCTT
CTGCGGGTTGAAAGTAATGAAAGAACAGTATTAAGAAGTGTGTTTATATATAATAAAATT
CTGGTTTGATAC
>Mki67|ENSMUST00000033310.8|Reference_end location: chr7(-): 135689785 site_id: 444897, AMPLICON
CTACTGCTACTGCCTGAGTTTAAGGAAGGAAGCTTTGAGCTTTCCTGGTCATACTCTCTT
CAGACGCCAATGGAGGTCATGAGGAAGATCACCAGGGATCTCAGCGCAATTACAGTTTAG
GGGTGAGCAGGCAGAAATGTGGCCCTCTGTCCTATCCAATAAAGCTCTGAAATTCGCTGC
C
>Mmp13|ENSMUST00000015394.9|Reference_end location: chr9(+): 7283329 site_id: 1310304, AMPLICON
GATCCAGCTAAGACACAGCAAGCCAGAATAAAGACTGTGCCAGCTGGTCAGTCGCCCTTT
TGAGACCACTCCTTTGTGCTCCACCATGTTTGTTAATCCCTCTCTGCTTTCCTTAGCGAG
TAACACTTGGTGCTTACTGATGTGTGAAAAGCTATTGTGTCAAGAGACAGTGTTAATTAA
ACTGGGAAAATACAAAAGAACTGTTTTTTTGAATAATATGTTAGACTGTATTTATGTTGT
TTCTAATAAAAATAAGTGTTTTCAGC
>Mmp9|ENSMUST00000017881.2|PolyA_1 location: chr2(+): 164955120 site_id: 410930, AMPLICON
GAACTCACACGACATCTTCCAGTACCAAGACAAAGCCTATTTCTGCCATGGCAAATTCTT
CTGGCGTGTGAGTTTCCAAAATGAGGTGAACAAGGTGGACCATGAGGTGAACCAGGTGGA
CGACGTGGGCTACGTGACCTACGACCTCCTGCAGTGCCCTTGAACTAGGGCTCCTTCTTT
GCTTCAACCGTGCAGTGCAAGTCTCTAGAGaccaccaccaccaccaccacacacaaaccc
caTCCGAGGGAAAGGTGCTAGCTGGCCAGGTACAGACTGGTGATCTCTTCTAGAGACTGG
GAAGGAGTGGAGGCAGGCAGGGCTCTCTCTGCCCACCGTCCTTTCTTGTTGGACTGTTTC
TAATAAACACGGATCCC
>Myc|ENSMUST00000022971.7|Reference_end location: chr15(+): 61990352 site_id: 1119839, AMPLICON
GTTCCTTCTGACAGAACTGATGCGCTGGAATTAAAATGCATGCTCAAAGCCTAACCTCAC
AACCTTGGCTGGGGCTTTGGGACTGTAAGCTTCAGCCATAATTTTAACTGCCTCAAACTT
AAATAGTATAAAAGAACTTTTTTTTATGCTTCCCATCTTTTTTCTTTTTCCTTTTAACAG
ATTTGTATTTAATTGTTTTTTTAAAAAAATCTTAAAATCTATCCAATTTTCCCATGTAAA
TAGGGCCTTGAAATGTAAATAACTTTAATAAAACGTTTATAACAGTTACAAAAGATTTTA
AGACATGTACCATAATTTTTTTTATTTAAAGACATTTTCATTTTTAAAGTTGATTTTTTT
CTATTGTTTTTAGAAAAAAATAAAATAATT
>Myh11|ENSMUST00000231567.1|Reference_end location: chr16(-): 14194648 site_id: 1422024, AMPLICON
GACTGCTTAGCACTTGTGTCCATTCGTTCTCAAGTCACAGAAATCACTCCACCCCTCACC
AGGAGTCAACCACAGCCCTGCACAAAGGGTGTGAATTCAAGTTCTTAGATGGGCTATCAG
AGAAAAATCCAAATAAGACGTACAGGTCAGGAGCCACAGTCACCAGCAACTTTGTTTACA
TGAAATGAGGAAGTTACATGAATGGGTGCCACAGCCCCCAACCCACTCCCAACCTTTCCC
TGACCTGCCTGACCTTCCAGTTTGGGAGGAAATCCCGTTTGAAAATTCCCTTCCCTTTCC

TGCCTGCCACACCTCAGACACACAGTTCACTTCGCCCAAGTGAC
>Notch3|ENSMUST00000087723.4|Reference_end location: chr17(-): 32120825 site_id: 1461884, AMPLICON
GTCTTGTCTGCTCAAAGCCTCAACTCCAGGCTCACCTCCCAGAGCCTGGCTCACCTTTTA
GGGCCTGGGGTGGGGGGGCACGTCAGGGGAGATGTATTTGTATGCATTCCACTTCTAAT
TGTAAATACAGGGCAGAAGGTGGGAGTGGCTCTCCCTCTTCCTGTTGTTCTCTTGGCTCA
GCCTGCCTAATAGAAATGTTTTTAGGCTGTTTTTGTAATATGGCACCTGGTCACAGTCCT
TTGTAGCTGAATTCCCAGGTCCTGCACTGTACAACCCTCACCTTCTCAGTTCCCTTACCA
CCTAATAAAGGAATAGTTAATAC
>Nrarp|ENSMUST00000104999.3|Reference_end location: chr2(+): 25183329 site_id: 1268681, AMPLICON
GACACTGAGACAAGCACAGATTACTTCAAAAGGAGTTCTGTATGGTTTTTCTACACGCAA
ATGCCTTTTTAATTATGTTAATTAATGTTAAAACGTACTGGGCCTATGTTGAAGCTTCAG
ATGGGTCCTGGTTGGTCTCTTCTATCTGTATGAAGTCTGTGGCACACTCCTAAGCGTACG
ATATAGACTTGTAGCCCATCGTGAAAAATTTATAAATAAATTTTTCATTGGTCTTTTTAT
A
>Panx3|ENSMUST00000011262.3|Reference_end location: chr9(-): 37659902 site_id: 1311444, AMPLICON
AGTCCCATCGATCCACTTCTATACATTCTCCATTCACTGTTTCCTCCTGGCATCTAATTG
TCACAGCTTAGCTCTGATTCAAAAGACGGTGCTCTATCCCCAGTACCCTCGTGACTCTTA
ATAAATATTTTTAAACTGTATATTATGTATTTCTTGAGAGAATTTTTTTATACAAGTCAT
TTAGATCCCTCTTCATATAGGTAATAGAAAAGTCAGGTTGCTTCTGACAAATTCATGTCA
ATTGCCATAAAGCAAGCCAAGAGCTTATAGATGTTCACAGATGACATTAATAAAAGTTTC
ATGTCA
>Pecam1|ENSMUST00000080853.10|Reference_end location: chr11(-): 106654220 site_id: 1326097, AMPLICON
GTCACTTGAAGACAGACCTCAAGCCAGCAATGGCAACTGGAGCGAGCACTTAAAGCGCAC
CTTGATCTTCCTTTCCGTTCCATGCCGAAGGCCCAAAGAAGAGAAATGGCTGGCTACAAC
AACTGTTATTATTTTTTTAATAGTAAAAGGAAATATGTACTTTTCTCACTAATTTTTCTT
TATTTATTCCCCACTAAAGAAACGGTTTCCTAAGGTCTGAGCTGTTTCCCAGGGTGGGCT
AGAGTGGGTGGGCAGGAGCTGCCGACATTTTGTGTACTATACCTAACCCTCCTCGCCTTC
TCCGTTTGCCTCAGCCACCCGGCTCCAGGGCCCCTGAGGGGAGTTGTATTAAATAAAATT
GATTTTTACTCTT
>Postn|ENSMUST00000073012.12|Reference_end location: chr3(+): 54391016 site_id: 1276553, AMPLICON
CACACAGTTACCTTTCCAGGGGAGGCTAAGGTATCAAAAGGGGTGTTCAGTTATACAACA
TGCAAACAAACCTACCAAATTACGAACAGTGGTGTTACATATTTCTCATGCAATGTGGGT
TTCCTGCTAAATTTTGTTATTTTTACACTTGATTTATATCCTCGAGATGATTGTCATAAG
CTTCTTGCAATACAAATGTTTTCTCTCAAACATTTCAATAAAACCATTCTTCAGGTATAA
AGAGAATTACTGCAGAGTTGGTAATTCAGAAAACTCAAGGTTTAAGTTAAAAGTGAGTTT
AGACTTTGGAATAGGACTTCATACCTTTTTTTATTGTTAACAAGTAC
>Prg4|ENSMUST00000161611.7|Reference_end location: chr1(-): 150449887 site_id: 402130, AMPLICON
TACTCGGTGCCCATGAGAGTTTCTTATCAAGACAAAGGTTTCCTCCACAATGAAGTCAAA
GTGAGTACAATGTGGAGAGGGTTTCCAAATGTTGTTACTTCAGCGATAACACTGCCTAAC
ATTAGGAAACCTGATGGCTATGATTACTACGCCTTTTCCAAAGATCAATACTATAACATT
GATGTGCCTACAAGAACAGCAAGAGCAATTACCACACGTTCAGGGCAGACCCTCTCCAAA
ATCTGGTACAACTGTCCTTGAACTGATTGACAAAGAAGAGTCATCAAAATGAAATGAAGA
AAACAGTGATACTTTTTGACATTGAAATACATTTTATTAATAAAGAATGTTGAAATGAG
>Pth1r|ENSMUST00000006005.11|Reference_end location: chr9(-): 110722154 site_id: 454922, AMPLICON
CTTAGCCCCCGCCTGCTTCCTGCCACCACCAATGGCCACTCCCAGCTGCCTGGCCACGCC
AAGCCGGGCGCTCCAGCCATTGAGAACGAAACCATACCAGTTACTATGACAGTTCCCAAG
GACGACGGCTTCCTTAATGGCTCCTGCTCGGGTCTGGATGAGGAGGCCTCTGGGTCTGCG
CGGCCACCTCCATTGTTGCAGGAAGAATGGGAAACAGTCATGTGACTGGGCATCAAGGGG
CTGGACTGCTGGCATGGGCAGATGGACAGATGGACCAAGAAGCCAGAGTTTGGCTGGCTG
TCTATTCAGGACTGGACCAGGAAGAC

```
>Ptn|ENSMUST00000101534.4|Reference_end location: chr6(-): 36714934
site_id: 431626, AMPLICON
CTTTGAAAGTTTGCAGACTGAGAGGAGAGAGGCAGAGCAACGATGTAGTGAAATGTTGAT
CtttgttttttttttttttttAAGATAAGATTGAAACATGAAATCCTTTCACTTTGGCAG
AAAAACATTTGTTTTCTTGATGAAATTATTTTTACATCTGAGGAAAAAAATCTAGGAAAA
TAAAACAAGTGATGCTGAA
>Ptprc|ENSMUST00000182283.7|Reference_end location: chr1(-): 138063422
site_id: 1265900, AMPLICON
ACCTCGTACAGTTAGTTCTGTTATGGAATTCACCATTTATGGGAAATGTAAAATTGACTA
TGGCCATTTCCTATGCTTAAGACCATCTTTGACTTGCATTACTGTGTATTTATCTTGAAT
TTCCCCACTGTTTTGTTTACTCTTACTGAGATATAATATTGATAACCATAATAAACTTTC
AACTATT
>Rgs5|ENSMUST00000027997.8|Reference_end location: chr1(+): 169695790
site_id: 1266827, AMPLICON
ATGTCACAGGAGGGGAGGAATCTGGGTAATGCTTGCCAGACATTCCAGGACACGGAGTCA
TCACGATGTGAGGGAACAAGAGTTCTGGTTTTGTTTTTAATTCTGTAAGTTAGTCTTTTG
GGGGCTAGAATGGAATGGGGAGGGGGTTTATTAAGTCCTGTGCCAGCACTTCGCTCATAA
AGTTTTAAATTCTGGACCAATAGTGTTTGTTTCCTGATTATTGTATCCAATACTTTGCCT
AGTATGTAACTCACTCTTACTGATAATAAAATAAATATTAGTCT
>Rspo1|ENSMUST00000030687.7|Reference_end location: chr4(+): 125009083
site_id: 421445, AMPLICON
GAGGTCACTGAAGACACTTCTATTCTGTGGCCCAGCTGTATATTCAGTCTTTAATGCTCT
TGGAAGACATATCTGAGAGAACCTTTCCCAGCATCTGAAACTAAGGAGTGGAACCTTCTG
GAGGAACTTCTGGGACAGCATCTGACAGATGGATGGCAGATTGGAGCCAAAGCTGGAGCA
GCTGCCGAGAGGGAGAGAGAGGGAAAGCGCTTTCCCGGCTTGAGAGGCACTCCCAGCTGT
GAGACTTGATTGTCGGAGATGAGAATTATTACACATCCGTGGTACACGTCACGGATGACC
TGACTTGGAAACTGCTTAAAGGTTTATTTCAAA
>Rspo2|ENSMUST00000063492.7|Reference_end location: chr15(-): 43020795
site_id: 1331515, AMPLICON
GAAGCCTTTCCTTGCCTTGCATTGGCAAAACTACCTCTTTAGCATTTATGTTGATTCAGA
AACATCTTGCTGATATGTGTAGATGTTTTAAGCTTCATTGTGAAAATATTGATGCAAGAT
AAGCCATATATGAATGTTGTATTCAACTTTAGGGCTTGAAATTAATCCTAAAGTGTTCAC
CTCTCTCCATGTCTATTTACACTCTGTTCCTATTTACTAAGAGGGTAGGGGTCTCCTTAA
TATCATACTTCATTGTTAATAAGTCAATGCTTGTTATGTTTCTTGGCTGTTGTTTTTGTG
CATTAAAAACTCAAAAAT
>Rspo3|ENSMUST00000092623.4|Reference_end location: chr10(-): 29452418
site_id: 1316830, AMPLICON
CTATTGGGAACTGGAGTCACCCTAGCAGGAAGGCTCAGCTATCTTTCCCAGAGGACCCAG
GCAGGCCTGTGCAGAAGGAGAGAATCTCTGAGTGTGTGCTACAAACTCTCATGACCCACT
TAGCCTTCTGCAACGACAGGAGATGTGCAGCCACTCTGGGTACCATGGTTCCAGCCCTCT
CAAGTTCACCGCCTCCTGAAGAAACTGGACTGAGTCTGCCTTCTACCTGTCTGTTTCTGG
ACATCTTTTCTTCACCAACTGTACTGCTCACATGGGCCACTTGGCTTAAATGGTTTTCCA
CTTCTCTTTTAAGACATAAAAGTTTGAAATAGTGTATACC
>Rspo4|ENSMUST00000042217.3|Reference_end location: chr2(+): 151874668
site_id: 410026, AMPLICON
CCTTCACAGTCACACTACGCTTGACAGATGGGGGTCCCCAGCACCACTCCTGACCCTGTA
GCTGTGTTCCTCCATGATGCCCCACTGAGAACAGCTGGGACCCCTGCTTGCCAGGCCAGG
GGCCCAGCTTCTCTGCAGAAGTTCACACCTCTGAAATGCTTTCTTATTTCAAGACTCCCA
GCTACTTTTCAAAGAAAGAT
>S100a4|ENSMUST00000001046.6|Reference_end location: chr3(+): 90606040
site_id: 414568, AMPLICON
CCTTCCACAAATACTCAGGCAAAGAGGGTGACAAGTTCAAGCTGAACAAGACAGAGCTCA
AGGAGCTACTGACCAGGGAGCTGCCTAGCTTCCTGGGGAAAAGGACAGATGAAGCTGCAT
TCCAGAAGGTGATGAGCAACTTGGACAGCAACAGGGACAATGAAGTTGACTTCCAGGAGT
ACTGTGTCTTCCTGTCCTGCATTGCCATGATGTGCAATGAATTCTTTGAGGGCTGCCCAG
ATAAGGAGCCCCGGAAGAAGTGAAGACTCCTCAGATGAAGTGTTGGGGTGTAGTTTGCCA
GTGGGGGATCTTCCCTGTTGGCTGTGAGCATAGTGCCTTACTCTGGCTTCTTCGCACATG
TGCACAGTGCTGAGCAAATTCAATAAAAGGTTTTGAAA
```

>Scrg1|ENSMUST00000034023.3|Reference_end location: chr8(+): 57477581 site_id: 447511, AMPLICON
GATCGCAATTGTCACAACCTTCCGGAGGGCAGAGCCGACCTGAAGCTGATAGATGCAAAT
GTCCAGCATCATTTCTGGGATGGGAAGGGATGCGAGATGATCTGCTACTGCAACTTCAGC
GAACTGCTCTGCTGCCCAAAAGATGTCTTCTTTGGACCAAAGATCTCCTTTGTGATCCCC
TGCAACAACCACTGAGGATCTGCCTTGCACTCTGGAGAACATGGTCCTGAAGGCCTTCAC
GTCCCCTAATTTCCCACAAACTCTGTCAGTTCAGCGCCATTTCTGATATCCATCCAGTAT
ATCCAATCTTGCATAGATTCTATAAAGTCTTACTTGCTAGAGTATACTTGGGCTAAAGTG
GTAATAAAAGTTGTTTC
>Sema3g|ENSMUST00000090180.3|Reference_end location: chr14(+): 31230310 site_id: 1327400, AMPLICON
GTGGCATTTGGACAGAGTCTAGGTTTGGCAAGCTCTGGGCAGGGTCCTCTGGGGAAATGG
CCCTGGAAAACACAGGAAAAAGGAACTCGGTATCCTAAACAGTGCACCCCTATACCTGGA
AACATAGGACAGCTCTTGCCCACTGCCACCTTGCCCACGGTACTGTACTACGAACACCCT
GAAAACGCCCACATGTGTGAACTGTGTCTGTTTTCAAATGGGCCTTGTGGCTTCTCCGTC
TTCCAAGCATACttattgaattgttgttgatatttttattgtcatgttGCCTTGAGTTCT
GATGAGAAAAAGCAGAGCCT
>Sgms2|ENSMUST00000090246.4|PolyA_1 location: chr3(-): 131322738 site_id: 416694, AMPLICON
TTCCTGCAGCTCTTCGTTCATTGATGACAAAGCCCCCACACTGGAGTTCTGAAGAGGTGG
CAAAGAACACGCCGAGCCTCTCCTCGCCTTCCTTCACTTCCACGTTCTTTCCAGATTGCT
TTTTTTCTCCCTTCAAGGTCAGAAGAGTTTGCTAACGTTTTGAATAAAATGTCTGGATAT
ATAC
>Smpd3|ENSMUST00000067512.7|Reference_end location: chr8(-): 106252550 site_id: 1309357, AMPLICON
TTATTTGTCCAACACCACCTGGAGTTTTAAGTATGAATATTAACCTTGATGCTTTTTAAC
TATTGTATTAACTTGCTCAGATTTAGAAATACTGTTTTAAAAGACAAAGAAGAAACTTTT
TTAATTTCTGTATTTTTTTTCTGTATTGTATCCTATGGGACACTAGGGGTTTTATATGGT
AAGACACCCAAGGTTTTGGTAAAACATTATCAAGTATATATCCAGACAATTCTTCCCTAG
AAGAAAAAACAATCTCTATGCCTGATTTAAAAAAAAAGTCGAAAAGAGGTGGATTTTCCC
TTTATGGTGCTGAAAGGAAGACGGAGACTGAGGAGAAAATAAACCCGTGAGGAGGCTCA
>Smpd3|ENSMUST00000067512.7|PolyA_1 location: chr8(-): 106254960 site_id: 449646, AMPLICON
ATAGACCAGCCATGCCACGGTCTCGAGCAGTAGGGCCTCTGCCGGCCCTTCAAGCCACAG
CCCGTGCCTGGGCTGTGTGCCCTCCATTGAGCACCCAGTGCTGGGGGAAGGAGGGCAGGG
GACAGGGAAGGACGAGCCCATCAATCCCCTGCAAGCCTGGGAACCAGGCTGCTCTGTGCC
TCTGGGCCAACTCTGCGGACAGAGGACTTGGGCCAGCCTGGGAGCCCCCGGCTGCCTGAC
CAGTGCCATTCTTTCAGTCACGATTTTCTACAGAAATACACAGCACAACCTAGTTTGTAA
CCC
>Sox9|ENSMUST00000000579.2|Reference_end location: chr11(+): 112787750 site_id: 464858, AMPLICON
CTGCTGTGGCTGGAGAGTATAAGGAATGCttttttcttttttcttttcttttcttttttt
tttttttAAGACAGCAGTCTTTTTTTTTAATTTAAAAAAAAAAAGATATATTAACAGTTT
TAGAAGTCAGTAGAATAAAACCTTAAAGCGTTCTTATAATATGGCATCTTTCGATTTCTG
TATAAAAACAGACCTTTTAAAAAATATTTCTGTAACTTAAGAAACCTGACATTTATGTCA
TATTTTCTCTTTAGGTAAGATTTGGTTTGTTTGTGTCTTGTTTGTTTCCCTCTCCAAATT
CTTCCTTCTTTGTGCACCCTGCCTTTCTTCCCTTCCATCCTTTCTTTTTTGTATATTATT
GTTTACAATAAATATACATTGCATTAAAG
>Sox9|ENSMUST00000000579.2|PolyA_1 location: chr11(+): 112785920 site_id: 464859, AMPLICON
GACAACATATTGCAAATGGCCGGGCCACTCGTGGCCAGACGGACAGCACTCCTGGCCAGA
TGGACCCACCAGTATCAGCGAGGAGGGGCTTGTCTCCTTCAGAGTTAACATGGAGGACGA
TTGGAGAATCTCCCTGCCTGTTTGGACTTTGTAATTATTTTTTAGCCGTAATTAAAGAAA
AAAAAAGTC
>Spp1|ENSMUST00000031243.14|Reference_end location: chr5(+): 104441047 site_id: 1289114, AMPLICON
GGCTCTTAGCTTAGTCTGTTGTTTCATGCAAACACCGTTGTAACCAAAAGCTTCTGCACT
TTGCTTCTGTTCTTCCTGTACAAGAAATGCAAACGGCCACTGCATTTTAATGATTGTTAT

TCTTTTATGAATAAAATGTATGTAGAAACAAGCAAATTTACTGAAACAAGCAGAATTAAA
AGAGAAACTGTAACAGTCTATATCACTATACCCTTTTAGTTTTATAATTAGCATATATTT
TGTTGTGATTATTTTTTTTGTTGGTGTGAATAAATCTTGTAACGAAT
>Srgn|ENSMUST00000020271.12|Reference_end location: chr10(-): 62494461 site_id: 1317872, AMPLICON
CCCTTTCCTCATTAACTCGTAAGGAATTATGCTTTAATGCTGTTACCTATCTTGTTGTTC
TGGAAAATGCCTGCATTTATGTGTATTGAATCAACATTTAAGAAATTAACACACACCCCC
ATTATTATACAATAACTTTCAAAGCCATACTGGTTTTGAAAATTTTAATTTGATAGCAAG
TTGATGAACAATCTTTCATACCTAAAGTGTTCAGGAACCCAACTCGCATTGTGAATTACA
AATATATTCCTTTATGTGATTAA
>Stab1|ENSMUST00000036618.13|Reference_end location: chr14(-): 31139036 site_id: 471944, AMPLICON
GATGACTTCTCCCCATGGCAAGAAGGGACCAGCCCAACCCTGGTTTCTGTCCCCAACCCT
GTTTTTGGCAGCAGTGACATCTTTTGTGAACCCTTTGATGATTCAGTCCTGGAGGAGGAC
TTCCCCGACACCCAGAGGGTCCTCAAGGTCAAATGATGAGCTGGAATGAAGCAGAGGCTC
TGAGAAGACACAGACCATTTTTATTGCTTGTCAGGGTGGCTGAGGGGGCCAATGGTCTGT
CCAGGACAATAAAAGTGCCCTCAGCGGA
>Stc1|ENSMUST00000014957.9|PolyA_1 location: chr14(+): 69041321 site_id: 1330851, AMPLICON
CTAGACCTGTTTGCATGATCTCCTCTCTTGTGATTTGGTTGCACTTTAGAACATTTTTGT
GCCGTATTATTTGCATTATGTATTTATAATTTAAATGATATTTAGGTTTTTGGCTGAGTA
CTGGAATAAACAGTGAGCATATCTGGTATATGTCATTATTTATTGTTAAATTACATTTTT
AAGCTCCATGTGCATATAAAAGTTATGAAACATATCATGGTAATGACAGATGCAAGTTAT
TTTATTTGCTTATTTTTATAATTAAAGATGCCATAGCA
>Tagln|ENSMUST00000034590.3|PolyA_1 location: chr9(-): 45930003 site_id: 1312216, AMPLICON
TAGCCTGCCTCACAAATGCCTATGTAGGTTCTTAGCCCTGACAGCTCTGAGGTGTCACTG
GGCAAAGATGACTGCACATGGGCAGCTCCCACCTATCCTTAGCCTCAGCCCAGCATCTTA
CCCCAGAGCCACCACTGCCCTGGCCCCTGTTCCCAGCTGTACCCCCACCTCTACTGTTCC
TCTCATCCTGGAGTAAGCAGGGAGAAGTGGGCTGGGGTAGCCTGGCTGTAGGCCAGCCCA
CTGTCCTTGATATCGAATGTCCTTTGAAGGAGACCCAGCCCAGCCTCTACATCTTTTCCT
GGAATATGTTTTTGGGTTGAAATTCAAAAAGG
>Top2a|ENSMUST00000068031.7|Reference_end location: chr11(-): 98992994 site_id: 463882, AMPLICON
AACTGGTTAGTTCTAGTACAGATACAGTGCTCAACCTCTGACGTGATGCATTTTGTTTAA
GCCATGAGAAGTTGCTCGTACCTTTTGAACATCGGAGGCTGGAGAAAGTCACCTTGACTG
TGTCTTTATGACTCAGCACAAGCAGCAAGGTGAGGAAAGTTAGGTCAGTTACGTAGAGTA
CTGACTCTACTCGGGATGCCTTCCCAACACGAGATCCCTGGGAGGAGGCAGGACTGCTCA
CGGTTACATCTCCAGGATCAACCAGGAAGTGGGATCCATTGCAGATCTCCATAATTCCAA
CTTGTGACTGCCAAGACCTTCTGTGCTGTTGTCTCTTATTTAAGTGCTGTTATCAATGTC
TTTTGTAAATATTTAATATGTCTGTCTGTTCCGC
>Vcan|ENSMUST00000109543.8|Reference_end location: chr13(-): 89657056 site_id: 470552, AMPLICON
GTTTCATTCAGCGACACCTTCCAACTATCCGGTGCCTAGGAAACGGGAGATGGGCAATGC
CTAAAATAACCTGCATGAACCCATCTGCATACCAAAGGACTTATTCTAAGAAATACTTAA
AAAATTCCTCATCCGCAAAGGACAATTCTATAAATACATCAAAACATGAGCATCGCTGGA
GCCGGAGGCGGCAGGAAACCAGGCGCTGATCCTTAAAATGGCGAACATGTGCTTCACTCA
TCATTTCAGCCAAAGTCCTGCCTTTCCGTGCCTTTCCTATCACCTCAAGAAGAATTAGAA
GTTGGTTTGGATTATGGGACTGCCGTCTGGTCATTTGGGGTGGCTGTACTCTTAAAATAT
TTTCAATGAAACATGGAATTTTGA
>Vwf|ENSMUST00000112254.7|Reference_end location: chr6(+): 125686672 site_id: 1296323, AMPLICON
AGCAAAGCTGTGTACTCCATTGACATCGAGGATGTGCAGGAGCAATGCTCCTGCTGCCTG
CCCTCGAGGACGGAGCCCATGCGCGTGCCCTTGCACTGCACCAATGGCTCTGTCGTGTAC
CACGAGGTCATCAACGCCATGCAGTGCAGGTGTTCTCCCCGGAACTGCAGCAAGTGAGGC
CTGTGCAGCTACAGCGGATTCCTACTGATACCCATGCTATGACCTGGCTATCCAGAAAAC
CAGATCCCTCCCTTGGGCCTCCTGACCTTTGTGCCCTCCTGGGCCCACAATAAAGGCCAA
GTTCTCATC

>Wls|ENSMUST00000068952.9|PolyA_1 location: chr3(+): 159935051 site_id: 1280494, AMPLICON
CTTTGCAGTCCAACATTTGCTGATGTCATAGTCTTTTTACTCTCCTCCATGGGCACTTTT
TTCTATTTATATCACCAAGTAGCCAAATGAGTTATTTAAGTTGTCAGTACAAATATGTCA
ACTTTTTAAAAAATGGATTACTATAAAATAACCAAACTTCATGAGAAATTTTATCTTACC
AGAGGCATTTCAGCTTTGGAACCAAATCTGTGTATTTGATACTAATCTGTCTGTTTGTTT
GTGGATTTTGAGAAATATTTGCTTAACTACCCAAATAGGATTTCTGGTATTAAATGG
>Wnt1|ENSMUST00000023734.7|Reference_end location: chr15(+): 98793837 site_id: 476702, AMPLICON
CCACGCGTTCGGTTATGTAAATAAAACTATTTATTGTGCTGGGTTCCAGCCTGGGTTGCA
GAGACCACCCTCACCCCACCTCACTGCTCCTCTGTTCTGCTCGCCAGTCCTTTTGTTATC
CGACCTTTTTTCTCTTTTACCCAGCTTCTCATAGGCGCCCTTGCCCACCGGATCAGTATT
TCCTTCCACTGTAGCTATTAGTGGCTCCTCGCCCCCACCAATGTAGTATCTTCCTCTGAG
GAATAAAATATCTATTTTTATCAACGA
>Wnt10a|ENSMUST00000006718.14|Reference_end location: chr1(+): 74804171 site_id: 1263961, AMPLICON
CGTCATCGGGCTGAAGTGACTCTAAGGGCTCCAGACCTCTGCTCCTGTCTTTCACTTAAC
AGATATTTATTTTTGCGCTCTCTTTGAGACACTCTCTGGGGAAAAAGAAGCTCCggagtc
tacaggctgattaagggacatggacaataaaccagtaaaca
>Wnt10a|ENSMUST00000006718.14|PolyA_1 location: chr1(+): 74803900 site_id: 399696, AMPLICON
TGGGTCTCAAGAATGGTTGTCCTCTTGGTGCCTGGCTTCTGCCGCTAGCGGATCTGAGCC
AGGCAGCAAGCAGCAGCCTTGGCTCCTGAGAGAGGTGGTTGGCTCTTACAGCCCCGAGGG
TCTACAATCACCAGACAGTCCAGATCTGATTGACATTCCTCCGCTCACCTCTGTAGGTTC
CCCTCTTTCTGTTCCTAGCTCAGACAGCTGGGGGTGATAGTGGAGACTGTTCCACACCCT
AGGACAGGTCACCAAAGCAGCCC
>Wnt10b|ENSMUST00000023732.11|Reference_end location: chr15(-): 98771752 site_id: 476697, AMPLICON
GGTTCTCAAACTCCTCCACTACAGCCCAGAACCTCCTCTTATGGGACCTCGGGTGACAAT
AATGAGAGGTTTTCGGTTGGAAAAGGACAGAGGGCAGGGAAGCCTCAGACAGCTGTCTTG
TCAGGCTCTTGGGAGGCTTCTCCTTCCGTTCAGTTGTTGAAAGGGTCTCTCCAAAGGAAA
GGTTTTAGCCATAACTCTTGGAGGCCCTTTTCCTTCTTCAGCAGGAAGGGTGGGAATGGA
TAATTTATTTTACTGAGATGTGTTCTTGGTTCCTGTTTGAAACTAAAATAAATTAAGTTA
CTGAACTACTGG
>Wnt10b|ENSMUST00000023732.11|PolyA_1 location: chr15(-): 98772238 site_id: 476698, AMPLICON
TCGTTTCCACTGGTGCTGTTATGTGCTGTGTGATGAGTGTAAAGTCACAGAGTGGGTCAA
TGTGTGTAAATGAAGGTGAGCCTCGCCTAGGCACGACGAGGAGGAGAAGCACTGTGTGAG
GGCTGCTCTCTTTCAGCCCTTTGCTCGGATTTCTGTCTAGGGTTTATCGTGGCTCCCGGA
AGCTCAGAGCATCTGCCTGAGAACAGCTCTGGGGGTGTAGGGTCAGGTGAAATCTGTAAC
GAGCAGCCTTTTGTGGGGGAAGTGGCCCCACACTCTGTTCTTAAACACTCGAATAGACTA
AGATGAAATGC
>Wnt11|ENSMUST00000067495.8|PolyA_1 location: chr7(+): 98853811 site_id: 1302580, AMPLICON
CCACATGGAACCACTAACTTGGGTTGTAAATTTTTATTTTCCTTCCCCTCTCCGTGGGAT
GTGGGAGTTACAGAAATATTTATAAAAATACAGCTTTTTCTTTGGGGTGGCTCACTTCAA
TCCCTCTTtatatattatatatataaatatatataatgattatatatTT
>Wnt16|ENSMUST00000031681.9|Reference_end location: chr6(+): 22298522 site_id: 431083, AMPLICON
CGTGGAGAGGTGTGAGTGTAAGTTTATCTGGTGCTGCTACGTCCGCTGCAGGAGGTGTGA
AAGTATGACCGATGTCCACACGTGTAAGTAACCTCTCCGTCCAGCCTAGCATGAGACGCC
TCTGTAGTAACCAAGGTGTGGTGTTGGCATCTGGAGGGCGCCCCTACTGTGCACTGATGG
GGAAGTCGCTGCCTGTAAGAGTGTTCCCAGACCCCTGGGCTAGTCTACGATTTCTTTCTT
TCTGGCAGGCTTCAAATCACAAGCTGATCCAGAGGATTGCTTGGGATTCTGAAGTTGAAA
AGGTTGGCAGTCGCCTTTGGATGATTTGGGAAATATACATTGATATACAGGAAACATCAA
ATCTGTTTCTGAAGCAATGTGG
>Wnt2|ENSMUST00000010941.5|PolyA_1 location: chr6(-): 17989246 site_id: 431013, AMPLICON

CACCCTATTCTGTCTATCTTGGGCATTCTGATGTCACCTCTCTTCCTGCTGATTTCTTTT
TGGAAATGGCATGACAGGCTGTTAGAGGAGGAGGGTCATAGCCCCCCACCACTGTCACCT
AGACATTTCCTCTTTGGCTGCGGGGAGAAACATCACATAGCGAAGGAACTTCCTCTGTGT
TTTCCCAGATTCCAACAACCCAGAAAGTCTGTGTTTCCCTGGGGCGCGGGGTAGGGATGG
AAAGCAGAATGAGCTGACACCAAAATTTCCTCGGATTTTTTAAAAAAAGAGTAAGCAAG
GGCTTTAACTAAGTGATAGCTGTTGATAGCATCCTTGGTGACTTTCTAGAGAAAGATGGC
TTCCAATAAACATCAGGTTAAAAC
>Wnt2b|ENSMUST00000029429.5|Reference_end location: chr3(-): 104945272 site_id: 415647, AMPLICON
CCGACTGATAGACGTCATCAACACTCTCACTGGTCAAGTACTTCCTGCTTCTCTGGGACC
TTCTGATTTAGGGCTGTCTGGGCAGACAACAGACTAGATTCAAAGGCTTTCACAAGGAAT
TCTGGATATAGCTCCTCTCTCTCTTCTCAGGTTCCTCTTCATCCAATCGTACTCTCAGAT
GTTTGTGGAGCAACCTCTTTCTGCCCAGGCAGCAGGAggctggggtggggtggggtgggg
gggCACAGCTCTGGCCACAGAGGCAGATTTATTTGGATGATAGGACTAATATTTGTGTAA
CCTGCTG
>Wnt3|ENSMUST00000000127.5|Reference_end location: chr11(+): 103817957 site_id: 464453, AMPLICON
AAGTCATGTTTCCGGGGCGTATTCAAGTAGCTGACAAGTAATTATTTAATAATAGTACAT
GAGCGCATTGTAATTATCCTCGCCATAGTCAGGTAATAGCATCCAATGGGAGGTCCCTAC
CAACCTGCTGTATCCAAAGTTTTGTAAAAAGTTGTAGAAGTTGTTGATCTTTTTGATTTT
ATATTCAAAAAGTCTCTTTTTATAAATATTATTTATTATACAATGTATATACCTTTGAGT
TAACTAAGATtatatattatataaatatatatatatT
>Wnt3a|ENSMUST00000010044.7|Reference_end location: chr11(-): 59248033 site_id: 461067, AMPLICON
TCTGCCACAAGAGCTTCCTGATTGGTACCACTGTGAACCGTCCCTCCCCCCTCCAGACAG
GGGAGGGGATGTGGCCATACAGGAGTGTGCCTGGAGAGCGCGGAAAGAGGAAGAGAGGCT
GCACACGCGTGGTGACTGACTGTCTTCTGCCTGGAACTTTGCGTTCGCGCTTGTAACTTT
ATTTTCAATGCTGCTATATCCACCCACCACTGGATTTAGACAAAAGTGAtttctttttt
tttttttcttttctttCTATGAAAGAAATTATTTTAGTTTATAGTATGTTTGTTTCAAAT
AATGGGGAAAGTAAAAAGAGAGAAAAAAAAATAA
>Wnt4|ENSMUST00000045747.4|PolyA_1 location: chr4(+): 137299497 site_id: 422350, AMPLICON
CTTCCTACCTGAACCACTCTCTACTGTTGTTGTCACAAGGCAAAAGTGGCATTCCTTCCT
CCAAGGCCAGGGCTGGAAAAGCCAGCTCCGGATGCTGCTACTCTACCGTGCTGCCCCACC
CCGTCCTCCAGTTTTTATATGAGGCTCCTTGGCATTGTGACATCCCCACGGCATAGTCTG
AGATGCACGATAAAAATGGTTATTTCTC
>Wnt4|ENSMUST00000045747.4|PolyA_2 location: chr4(+): 137296856 site_id: 422351, AMPLICON
CGTGCAACAAGACATCTAAAGCCATTGACGGCTGCGAGCTACTGTGCTGTGGCCGCGGCT
TCCACACAGCGCAAGTGGAGCTGGCCGAGCGCTGTGGCTGCAGGTTCCACTGGTGCTGCT
TCGTCAAGTGCCGGCAGTGCCAGCGGCTCGTGGAGATGCACACGTGCCGGTGACCATGCC
GTCTGTGCCCAGGACCACCTGCGTGGCCCAGGGAAGGCCAATAACTTAAACAGTCTCCCA
CCACCTACCC
>Wnt5a|ENSMUST00000063465.11|Reference_end location: chr14(+): 28527440 site_id: 1327366, AMPLICON
TTCCCTCTCTTGGTGGAGCTGTAAACAAGATGGCATGTTGTGAAGGTTCAAGATGATTTT
TTTTTAAATCGCAGAAACATTTAGACACCTAAGAACTAAAACTTATAAAAGGGATCTTTG
AATTTGCCTGTTAACATGGATTAATGTTTACACTTACAGCTGATGATTGGACGGTGTTTT
ATGTTAGGGAAATGCCTTGTTAACGAACTTCATGAAGCAGATGTAATTAAAGGTTGATGT
GAGCCAATCTAGAAGGTTGAACAGTGTTTTCAAAGAACGGAGAGACTTACATTTTAGACC
AATCTTTATACATTTTGCTGAGCTAGAAAGGAGATAAAGATTATTTATTTTTGTTCATAT
CTTGTACTTTTCTATTAAAATCATTTTA
>Wnt5b|ENSMUST00000117171.7|Reference_end location: chr6(-): 119432535 site_id: 434942, AMPLICON
GCTCCTGCCTCAAGCTCTTGTGCCAAGAGAAAGACGGTTCTGTCACCTGCTACAGCCAGG
AAGACGTGGAGCAAACCTGGGATTTGACTGGGGACCAAGTGCCTGTTGCACAGGACAGGA
ATCTGCTGTCACTCTGTCAAGGGAGGCTTTGAGAATGACAGGGCATGCTAGCAGGTCAGG
TCAACTGCCTGTGAGACTGTCATCTCTGCCCACATGTACAGCGTCCCTTTGACATTAAAT

ATCTTTTTACTG
>Wnt6|ENSMUST00000006716.7|Reference_end location: chr1(+): 74785319 site_id: 399664, AMPLICON
ACCTTTAGCACACCTTCTGTTCTTTCTTGGCTCTCGAGAACTTGTGGTCCCATGTTAAAG
GAACACAAAGAGGCCAGAAGCTTCTAGTAGTGGGCTTAGTTGTCAGGCGTCTGTGTGAAC
TTTTATGGCATTCTGTCTTTCTGTGACTCCTGATGAAATTAACGGTCATGAGATTAAAAG
CTCGTCATGAACTGTAAAATGCAAC
>Wnt6|ENSMUST00000006716.7|PolyA_1 location: chr1(+): 74784945 site_id: 399665, AMPLICON
TGAAAGGCCCTTTGCTAGTTCCTGCAGGAGATCACTCCCCTTGGCCCCCCAGATGGAAAT
AAAAAAGCCAGACTCTGCCCTCTGGAAATAATATTCCTCAGAATTACTGGGATGGATGGG
TGAGTTTAGTATCAATAAAGACATTTAAATCC
>Wnt7a|ENSMUST00000032180.6|Reference_end location: chr6(-): 91363995 site_id: 434093, AMPLICON
CACTTGTGGTCTCAGGGGTTATTTCCAGGTATCTGCATTTGTGGGTGGGGTGCAAGGTAG
ACAGCAGGGAACTGATTTGATTGTGTTGAGCCACAGTGAGACTGCAACTCTGAACTCTGT
CTCCACAGCTGCTGGTGAAACTCAGATGCCTGTGAGACAACAGCCCTGAGCCTCATGGCC
CACATGCTGGGAGCCCCTCAGTGTCTAGGTCATGTCCAGTCCCCCACCTGGGTTACATCA
CGACCAATAAACATGGCTGTATGGCTGAT
>Wnt7b|ENSMUST00000109424.3|Reference_end location: chr15(-): 85535439 site_id: 1330624, AMPLICON
CCCTGTCTGTCATGTCTCTTAAGTTATTGTGACCTACACTGGGTACCGGAGGGGATGGGG
GATGGCTTCAGCTGCTGTCCCCCAAGCCAGGCTCCTCCTTCTGCTTGAAACAGACCCTCG
GGGGCCCCTGATGCCACCGAGGCAATTCGCACTGTCCCTGGGCTGCCAGGCACCTGCGCC
TGCACTCGGTCAGCCGCAGACCTTGCCTTGGGGGAGAGAGGTGGTTAGTGGACCCAGGCA
GGGCACTGGCTGTCCCAATGCTGTGTGCTGGGGTGGAGGTGGCCGGGCACCACATGTCCT
TGAAGTGCCCTACTTCTGATGGGCTGTGTTCCTGCCTCCTCTGGAGGGGAGCACTTAGCC
CCAATAAAAGCTGGAATCAGAAAAGCA
>Wnt8a|ENSMUST00000012426.2|Reference_end location: chr18(+): 34548273 site_id: 483847, AMPLICON
CTTTCCTGCGCAGTTACTCTTGGACTTAAGCAGCTTGTTAAAGAGGGAGTTTGATTTGGG
TGCACATTCAGAGGAGCCTGGAAGAACCGTATTCCATTAAGTTTCAGATACCGTTCCACC
CAGCTGTGCTGCTGGGAGTGCGAGGGAAGAGAAGTTAAAGGAAAGGAATTCTGggggcgg
gagagatggctcagtggttaagggccctggctggccctccagaggactggctcacttcac
agcacccacttgatggctgtgagccatctgtacttctagttccaggggatccaatgtcct
tgcctggtctctgtgaccaccaggcacaaatgtgcacagacagacatttatacatataaa
ataataaagtaaaAACTTACATTT
>Wnt8b|ENSMUST00000041163.4|Reference_end location: chr19(+): 44514273 site_id: 486906, AMPLICON
ACTCCCAGTTCATCCCAGTTCTACAGAGAACTTCCTGCCCATGCACTACGCCTTCAGCCT
TTGTTCACGCTATACACACTGGAGTGGAGACAGCTAACAACACACAAGCATCCTTCAGTT
CACGGCTTCATGTCCTACACTAACTCACTGAGACTCTGCAGACAACTCTAGAGCTGCTGA
CTGCTGACTGGGAAGATTAGACACCTCTGAGGTCAGCTAGTCAAACTTTaac
>Wnt9a|ENSMUST00000108783.3|Reference_end location: chr11(+): 59333542 site_id: 461069, AMPLICON
GTCTTTCTGGATGAAGCAACTCCCGCAGGCCAGAGCCTCTTTGATCTTCTTCTATGGGCA
GCAGAGTGAGCAGCTGCCCTGCTCCCGCCGGTCCTGCCGGCCACCCAGGTTAAGCCGGTA
CAGCAGTGGACTCACAAGGGAGCTGTCTTTGTGTGGAGTCTTGTGCAGCCTGTGATTTCT
CTACCTCAACTGTGTGGCGAGTAGGTGTGAACCTGAGTGACTGCAGAGAATGTATTTATT
TAACAGCTTTGTGTAACAAAACCAGAAACAAATGGAAACACTAAATAAATGTATTTTAAA
TT
>Wnt9b|ENSMUST00000018630.2|Reference_end location: chr11(-): 103727364 site_id: 464452, AMPLICON
TCTCAACACCCATGAACGTCCATGCTTCCTGTCTGAGCACTGAGGAGAACCCCAGCGGAG
CTCATTGTTCAGTGCTGGAATACCCATCCCCCCTCCCGTTGATtatttagggagtgtctg
ataatgccaggggatactctgggtgctagggcgcagaagtacttaagagcaagtcccagc
ctcaggggacttatatgccggcgaggagaaagccaacaaaccaataaaCTATGCACTGGT
