# Supplementary material for: A specialized bone marrow microenvironment for fetal haematopoiesis
Source: Nat Commun. 2022 Mar 14;13:1327. doi: 10.1038/s41467-022-28775-x (PMC8921288; doi:10.1038/s41467-022-28775-x)
Supplement: Supplementary file 8 — Reporting Summary [file 41467_2022_28775_MOESM8_ESM.pdf]

Corresponding author(s): Ralf H. Adams

Last updated by author(s): Jan 24, 2022

Reporting Summary

Nature Research wishes to improve the reproducibility of the work that we publish. This form provides structure for consistency and transparency in reporting. For further information on Nature Research policies, see our Editorial Policies and the Editorial Policy Checklist.

Statistics

For all statistical analyses, confirm that the following items are present in the figure legend, table legend, main text, or Methods section.

n/a	Confirmed	
☐	☒	The exact sample size (*n*) for each experimental group/condition, given as a discrete number and unit of measurement
☐	☒	A statement on whether measurements were taken from distinct samples or whether the same sample was measured repeatedly
☐	☒	The statistical test(s) used AND whether they are one- or two-sided<br>*Only common tests should be described solely by name; describe more complex techniques in the Methods section.*
☒	☐	A description of all covariates tested
☐	☒	A description of any assumptions or corrections, such as tests of normality and adjustment for multiple comparisons
☐	☒	A full description of the statistical parameters including central tendency (e.g. means) or other basic estimates (e.g. regression coefficient) AND variation (e.g. standard deviation) or associated estimates of uncertainty (e.g. confidence intervals)
☐	☒	For null hypothesis testing, the test statistic (e.g. *F*, *t*, *r*) with confidence intervals, effect sizes, degrees of freedom and *P* value noted<br>*Give P values as exact values whenever suitable.*
☒	☐	For Bayesian analysis, information on the choice of priors and Markov chain Monte Carlo settings
☒	☐	For hierarchical and complex designs, identification of the appropriate level for tests and full reporting of outcomes
☒	☐	Estimates of effect sizes (e.g. Cohen's *d*, Pearson's *r*), indicating how they were calculated

Our web collection on statistics for biologists contains articles on many of the points above.

Software and code

Policy information about availability of computer code

Data collection	Leica SP5 application Suite Advanced fluorescence2.6.3.8173; LSM780 ZEN 2011 SP7 FP1 14.0.9.201; FACSuite v1.0.5.3841; FACS Diva8.0.1; FACSymphony A3 Cell Analyzer:
Data analysis	Fiji(Image J 1.51s), Volocity(6.3), Photochop(CS6), Illustrator(CS6), Graphpad Prism(7), Microsoft Excel(15.40), FACS Diva(8.0.1), FACSuite (1.0.5.3841), FlowJo(10.4.2) were used for data analysis. Sequencing data were processed with UMI-tools (version 1.0.1), aligned to the mouse reference genome (mm10) with STAR (version 2.7.1a), and quantified with Subread featureCounts (version 1.6.4). Data normalization and further analysis were performed using Seurat (version 3.1.3). Monocle (version 2.12.0) was used for pseudotime trajectory analysis. Cell cycle phases were classified by cyclone function of scran (version 1.14.5). An R package iTALK (doi: https://doi.org/10.1101/507871) (version 0.1.0) was used for the ligand-receptor interactome analysis. MetaCell (version 0.3.6) was used to compute cell to cell similarity.

For manuscripts utilizing custom algorithms or software that are central to the research but not yet described in published literature, software must be made available to editors and reviewers. We strongly encourage code deposition in a community repository (e.g. GitHub). See the Nature Research guidelines for submitting code & software for further information.

Data

Policy information about availability of data

All manuscripts must include a data availability statement. This statement should provide the following information, where applicable:

- Accession codes, unique identifiers, or web links for publicly available datasets
- A list of figures that have associated raw data
- A description of any restrictions on data availability

The raw data of scRNA-seq has been submitted to GEO, and will be made public available when manuscript is accepted. Data supporting the findings of this study are available within the article and from the corresponding author on reasonable.

Field-specific reporting

Please select the one below that is the best fit for your research. If you are not sure, read the appropriate sections before making your selection.

- [x] Life sciences
- [ ] Behavioural & social sciences
- [ ] Ecological, evolutionary & environmental sciences

For a reference copy of the document with all sections, see nature.com/documents/nr-reporting-summary-flat.pdf

Life sciences study design

All studies must disclose on these points even when the disclosure is negative.

Sample size	No statistical method is used to predetermine sample size. Sample sizes were chosen based on previous publication from our lab and other research on haematopoiesis (Reference PMID: 30661958, 33947846, 31761723), roughly 6 to 20 mice were analyzed for each sample.
Data exclusions	Mice that died before the completion of experimental protocols were excluded from analysis, which was a pre-established criterion before the experiment. Embryonic mice with clear developmental delay or unexpected death were excluded from analysis.
Replication	All attempts of replication were successful to get data point for statistics. At least 2-3 experiments were performed independently.
Randomization	Mice were allocated into each group based on their genotyping results
Blinding	Samples were allocated after we get genotyping result, therefore investigators were blinded to group allocation in many analysis.

Reporting for specific materials, systems and methods

We require information from authors about some types of materials, experimental systems and methods used in many studies. Here, indicate whether each material, system or method listed is relevant to your study. If you are not sure if a list item applies to your research, read the appropriate section before selecting a response.

Materials & experimental systems

n/a	Involved in the study	
	x	Antibodies
x		Eukaryotic cell lines
x		Palaeontology and archaeology
	x	Animals and other organisms
x		Human research participants
x		Clinical data
x		Dual use research of concern

Methods

n/a	Involved in the study	
x		ChIP-seq
	x	Flow cytometry
x		MRI-based neuroimaging

Antibodies

Antibodies used	Antibody information including supplier name, catalog number and usage are provided in Supplementary Table S2
Validation	Antibodies were validated using FACS or immunostaining. The antibodies were mostly also validated by the Manufacturer. Validation of antibodies can be checked in figures of manuscript.

Animals and other organisms

Policy information about studies involving animals; ARRIVE guidelines recommended for reporting animal research

Laboratory animals	Details of laboratory animals are described in the "Animal experiments and genetically modified mice" section of Methods. Infomation about genetically modified animals were summarized in Supplementary table.1. The strain of mice were described in the "Animal experiments and genetically modified mice" section of Methods. For mice mating, we used 8-30 weeks old mice. We used both gender of embryo mice for analysis. The animal house has 14/10 hours light/dark cycle at approxiamte 25 degree centigrade.
Wild animals	The study does not involve wild animals.
Field-collected samples	The study did not involve samples collected from field
Ethics oversight	All the animals were housed in the animal facility at the Max Planck Institute for Molecular Biomedicine. All experiments were performed according to the institutional guidelines and laws, following the protocols approved by local and national animal ethics committees. The permission is granted by permissions granted by the "Landesamt fuer Natur, Umwelt und Verbraucherschutz (LANUV) of North Rhine-Westphalia", Germany.

Note that full information on the approval of the study protocol must also be provided in the manuscript.

Flow Cytometry

Plots

Confirm that:

- [x] The axis labels state the marker and fluorochrome used (e.g. CD4-FITC).
- [x] The axis scales are clearly visible. Include numbers along axes only for bottom left plot of group (a 'group' is an analysis of identical markers).
- [x] All plots are contour plots with outliers or pseudocolor plots.
- [x] A numerical value for number of cells or percentage (with statistics) is provided.

Methodology

Sample preparation	Bones were dissected to remove muscles and then crashed by pestle for 3-4 times. All the cells were collected in 2% FCS-PBS solution. The tissue was immersed in 6ml dissociation solution (2% FCS-PBS solution with approximate 145U/ml type 4 Gibco collagenase) and incubated at 37 degree water bath for 30 minutes. The samples were filtered using 70µm Nylon cell strainer to get single cell suspension solution for staining or directly sorting.
Instrument	FACS sorting was performed on a FACSAriaIIu cell sorter (BD Biosciences, San Jose, CA) using a 85 µm nozzle. FACS analysis was performed using BD FACS Verse.
Software	FACS sorting data was processed in FACS Diva8.0.1. The representative image was exported to fcs file and processed in flowJo (10.4.2). FACS analysis data was directly processed in FACSuite(version1.0.5.3841).
Cell population abundance	In FACS analysis, the abundance of relevant cell groups(e.g. LSK or HSCs) were in general consistent with previous publication. The LSK/HSC abundance is approximate 0.1% and 0.01%, respectively. In post-sort fraction, the purity of cells are higher than 90%, which is determined by FACS analysis of post-sort sample
Gating strategy	For all flow cytometric analysis and sorting, debris were excluded by FSC SSC scatters. DAPI staining was used to exclude dead cells in all sorting experiments. Unstained control and FMO (fluorescence minus one) control were used to define boundaries of positive and negative staining cell populations. For genetically modified mice, littermate control were used to define boundaries of positive and negative cell populations.

- [x] Tick this box to confirm that a figure exemplifying the gating strategy is provided in the Supplementary Information.
